# Synthesis, Antiprotozoal Activity, and Physicochemical Evaluation of Benzamido–Menadione Derivatives

**DOI:** 10.3390/ijms262210951

**Published:** 2025-11-12

**Authors:** Armin Presser, Gregor Blaser, Eva-Maria Pferschy-Wenzig, Monica Cal, Pascal Mäser, Wolfgang Schuehly

**Affiliations:** 1 Institute of Pharmaceutical Sciences, Pharmaceutical Chemistry, University of Graz, Schubertstrasse 1, 8010 Graz, Austria; gregor.blaser@gmx.at; 2 Institute of Pharmaceutical Sciences, Pharmacognosy, University of Graz, Beethovenstrasse 8, 8010 Graz, Austria; eva-maria.wenzig@uni-graz.at (E.-M.P.-W.); wolfgang.schuehly@uni-graz.at (W.S.); 3 Field of Excellence BioHealth, University of Graz, 8010 Graz, Austria; 4 Swiss Tropical and Public Health Institute, Kreuzstrasse 2, 4123 Allschwil, Switzerland; monica.cal@swisstph.ch (M.C.); pascal.maeser@swisstph.ch (P.M.); 5 Faculty of Philosophy and Natural Sciences, University of Basel, Swiss TPH, Petersplatz 1, 4003 Basel, Switzerland

**Keywords:** antiplasmodial, plasmodione, menadione, physicochemical parameters, ligand efficiency metrics

## Abstract

The naphthoquinone skeleton is known for broad biological applications and, in particular, for antiparasitic efficacy. As part of our ongoing search for new antiprotozoal naphthoquinone derivatives, we incorporated computer-aided optimization models utilizing physicochemical parameters into our approach. Herein, we report on the synthesis of 21 new benzamido–menadione and naphthoquinone derivatives via the Kochi–Anderson reaction. The antiprotozoal activity of all the synthesized compounds was evaluated against *Plasmodium falciparum* NF54 and *Trypanosoma brucei rhodesiense* STIB900. Cytotoxicity towards L6 cells was also determined, and the respective selectivity indices (SI) were calculated. Several ligand efficiency metrics, such as LLE, SILE, and FQ, were calculated, and the results were visualized in scatterplots. Almost all of the synthesized benzamido–menadione derivatives exhibited high activity against NF54 (IC_50_ < 1 µM), with the strongest activity and excellent selectivity observed in the 2-fluoro-5-trifluoromethylbenzamido derivative **2f** (IC_50_ = 0.021 µM, SI = 10,000). Specific ligand efficiency metrics, such as SILE, LLE or FQ, showed a clear correlation with the corresponding antiplasmodial activities. Toxicity predictions confirmed low acute oral toxicity for most compounds, further supporting their potential as safe drug candidates. Our findings highlight the benzamido–menadione scaffold as a viable option for new antiplasmodial drugs.

## 1. Introduction

Malaria, the world’s most devastating parasitic disease, remains a serious health threat. In 2023, the World Health Organization (WHO) estimated 263 million cases and 597,000 deaths globally, a rise of 11 million cases from 2022, with most occurring in the African region [1,2]. Progress in the global fight against malaria has stalled slightly in recent years. Therefore, the WHO is requesting increased efforts and additional funding to support the strategic use of new tools, including vaccines and new antimalarial drugs.

**Figure 1:**
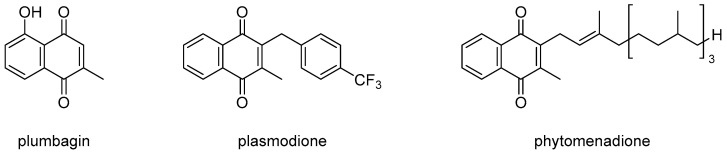
Alkyl-1,4-naphthoquinones with remarkable biological activities.

Alkyl-1,4-naphthoquinones, including plumbagin, plasmodione, and phytomenadione (vitamin K1) (Figure 1), have attracted considerable attention due to their significant biological activity [3,4,5,6,7]. In particular, the redox-active compound plasmodione has emerged as a promising candidate in the search for new malaria drugs due to its multifactorial mechanism of action against different stages of the malaria parasite *Plasmodium falciparum* with varying degrees of drug resistance [8,9,10,11]. This compound belongs to the class of 1,4-naphthoquinones, which are recognized for their antiparasitic efficacy and broad biological applications [12,13,14,15,16]. In addition, plasmodione has demonstrated a favourable safety profile, which enables a possible human use, including for patients with G6PD deficiency [17].

In the present study, 21 plasmodione-like derivatives with a benzamido–naphthoquinone skeleton were synthesized. The strategic application of physicochemical parameters is of particular importance in this work due to their decisive role in rational drug development. Therefore, in order to assess the usability of the synthesized compounds, an analysis of various physicochemical parameters and ligand efficiency metrics was conducted, as well as toxicity studies.

## 2. Results and Discussion

### 2.1. Synthetic Chemistry

We recently reported a simple and fast preparation route for benzyl-substituted 1,4-naphthoquinone (NQ) and menadione (MD) derivatives via the Kochi–Anderson reaction [18]. Silver-catalyzed coupling between an NQ core and substituted phenylacetic acids is an efficient method for synthesizing these derivatives in a single step [9,19,20]. However, it has been demonstrated that the use of aminophenylacetic acids as coupling agents yielded only very modest results, and in addition, it requires the application of protective groups [21].

**Scheme 1:**
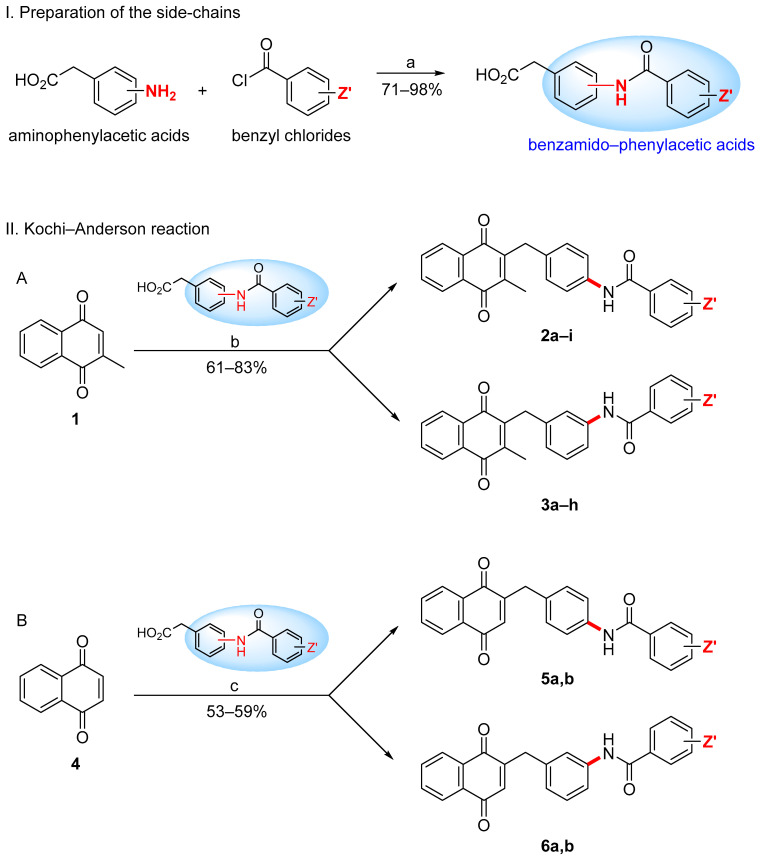
Synthesis of benzamido–menadione and naphthoquinone derivatives via Kochi–Anderson reaction. Reagents and conditions: (**I**) aminophenylacetic acid (1 equiv.), benzoyl chloride derivative (1.1 equiv.), 2 M KOH, rt, 18–24 h; (**IIA**) menadione (1 equiv.), phenylacetic acid derivative (1.4 equiv.), AgNO_3_ (0.35 equiv.), (NH_4_)_2_S_2_O_8_ (1.3 equiv.), CH_3_CN/H_2_O, 85 °C, 3–6 h; (**IIB**) 1,4-naphthoquinone (1 equiv.), phenylacetic acid derivative (1.4 equiv.), AgNO_3_ (0.1 equiv.), (NH_4_)_2_S_2_O_8_ (2 equiv.), CH_3_CN/CH_2_Cl_2_/H_2_O, 80 °C, 3–4 h.

In the present study, we employed a variety of benzamido-phenylacetic acids as reactants, which were synthesized from commercially available aminophenylacetic acids and (mainly fluorinated) benzyl chlorides as protecting groups (Scheme 1), according to Billamboz et al. [22]. Based on our previous work, we first synthesized a series of *para*-(**2a**–**i**) and *meta*-substituted menadione derivatives (**3a**–**h**) in good-to-satisfactory yields (61–83%; see Scheme 1, route A).

For the preparation of the benzamido-NQ derivatives, the procedure had to be slightly modified by adjusting the amounts of the reagents used to minimize the formation of disubstituted compounds. Consequently, the *para*-substituted compounds **5a**,**b** and the *meta*-derivatives **6a**,**b** could be obtained in moderate yields (53–59%) (Scheme 1, route B). The synthesized compounds, together with their synthetic pathways and the resulting yields, are presented in Table 1.

### 2.2. Biological Evaluation

Synthesized benzamido–menadione and naphthoquinone derivatives were tested in vitro for their antiprotozoal activity against *P. falciparum* (NF54) in the erythrocyte stage and *T. brucei rhodesiense* (STIB900) in the bloodstream form. The evaluation of the compounds’ potential for toxicity was conducted using rat L6 skeletal muscle cell lines, and their corresponding selectivity index (SI) was determined based on the ratio between the cytotoxic and antiplasmodial activity of each compound (SI = IC_50(L6)_/IC_50(parasite)_).

According to the recommended hit-to-lead identification criteria [23,24,25], almost all benzamido–menadiones exhibited high activity towards NF54 (IC_50_ < 1 µM, Table 2) and favourable selectivity, particularly **2c**, **2e**, **2f**, **2g**, and **2i** (SI = 2900–10,000). The strongest activity in the NF54 assay with excellent selectivity was exhibited by the 2-fluoro-5-trifluoromethylbenzamido derivative **2f** (IC_50_ = 0.021 μM, SI = 10,000). In this setting, only the *meta*-substituted benzamido–menadiones **3a**, **3d**, **3g**, and **3h** displayed moderate antiplasmodial effects (IC_50_ = 1–14 µM). The benzamido–naphthoquinones (**5a**, **5b**, **6a**, and **6b**) generally displayed lower antiplasmodial activity and selectivity. A certain trypanocidal effect was also observed (most notably with benzamido-NQ **5a**), although it was generally much less developed.

Toxicity prediction was performed using the online platform ProTox-3.0, a web-based tool that utilizes a comprehensive database and was developed to evaluate the toxicological properties of chemical compounds using advanced machine learning algorithms [26]. These predictions indicated low acute oral toxicity for our compounds, with LD_50_ values of no less than 1000 mg/kg. The only negative outlier was the trifluoromethoxy derivative **2h**, with a calculated oral toxicity of 57 mg/kg. Due to their presumed negligible oral toxicity [27], the development of drugs based on the benzamido–menadione scaffold could be a promising approach to retrieve new antiprotozoal agents.

### 2.3. Physicochemical Investigations

The hit-to-lead process (H2L) is an essential part of modern drug discovery and involves the optimization of validated hit structures into lead molecules [28,29,30]. While a lead molecule may not yet be perfect, it has structural properties as well as inherent efficacy and physicochemical characteristics that would be difficult to address in later phases of drug development. Calculated physicochemical properties, especially those falling under the rule of five (Ro5), have long played a central role in drug development, as they have been shown to be effective tools in predicting the absorption and permeation of compounds [31,32,33,34,35].

An important factor in selecting hits is to prioritize compounds with higher ligand efficiency. Of all the ligand efficiency metrics (LE, SILE, FQ, LLE, and LELP), the lipophilic ligand efficiency LLE (also known as LipE) is the most robust and widely applicable due to its correlation to enthalpy [36,37,38]. A selection of important ligand efficiency metrics and multi-parameter scores, along with their calculation method, is shown in Table 3.

The size-independent ligand efficiency (SILE) [39] and fit quality (FQ) [40] scores have been developed to identify drug candidates across a wide range of ligand sizes, thereby facilitating the discovery of ligands with good ligand efficiency or even ligands with exceptional efficiency.

However, it has been shown that setting ambitious scores for these metrics is unwise, as they are target-dependent [29]. The recommended strategy is to focus on maximizing the pivotal LLE and prioritizing series with higher average values. Due to their significance in the drug discovery process, we calculated the most important physicochemical parameters, ligand efficiency indices, and multi-parameter scores of our tested compounds and listed them in Tables S1 and S2 in the Supplementary Material.

### 2.4. Structure-Activity Relationships (SAR) of the Antiplasmodial Activity

The vast majority of the compounds examined in this study complied with the established recommendations of Ro5 [41], AB-MPS [42], and PFI [43]. However, some argue that focusing too much on these guidelines hinders the development of new drug leads [44]. Therefore, this study focuses on ligand efficiency metrics as the most important tool for lead optimization and controlling lipophilicity in relation to efficacy.

As in our previous work [18], we found that some of the calculated ligand efficiency metrics, especially size-normalized SILE and FQ, correlated highly with the observed antiplasmodial activity (e.g., SILE_P.f._, ρ = −0.99; FQ_P.f._, ρ = −0.99). Spearman’s rho (ρ) used for this purpose is robust against outliers and does not require normally distributed data, as extreme values are converted into ranks, thereby reducing their disproportionate influence on the calculation. The observed Spearman correlation for SILE and FQ confirms that hit compounds can be more accurately identified by adjusting their lipophilicity relative to their molecular size. It is noteworthy to mention that alternative size-independent ligand efficiency metrics, such as the ligand efficiency lipophilicity price (LELP) [45] and the Astex lipophilicity ligand efficiency (LLE_AT_) [46], exhibited no substantial correlation with the efficacy of our compounds.

**Figure 2:**
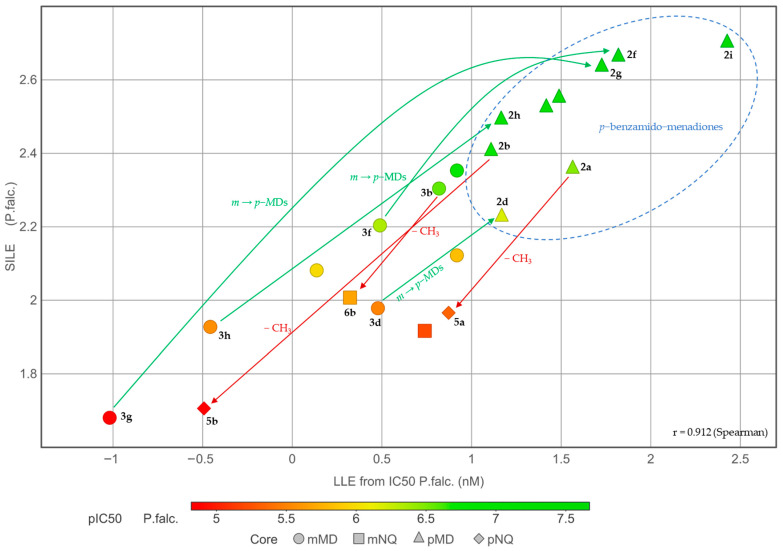
The scatterplot illustrates the relationship between lipophilic ligand efficiency (LLE) and size-independent ligand efficiency (SILE) of the synthesized compounds. This analysis also includes some optimization trajectories. The colours correspond to their respective pIC_50_ values, while the marker shapes correspond to the respective scaffolds.

Examination of the ligand efficiency metrics of our compounds in an LLE/SILE scatterplot revealed clear correlations between compounds with similar structural elements and corresponding antiplasmodial activities (Figure 2 and Figure S1 in the Supplementary Materials). Here, the activity of *para*-substituted benzamido–menadiones is particularly evident, as all these compounds are situated in the recommended upper-right quadrant (LLE > 1.1, SILE > 2.2) and demonstrate remarkable antiplasmodial activity (pIC_50_ ≥ 6.9). Among the *para*-MD derivatives, the multifluorinated derivative **2i** (pIC_50_ of 7.6) is particularly outstanding; only compounds **2a** and **2d** exhibited slightly lower values (with a pIC_50_ of 6.2 and 6.5, respectively). In contrast, the NQ derivatives synthesized in our study (**5a**, **5b**, **6a**, and **6b**) generally showed reduced activity (pIC_50_ ≤ 5.7) and were predominantly located in the lower-left region of the LLE/SILE scatterplot.

The trajectories of selected compounds in the LLE and SILE space are also shown in Figure 2, providing an excellent opportunity to evaluate the optimization process in drug development. For instance, an alteration from *meta*- to *para*-substituted derivatives (**3d** → **2d, 3f** → **2f**, **3g** → **2g**, **3h** → **2h**) has been observed to result in a substantial enhancement in effectiveness, as indicated by an explicit migration toward the north-eastern quadrant of the LLE-SILE plot. In contrast, the removal of the methyl group from the menadione backbone results in a substantial reduction in its antiplasmodial effect (**2a** → **5a**, **2b** → **5b, 3b** → **6b**).

Incidentally, this behaviour is not only limited to the LLE/SILE plane; the LLE/FQ alternative performed is almost identical to the LLE/SILE plot (see Figure S2 in the Supplementary Material), suggesting its potential application in a comparable manner. These results underscore the potential of the LLE/SILE, as well as the LLE/FQ plot, to serve as valuable tools regarding the effectiveness of different functional groups and moieties in the context of drug development.

**Figure 3:**
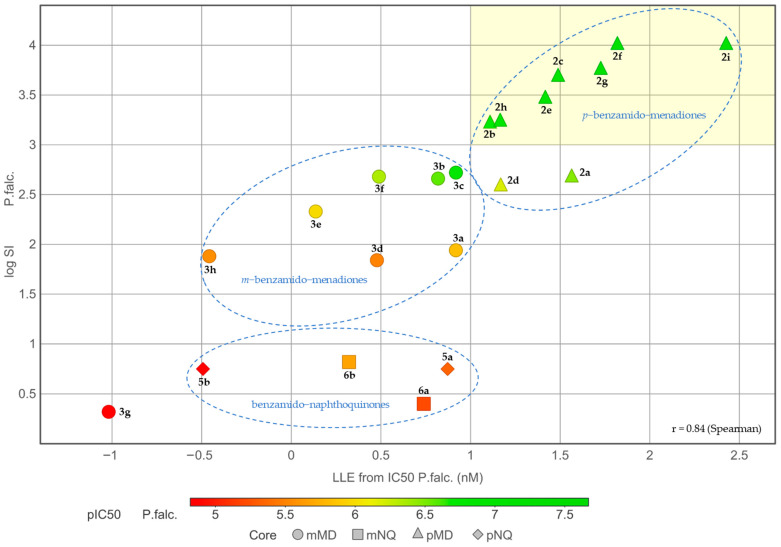
Plot of LLE (*P. falciparum*) data obtained in this study (x) vs. calculated log SI (*P. falciparum*) (y). The colours are assigned according to their respective pIC_50_ values, while the marker shapes are assigned to the respective scaffolds. Substances that lie in the yellow-shaded area combined high selectivity with acceptable ligand efficiency values.

In light of the prevailing consensus that LLE is the most robust and widely used metric for assessing quality in drug discovery [36], we also correlated this key parameter with the selectivity of the tested compounds as log SI (Figure 3).

The remarkable capability of *p*-substituted MDs is further evidenced by the LLE/log SI scatterplot, which exhibited a clear clustering of the evaluated compounds according to lipophilic efficiency and selectivity. With the exception of **2a** (log SI = 2.7) and **2d** (log SI = 2.6), the log SI values are > 3.2 in all cases, indicating that these compounds can be found in the desired upper-right section of the LLE/log SI chart. The *meta*-MD derivatives are situated in the central region of the scatterplot (with the exception of **3g**), while **3g** and all of the NQs exhibit less favourable prospects and are located in the southwestern part of the plane.

Abad-Zapatero et al. proposed an alternative approach to guide the transition from hit to lead, which involved the implementation of the binding efficacy index (BEI), an extension of the ligand efficiency (LE), and the surface-binding efficacy index (SEI) [47]. This concept was enhanced by the introduction of the Atlas-CBS system, which enables efficient lead optimization and prioritization by mapping the distribution of various compounds in an nBEI-NSEI plane [48,49,50]. This pair of variables is ideal for identifying the ‘direction’ of the optimization path provided by the corresponding scaffold, which corresponds to the slope given by NPOL (N + O count). It also allows for identifying likely drug candidates in the most probable ‘efficiency region’ situated in the north-eastern part of the plane. Furthermore, this approach facilitates the establishment of a direct graphical visualization of the guidelines proposed by Lipinski and colleagues, as outlined in Ro5 [50]. This provides further insight into the Ro5, in which the potency of the compounds is not considered.

**Figure 4:**
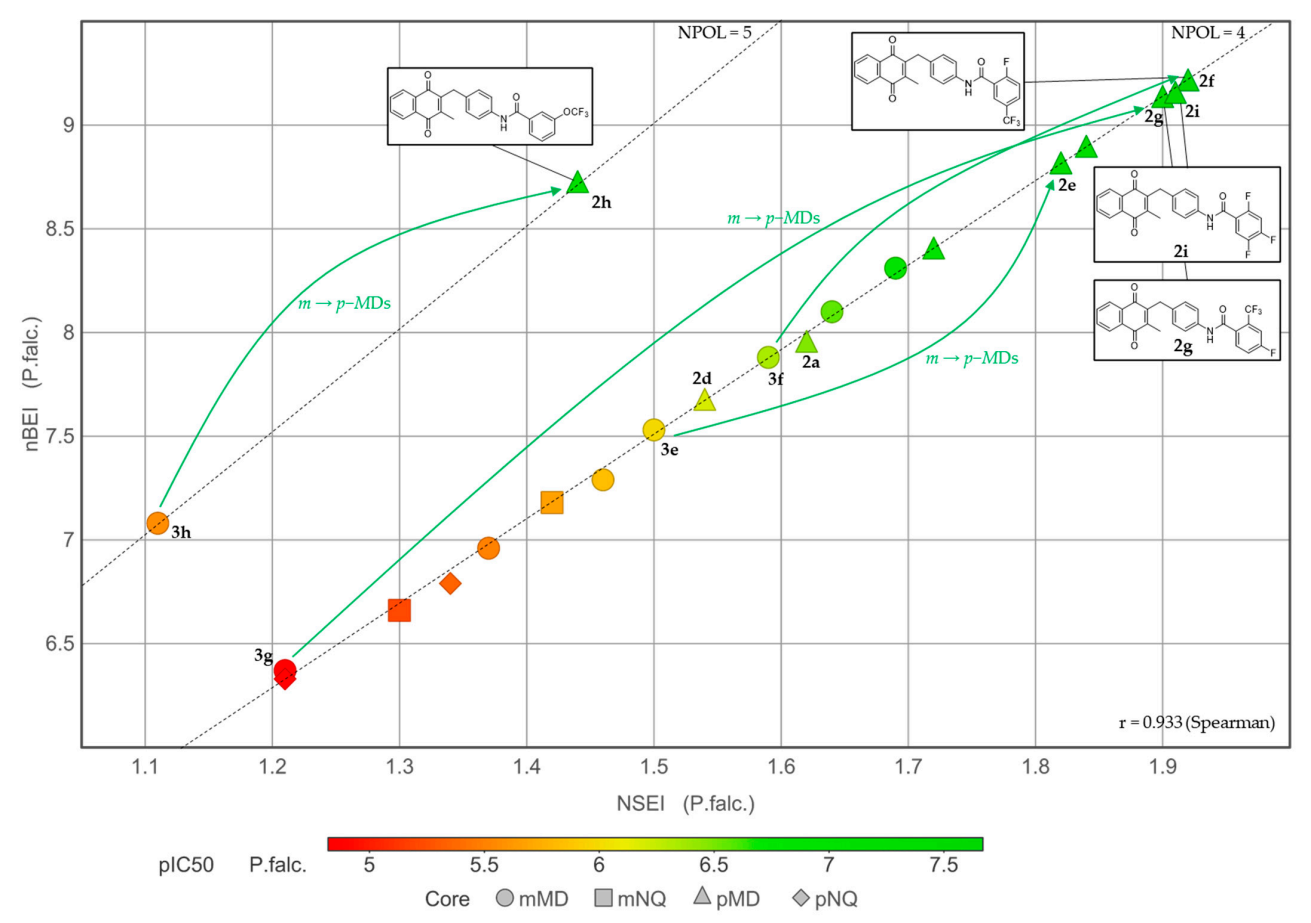
Application of the AtlasCBS concept, utilizing NSEI-nBEI plane analysis, to our synthesized compounds, encompassing the optimization of trajectories, with colours assigned according to the respective pIC_50_ values and marker shapes assigned to the respective scaffolds.

The application of the Atlas CBS system to our compounds corresponds remarkably well with the LLE/SILE plot described above. In this instance as well, the *para*-MD derivatives (again with the exception of **2a** and **2d**) have been identified as the most efficient compounds in terms of size and polarity and are located in the upper-right corner of the plane (Figure 4 and Figure S3 in the Supplementary Materials). Consequently, these compounds are regarded as promising candidates for further drug development.

The concept of trajectory mapping can also be used very effectively in the NSEI/nBEI scatterplot to monitor the optimization process in drug discovery. Again, the switch from *meta*- to *para*-substituted derivatives (**3e** → **2e, 3f** → **2f**, **3g** → **2g**, **3h** → **2h**) resulted in a substantial surge in activity, as demonstrated by a discernible shift toward the preferred north-east direction depicted in the NSEI/nBEI correlation plot. In contrast, the activity of all NQ derivatives was less promising, which is also clearly reflected in the NSEI/nBEI chart.

## 3. Materials and Methods

### 3.1. Chemistry

#### 3.1.1. General Information

All reagents and solvents were obtained from Merck and Fluorochem Ltd. Moisture-sensitive reactions were performed under an inert argon atmosphere. Each reaction was monitored by TLC on Merck TLC plates (silica gel 60 F254 0.2 mm, 200 × 200 mm) and detected at 254 nm. All reaction products were purified by flash column chromatography on silica gel 60 (Merck, Darmstadt, Germany, 70–230 mesh, pore diameter 60 Å) unless otherwise stated. Purity and homogeneity of the final compounds were checked by TLC and high-resolution mass spectrometry. Melting points were determined using a digital melting point apparatus (Electrothermal IA 9200, Thermo Fisher Scientific, Birmingham, UK).

The structures of all synthesized derivatives were determined by 1D and 2D NMR spectroscopy on a Bruker Avance Neo 400 MHz instrument (at 298 K) using 5 mm tubes. Chemical shifts were expressed in δ (ppm) using either tetramethylsilane (TMS) or the ^13^C signal of the solvents (CDCl_3_ δ 77.04 ppm, DMSO-*d*_6_ δ 39.45 ppm) as the internal standard. Accurate structural elucidation was confirmed by ^1^H, ^1^H- and ^1^H, ^13^C-correlation spectra (COSY, HSQC, HMBC, H2BC). ^1^H and ^13^C resonances were numbered according to the formulae (see Supplementary Material), and signals marked with an asterisk are interchangeable.

ESI and APCI mass spectra were acquired by analyzing sample solutions on an Ultimate 3000 HPLC with a Q Exactive™ Hybrid Quadrupole-Orbitrap™ mass spectrometer equipped with a heated ESI II source or an APCI source (Thermo Fisher Scientific) in positive or negative ionization mode.

#### 3.1.2. General Synthetic Procedure for the Preparation of Benzamido-Phenylacetic Acids

The aminophenylacetic acid (1 mmol) was dissolved in 2.0 M KOH (10 mL). Then, the benzoyl chloride derivative (1.1 equiv.) was added dropwise, and the solution was stirred at room temperature until TLC showed complete consumption of the starting material (18–24 h). After acidifying to pH 4.0 with 2.0 M aqueous HCl, the mixture was extracted three times with EtOAc (50 mL). The combined organic layers were washed with H_2_O, dried over Na_2_SO_4_, and concentrated in vacuo to yield the crude products as off-white solids, which were used without further purification.

#### 3.1.3. General Synthetic Procedure for Benzamido–Menadiones **2a**–**i** and **3a**–**h** (Route A)

Menadione (1.0 mmol) was added to a stirred solution of the respective benzamido-phenylacetic acid derivative (1.4 equiv.) in CH_3_CN (9 mL) and H_2_O (3 mL) and heated to 85 °C. AgNO_3_ (0.35 equiv.) was added first and then (NH_4_)_2_S_2_O_8_ (1.3 equiv.) dissolved in 4 mL CH_3_CN/H_2_O (3:1) dropwise over a period of 5 min. Stirring at 85 °C was continued until TLC showed complete consumption of the starting material (3–6 h). After cooling to ambient temperature, the mixture was extracted three times with CH_2_Cl_2_ (30 mL). The combined organic layers were washed with H_2_O, dried over Na_2_SO_4_, and concentrated in vacuo to give a residue, which was purified by flash chromatography as detailed below.

*N-{4-[(3-Methyl-1,4-dioxo-1,4-dihydronaphthalen-2-yl)methyl]phenyl}benzamide (***2a***).* Compound **2a** was obtained after stirring for 3 h, then purified by flash chromatography using cyclohexane:CH_2_Cl_2_:EtOAc (5:3:1). Yellow solid; yield: 79%; *R*_f_ = 0.53 (cyclohexane:CH_2_Cl_2_:EtOAc = 5:3:1); m.p.: 195–196 °C. ^1^H NMR (400 MHz, DMSO-d_6_): δ = 10.18 (s, 1H, NH), 8.01 (m, 2H, H-5, H-8), 7.92 (m, 2H, H-2″,H-6″), 7.85 (dd, *J* = 6.0, 3.4 Hz, 2H, H-6, H-7), 7.66 (d, *J* = 8.3 Hz, 2H, H-3′, H-5′), 7.57 (m, 1H, H-4″), 7.52 (d, *J* = 7.5 Hz, 2H, H-3″, H-5″), 7.21 (d, *J* = 8.3 Hz, 2H, H-2′, H-6′), 3.96 (s, 2H, H-9), 2.17 (s, 3H, CH_3_) ppm; ^13^C NMR (100 MHz, DMSO-d_6_): δ = 184.7 (C-4), 184.1 (C-1), 165.3 (CO(NH)), 144.5 * (C-2), 144.1 * (C-3), 137.3 (C-4′), 134.9 (C-1″), 133.9 (C-7), 133.8 (C-6), 133.3 (C-1′), 131.7 (C-4a), 131.4 (C-8a, C-4″), 128.4 (C-2′, C-6′), 128.3 (C-3″, C-5″), 127.6 (C-2″, C-6″), 125.8 (C-5, C-8), 120.5 (C-3′, C-5′), 31.1 (C-9), 12.9 (CH_3_) ppm; HRMS (ESI) calcd. for C_25_H_20_NO_3_ [M + H]^+^ = 382.1438; found: 382.1433.

*N-{4-[(3-Methyl-1,4-dioxo-1,4-dihydronaphthalen-2-yl)methyl]phenyl}-4-(trifluoromethyl)benzamide (***2b***).* Compound **2b** was obtained after stirring for 4 h, then purified by flash chromatography using cyclohexane:CH_2_Cl_2_:EtOAc (5:3:1). Yellow solid; yield: 72%; *R*_f_ = 0.47 (cyclohexane:CH_2_Cl_2_:EtOAc = 5:3:1); m.p.: 210–211 °C. ^1^H NMR (400 MHz, DMSO-*d*_6_): δ = 10.40 (s, 1H, NH), 8.11 (d, *J* = 8.1 Hz, 2H, H-2″, H-6″), 8.01 (m, 2H, H-5, H-8), 7.89 (d, *J* = 8.1 Hz, 2H, H-3″, H-5″), 7.84 (m, 2H, H-6, H-7), 7.66 (d, *J* = 8.4 Hz, 2H, H-3′, H-5′), 7.22 (d*, J* = 8.4 Hz, 2H, H-2′, H-6′), 3.97 (s, 2H, H-9), 2.17 (s, 3H, CH_3_) ppm; ^13^C NMR (100 MHz, DMSO-*d*_6_): δ = 184.7 (C-4), 184.1 (C-1), 164.1 (CO(NH)), 144.4 * (C-2), 144.2 * (C-3), 138.7 (C-1″), 136.9 (C-4′), 133.9 * (C-6), 133.8 * (C-7), 133.7 (C-1′), 131.6 * (C-8a), 131.4 * (C-4a), 131.1 (q, ^2^*J*_C,F_ = 32.0 Hz, C-4″), 128.5 (C-2′, C-6′, C-2″, C-6″), 125.9 * (C-5), 125.8 * (C-8), 125.3 (q, ^3^*J*_C,F_ = 3.7 Hz, C-3″, C-5″), 123.8 (q, ^1^*J*_C,F_ = 272.5 Hz, CF_3_), 120.6 (C3′, C-5′), 31.1 (C-9), 12.9 (CH_3_) ppm; HRMS (ESI) calcd. for C_26_H_17_F_3_NO_3_ [M − H]^–^ = 448.1161; found: 448.1173.

*2-Fluoro-N-{4-[(3-methyl-1,4-dioxo-1,4-dihydronaphthalen-2-yl)methyl]phenyl}-4-(trifluoromethyl)benzamide (***2c***).* Compound **2c** was obtained after stirring for 4.5 h, then purified by flash chromatography using cyclohexane:CH_2_Cl_2_:EtOAc (5:3:1). Yellow solid; yield: 81%; *R*_f_ = 0.43 (cyclohexane:CH_2_Cl_2_:EtOAc = 5:3:1); m.p.: 181–182 °C. ^1^H NMR (400 MHz, DMSO-*d*_6_): δ = 10.54 (s, 1H, NH), 8.01 (m, 2H, H-5, H-8), 7.85 (m, 2H, H-3″, H-6″), 7.84 (m, 2H, H-6, H-7), 7.71 (d, *J* = 8.1 Hz, 1H, H-5″), 7.60 (d, *J* = 8.3 Hz, 2H, H-3′, H-5′), 7.23 (d*, J* = 8.3 Hz, 2H, H-2′, H-6′), 3.97 (s, 2H, H-9), 2.16 (s, 3H, CH_3_) ppm; ^13^C NMR (100 MHz, DMSO-*d*_6_): δ = 184.6 (C-4), 184.1 (C-1), 161.3 (CO(NH)), 158.5 (d, ^1^*J*_C,F_ = 251.1 Hz, C-2″), 144.4 * (C-2), 144.2 * (C-3), 136.6 (C-4′), 133.9 (C-1′), 133.9 * (C-6), 133.8 * (C-7), 132.1 (qd, ^2^*J*_C,F_ = 33.0 Hz, ^3^*J*_C,F_ = 8.3 Hz, C-4″), 131.6 * (C-4a), 131.4 * (C-8a), 131.0 (d, ^3^*J*_C,F_ = 3.7 Hz, C-6″), 128.9 (d, ^2^*J*_C,F_ = 16.6 Hz, C-1″), 128.7 (C-2′, C-6′), 125.9 * (C-5), 125.8 * (C-8), 123.0 (dd, ^1^*J*_C,F_ = 272.8 Hz, ^4^*J*_C,F_ = 2.5 Hz, CF_3_), 121.5 (quint, ^4^*J*_C,F_ = 3.7 Hz, C-5″), 119.9 (C-3′, C-5′), 113.8 (dq, ^2^*J*_C,F_ = 25.6 Hz, ^3^*J*_C,F_ = 3.8 Hz, C-3″), 31.1 (C-9), 12.9 (CH_3_) ppm; HRMS (ESI) calcd. for C_26_H_16_F_4_NO_3_ [M − H]^–^ = 466.1066; found: 466.1073.

*4-Fluoro-N-{4-[(3-methyl-1,4-dioxo-1,4-dihydronaphthalen-2-yl)methyl]phenyl}benzamide (***2d***).* Compound **2d** was obtained after stirring for 4 h, then purified by flash chromatography using cyclohexane:CH_2_Cl_2_:EtOAc (5:3:1). Yellow solid; yield: 76%; *R*_f_ = 0.49 (cyclohexane:CH_2_Cl_2_:EtOAc = 5:3:1); m.p.: 198–200 °C. ^1^H NMR (400 MHz, DMSO-*d*_6_): δ = 10.20 (s, 1H, NH), 8.01 (m, 4H, H-5, H-8, H-2″, H-6″), 7.85 (m, 2H, H-6, H-7), 7.65 (d, *J* = 8.5 Hz, 2H, H-3′, H-5′), 7.34 (m, 2H, H-3″, H-5″), 7.21 (d, *J* = 8.5 Hz, 2H, H-2′, H-6′), 3.97 (s, 2H, H-9), 2.17 (s, 3H, CH_3_) ppm; ^13^C NMR (100 MHz, DMSO-*d*_6_): δ = 184.7 (C-4), 184.1 (C-1), 164.8 (d, ^1^*J*_C,F_ = 250.4 Hz, C-4″), 164.2 (CO(NH)), 144.5 * (C-2), 144.2 * (C-3), 137.2 (C-4′), 133.8 (C-6, C-7), 133.4 (C-1′), 132.0 (d, ^3^*J*_C,F_ = 9.7 Hz, C-2″, C-6″), 131.7 * (C-4a), 131.4 * (C-8a), 128.4 (C-2′, C-6′), 127.3 (d, ^4^*J*_C,F_ = 2.9 Hz, C-1″), 125.9 * (C-5), 125.8 * (C-8), 120.6 (C-3′, C-5′), 115.5 (d, ^2^*J*_C,F_ = 21.9 Hz, C-3″, C-5″), 31.1 (C-9), 12.9 (CH_3_) ppm; HRMS (ESI) calcd. for C_25_H_19_FNO_3_ [M + H]^+^ = 400.1349; found: 400.1339.

*4-Fluoro-N-{4-[(3-methyl-1,4-dioxo-1,4-dihydronaphthalen-2-yl)methyl]phenyl}-3-(trifluoromethyl)benzamide (***2e***).* Compound **2e** was obtained after stirring for 4.5 h, then purified by flash chromatography using cyclohexane:CH_2_Cl_2_:EtOAc (5:3:1). Yellow solid; yield: 65%; *R*_f_ = 0.40 (cyclohexane:CH_2_Cl_2_:EtOAc = 5:3:1); m.p.: 170–171 °C. ^1^H NMR (400 MHz, DMSO-*d*_6_): δ = 10.40 (s, 1H, NH), 8.31 (m, 2H, H-2″, H-6″), 8.00 (m, 2H, H-5, H-8), 7.83 (m, 2H, H-6, H-7), 7.68 (t, *J* = 9.9 Hz, 1H, H-5″), 7.64 (d, *J* = 8.5 Hz, 2H, H-3′, H-5′), 7.23 (d, *J* = 8.5 Hz, 2H, H-2′, H-6′), 3.96 (s, 2H, H-9), 2.16 (s, 3H, CH_3_) ppm; ^13^C NMR (100 MHz, DMSO-*d*_6_): δ = 184.6 (C-4), 184.1 (C-1), 162.8 (CO(NH)), 160.5 (dq, ^1^*J*_C,F_ = 258.5, ^3^*J*_C,F_ = 2.2 Hz, C-4″), 144.4 * (C-2), 144.2 * (C-3), 136.8 (C-4′), 134.9 (d, ^3^*J*_C,F_ = 9.9 Hz, C-6″), 133.8 (C-6, C-7, C-1′), 131.6 (d, ^4^*J*_C,F_ = 3.5 Hz, C-1″), 131.6 * (C-4a), 131.4 * (C-8a), 128.5 (C-2′, C-6′), 126.8 (qd, ^3^*J*_C,F_ = 4.5, 2.0 Hz, C-2″), 125.9 * (C-5), 125.8 * (C-8), 122.3 (qd, ^1^*J*_C,F_ = 272.5, ^3^*J*_C,F_ = 1.1 Hz, CF_3_), 120.7 (C-3′, C-5′), 117.4 (d, ^2^*J*_C,F_ = 21.0 Hz, C-5″), 116.5 (qd, ^2^*J*_C,F_ = 32.7, 12.7 Hz, C-3″), 31.1 (C-9), 12.9 (CH_3_) ppm; HRMS (ESI) calcd. for C_26_H_16_F_4_NO_3_ [M − H]^–^ = 466.1066; found: 466.1073.

*2-Fluoro-N-{4-[(3-methyl-1,4-dioxo-1,4-dihydronaphthalen-2-yl)methyl]phenyl}-5-(trifluoromethyl)benzamide (***2f***).* Compound **2f** was obtained after stirring for 5 h, then purified by flash chromatography using cyclohexane:CH_2_Cl_2_:EtOAc (5:3:1). Yellow solid; yield: 62%; *R*_f_ = 0.42 (cyclohexane:CH_2_Cl_2_:EtOAc = 5:3:1); m.p.: 179–180 °C. ^1^H NMR (400 MHz, DMSO-*d*_6_): δ = 10.53 (s, 1H, NH), 8.01 (m, 3H, H-5, H-8, H-6″), 7.96 (m, 1H, H-4″), 7.84 (m, 2H, H-6, H-7), 7.60 (d*, J* = 8.6 Hz, 2H, H-3′, H-5′), 7.59 (t, *J* = 9.1 Hz, 1H, H-3″), 7.23 (d, *J* = 8.6 Hz, 2H, H-2′, H-6′), 3.97 (s, 2H, H-9), 2.16 (s, 3H, CH_3_) ppm; ^13^C NMR (100 MHz, DMSO-*d*_6_): δ = 184.6 (C-4), 184.1 (C-1), 161.0 (CO(NH)), 160.8 (dq, ^1^*J*_C,F_ = 255.9, ^5^*J*_C,F_ = 1.2 Hz, C-2″), 144.4 * (C-2), 144.2 * (C-3), 136.6 (C-4′), 133.9 (C-1′), 133.9 * (C-6), 133.8 * (C-7), 131.6 * (C-4a), 133.4 * (C-8a), 129.6 (C-4″), 128.7 (C-2′, C-6′), 127.3 (p, ^3^*J*_C,F_ = 3.9 Hz, C-6″), 125.9 (d, ^2^*J*_C,F_ = 17.0 Hz, C-1″), 125.9 * (C-5), 125.8 * (C-8), 125.3 (qd, ^2^*J*_C,F_ = 32.8, ^4^*J*_C,F_ = 3.4 Hz, C-5″), 123.5 (q, ^1^*J*_C,F_ = 272.1 Hz, CF_3_), 120.0 (C-3′, C-5′), 117.6 (d, ^2^*J*_C,F_ = 23.6 Hz, C-3″), 31.1 (C-9), 12.9 (CH_3_) ppm; HRMS (APCI) calcd. for C_26_H_18_F_4_NO_3_ [M + H]^+^ = 468.1217; found: 468.1201.

*4-Fluoro-N-{4-[(3-methyl-1,4-dioxo-1,4-dihydronaphthalen-2-yl)methyl]phenyl}-2-(trifluoromethyl)benzamide (***2g***).* Compound **2g** was obtained after stirring for 4 h, then purified by flash chromatography using cyclohexane:CH_2_Cl_2_:EtOAc (5:3:1). Yellow solid; yield: 81%; *R*_f_ = 0.43 (cyclohexane:CH_2_Cl_2_:EtOAc = 5:3:1); m.p.: 181–182 °C. ^1^H NMR (400 MHz, DMSO-*d*_6_): δ = 10.50 (s, 1H, NH), 8.01 (m, 2H, H-5, H-8), 7.84 (m, 2H, H-6, H-7), 7.76 (m, 2H, H-3″, H-6″), 7.66 (td, *J* = 8.5, 2.6 Hz, 1H, H-5″), 7.56 (d, *J* = 8.3 Hz, 2H, H-3′, H-5′), 7.21 (d, *J* = 8.3 Hz, 2H, H-2′, H-6′), 3.96 (s, 2H, H-9), 2.17 (s, 3H, CH_3_) ppm; ^13^C NMR (100 MHz, DMSO-*d*_6_): δ = 184.7 (C-4), 184.1 (C-1), 164.4 (CO(NH), 161.8 (d, ^1^*J*_C,F_ = 248.5 Hz, C-4″), 144.4 * (C-2); 144,2 * (C-3), 136.9 (C-4′), 133.9 * (C-6), 133.8 * (C-7), 133.7 (C-1′), 132.8 (C-1″), 131.6 * (C-4a), 131.4 * (C-8a), 131.3 (d, ^3^*J*_C,F_ = 8.6 Hz, C-6″), 128.6 (C-2′, C-6′), 128.1 (qd, ^2^*J*_C,F_ = 32.6, ^3^*J*_C,F_ = 8.1 Hz, C-2″), 125.9 * (C-5), 125.8 * (C-8), 122.8 (qd, ^1^*J*_C,F_ = 274.0, ^4^*J*_C,F_ = 2.6 Hz, CF_3_), 119.8 (C-3′, C-5′), 119.4 (d, ^2^*J*_C,F_ = 21.2 Hz, C-5″), 114.0 (dq, ^2^*J*_C,F_ = 25.5, ^3^*J*_C,F_ = 4.8 Hz, C-3″), 31.1 (C-9), 12.9 (CH_3_) ppm; HRMS (ESI) calcd. for C_26_H_16_F_4_NO_3_ [M − H]^−^ = 466.1066; found: 466.1073.

*N-{4-[(3-Methyl-1,4-dioxo-1,4-dihydronaphthalen-2-yl)methyl]phenyl}-3-(trifluoromethoxy)benzamide (***2h***).* Compound **2h** was obtained after stirring for 4 h, then purified by flash chromatography using cyclohexane:CH_2_Cl_2_:EtOAc (5:3:1). Yellow solid; yield: 75%; *R*_f_ = 0.50 (cyclohexane:CH_2_Cl_2_:EtOAc = 5:3:1); m.p.: 115–116 °C. ^1^H NMR (400 MHz, DMSO-*d*_6_): δ = 10.32 (s, 1H, NH), 8.01 (m, 2H, H-5, H-8), 7.97 (m, 1H, H-6″), 7.87 (s, 1H, H-2″), 7.83 (m, 2H, H-6, H-7), 7.66 (m, 3H, H-3′, H-5′, H-5″), 7.58 (m, 1H, H-4″), 7.22 (d, *J* = 8.3 Hz, 2H, H-2′, H-6′), 3.96 (s, 2H, H-9), 2.16 (s, 3H, CH_3_) ppm; ^13^C NMR (100 MHz, DMSO-*d*_6_): δ = 184.6 (C-4), 184.1 (C-1), 163.6 (CO(NH)), 148.3 (q, *^3^J*_C,F_ = 1.9 Hz, C-3″), 144.4 * (C-2), 144.2 * (C-3), 136.9 (C-4′), 133.8 (C-6, C-7), 133.7 (C-1`), 133.0 (C-1″), 130.9 (C-5″), 131.6 * (C-4a), 131.4 * (C-8a), 128.5 (C-2′, C-6′), 126.7 (C-6″), 125.9 * (C-5), 125.8 * (C-8), 123.9 (C-4″), 120.7 (C-3′, C-5′), 120.1 (C-2″), 120.0 (q, ^1^*J*_C,F_ = 256.8 Hz, OCF_3_), 31.1 (C-9), 12.9 (CH_3_) ppm HRMS (ESI) calcd. for C_26_H_17_F_3_NO_4_ [M − H]^−^ = 464.1110; found: 464.1117.

*2,4,5-Trifluoro-N-{4-[(3-methyl-1,4-dioxo-1,4-dihydronaphthalen-2-yl)methyl]phenyl}benzamide (***2i***).* Compound **2i** was obtained after stirring for 5 h, then purified by flash chromatography using cyclohexane:CH_2_Cl_2_:EtOAc (5:3:1). Yellow solid; yield: 61%; *R*_f_ = 0.45 (cyclohexane:CH_2_Cl_2_:EtOAc = 5:3:1); m.p.: 175–177 °C. ^1^H NMR (400 MHz, DMSO-*d*_6_): δ = 10.41 (s, 1H, NH), 8.00 (m, 2H, H-5, H-8), 7.84 (m, 2H, H-6, H-7), 7.81 (m, 1H, H-6″), 7.72 (td, *J* = 10.3, 6.4 Hz, 1H, H-3″), 7.58 (d, *J* = 8.6 Hz, 2H, H-3′, H-5′), 7.21 (d, *J* = 8.6 Hz, 2H, H-2′, H-6′), 3.96 (s, 2H, H-9), 2.15 (s, 3H, CH_3_) ppm; ^13^C NMR (100 MHz, DMSO-*d*_6_): δ = 184.6 (C-4), 184.1 (C-1), 160.4 (CO(NH)), 154.6 (ddd, ^1^*J*_C,F_ = 248.4, ^3^*J*_C,F_ = 10.8, ^4^*J*_C,F_ = 2.4 Hz, C-2″), 150.4 (ddd, ^1^*J*_C,F_ = 252.4, ^2^*J*_C,F_ = 14.1, ^3^*J*_C,F_ = 13.0 Hz, C-4″), 145.8 (ddd, ^1^*J*_C,F_ = 243.9, ^2^*J*_C,F_ = 12.7, ^4^*J*_C,F_ = 3.6 Hz, C-5″), 144.4 * (C-2), 144.2 * (C-3), 136.7 (C-4′), 133.9 * (C-6), 133.8 *(C-7), 133.8 (C-1′), 131.6 * (C-4a), 131.4 * (C-8a), 128.6 (C-2′, C-6′), 125.9 * (C-5), 125.8 * (C-8), 121.6 (ddd, ^2^*J*_C,F_ = 17.5, ^3^*J*_C,F_ = 5.0, ^4^*J*_C,F_ = 4.2 Hz, C-1″), 120.0 (C-3′, C-5′), 118.0 (ddd, ^2^*J*_C,F_ = 20.6, ^3^*J*_C,F_ = 4.5, 1.5 Hz, C-6″), 106.8 (dd, ^2^*J*_C,F_ = 29.1, 21.5 Hz, C-3″), 31.1 (C-9), 12.9 (CH_3_) ppm; HRMS (APCI) calcd. for C_25_H_17_F_3_NO_3_ [M + H]^+^ = 436.1155; found: 436.1136.

*N-{3-[(3-Methyl-1,4-dioxo-1,4-dihydronaphthalen-2-yl)methyl]phenyl}benzamide (***3a***).* Compound **3a** was obtained after stirring for 4 h, then purified by flash chromatography using cyclohexane:CH_2_Cl_2_:EtOAc (5:3:1). Yellow solid; yield: 72%; *R*_f_ = 0.53 (cyclohexane:CH_2_Cl_2_:EtOAc = 5:3:1); m.p.: 190–191 °C. ^1^H NMR (400 MHz, DMSO-*d*_6_): δ = 10.15 (s, 1H, NH), 8.03 (m, 2H, H-5, H-8), 7.90 (m, 2H, H-2″, H-6″), 7.86 (m, 2H, H-6, H-7), 7.69 (dt*, J* = 8.0, 1.6 Hz, 1H, H-4′), 7.56 (m, 1H, H-4″), 7.55 (m, 1H, H-2′), 7.50 (m, 2H, H-3″, H-5″), 7.26 (t, *J* = 7.7 Hz, 1H, H-5′), 7.01 (ddd, *J* = 7.7, 1.9, 1.0 Hz, 1H, H-6′), 4.00 (s, 2H, H-9), 2.19 (s, 3H, CH_3_) ppm; ^13^C NMR (100 MHz, DMSO-*d*_6_): δ = 184.7 (C-4), 184.0 (C-1), 165.5 (CO(NH)), 144.4 * (C-2), 144.3 * (C-3), 139.3 (C-3′), 138.6 (C-1′), 134.9 (C-1″), 134.0 (C-6, C-7), 131.6 * (C-4a), 131.4 *(C-8a), 131.4 (C-4″), 128.7 (C-5′), 128.3 (C-3″, C-5″), 127.6 (C-2″, C-6″), 126.0 * (C-5), 125,9 * (C-8), 123.8 (C-6′), 119.8 (C-2′), 118.1 (C-4′), 31.7 (C-9), 13.0 (CH_3_) ppm; HRMS (ESI) calcd. for C_25_H_20_NO_3_ [M + H]^+^ = 382.1443; found: 382.1432.

*N-{3-[(3-Methyl-1,4-dioxo-1,4-dihydronaphthalen-2-yl)methyl]phenyl}-4-(trifluoromethyl)benzamide (***3b***).* Compound **3b** was obtained after stirring for 6 h, then purified by flash chromatography using cyclohexane:CH_2_Cl_2_:EtOAc (5:3:1). Yellow solid; yield: 72%; *R*_f_ = 0.47 (cyclohexane:CH_2_Cl_2_:EtOAc = 5:3:1); m.p.: 207–208 °C. ^1^H NMR (400 MHz, DMSO-*d*_6_): δ = 10.36 (s, 1H, NH), 8.09 (d, *J* = 8.1 Hz, 2H, H-2″, H-6″), 8.02 (m, 2H, H-5, H-8), 7.87 (d*, J* = 8.5 Hz, 2H, H-3″, H-5″), 7.85 (m, 2H, H-6, H-7), 7.70 (d, *J* = 7.9 Hz, 1H, H-4′), 7.54 (s, 1H, H-2′), 7.28 (t, *J* = 7.9 Hz, 1H, H-5′), 7.04 (d*, J* = 7.9 Hz, 1H, H-6′), 4.01 (s, 2H, H-9), 2.19 (s, 3H, CH_3_) ppm; ^13^C NMR (100 MHz, DMSO-*d*_6_): δ = 184.6 (C-4), 183.9 (C-1), 164.3 (CO(NH)), 144.4 (C-2, C-3), 139.0 (C-3′), 138.7 (q, ^5^*J*_C,F_ = 1.3 Hz, C-1″), 133.9 (C-6, C-7), 131.6 * (C-4a), 131.4 * (C-8a), 131.2 (q, ^2^*J*_C,F_ = 32.0 Hz, C-4″), 128.7 (C-5′), 128.5 (C-2″, C-6″), 126.0 * (C-5), 125.9 * (C-8), 125.3 (q, ^3^*J*_C,F_ = 3.9 Hz, C-3″, C-5″), 124.2 (C-6′), 123.8 (q, ^1^*J*_C,F_ = 272.4 Hz, CF_3_), 119.8 (C-2′), 118.2 (C-4′), 31.7 (C-9), 13.0 (CH_3_) ppm; HRMS (ESI) calcd. for C_26_H_17_F_3_NO_3_ [M − H]^−^ = 448.1161; found: 448.1168.

*2-Fluoro-N-{3-[(3-methyl-1,4-dioxo-1,4-dihydronaphthalen-2-yl)methyl]phenyl}-4-(trifluoromethyl)benzamide (***3c***).* Compound **3c** was obtained after stirring for 6 h, then purified by flash chromatography using cyclohexane:CH_2_Cl_2_:EtOAc (5:3:1). Yellow solid; yield: 69%; *R*_f_ = 0.49 (cyclohexane:CH_2_Cl_2_:EtOAc = 5:3:1); m.p.: 177–178 °C. ^1^H NMR (400 MHz, DMSO-*d*_6_): δ = 10.51 (s, 1H, NH), 8.02 (m, 2H, H-5, H-8), 7.85 (m, 4H, H-6, H-7, H-3″, H-6″), 7.70 (d, *J* = 8.0 Hz, 1H, H-5″), 7.63 (d, *J* = 7.9 Hz, 1H, H-4′), 7.46 (s br, 1H, H-2′), 7.28 (t, *J* = 7.9 Hz, 1H, H-5′), 7.04 (d, *J* = 7.9 Hz, 1H, H-6′), 4.00 (s, 2H, H-9), 2.18 (s, 3H, CH_3_) ppm; ^13^C NMR (100 MHz, DMSO-*d*_6_): δ = 184.6 (C-4), 183.9 (C-1), 161.4 (CO(NH)), 158.5 (d, ^1^*J*_C,F_ = 251.1 Hz, C-2″), 144.4 * (C-2), 144.3 * (C-3), 138.9 (C-1′), 138.7 (C-3′), 134.0 * (C-6), 133.9 * (C-7), 132.1 (qd, ^2^*J*_C,F_ = 33.0, ^3^*J*_C,F_ = 8.1 Hz, C-4″), 131.6 * (C-4a), 131.4 * (C-8a), 131.0 (d, ^3^*J*_C,F_ = 3.4 Hz, C-6″), 128.9 (C-5′), 128.9 (d, ^2^*J*_C,F_ = 15.5 Hz, C-1″), 126.0 * (C-5), 125.9 * (C-8), 124.3 (C-6′), 123.0 (qd, ^1^*J*_C,F_ = 272.8 Hz, CF_3_), 121.4 (C-5″), 119.1 (C-2′), 117.5 (C-4′), 113.7 (dq, ^2^*J*_C,F_ = 25.7 Hz, ^3^*J*_C,F_ = 3.9 Hz, C-3″), 31.6 (C-9), 13.0 (CH_3_) ppm; HRMS (ESI) calcd. for C_26_H_16_F_4_NO_3_ [M − H]^−^ = 466.1066; found: 466.1072.

*4-Fluoro-N-{3-[(3-methyl-1,4-dioxo-1,4-dihydronaphthalen-2-yl)methyl]phenyl}benzamide (***3d***).* Compound **3d** was obtained after stirring for 4 h, then purified by flash chromatography using cyclohexane:CH_2_Cl_2_:EtOAc (5:3:1). Yellow solid; yield: 71%; *R*_f_ = 0.49 (cyclohexane:CH_2_Cl_2_:EtOAc = 5:3:1); m.p.: 199–200 °C. ^1^H NMR (400 MHz, DMSO-*d*_6_): δ = 10.16 (s, 1H, NH), 8.02 (m, 2H, H-5, H-8), 7.97 (m, 2H, H-2″, H-6″), 7.85 (m, 2H, H-6, H-7), 7.67 (dt, *J* = 7.9, 1.9 Hz, 1H, H-4′), 7.53 (t, *J* = 1.9 Hz, 1H, H-2′), 7.33 (m, 2H, H-3″, H-5″), 7.25 (t, *J* = 7.9 Hz, 1H, H-5′), 7.00 (dt, *J* = 7.9, 1.9 Hz, 1H, H-6′), 3.99 (s, 2H, H-9), 2.18 (s, 3H, CH_3_) ppm; ^13^C NMR (100 MHz, DMSO-*d*_6_): δ = 184.6 (C-4), 183.9 (C-1), 164.4 (CO(NH)), 163.9 (d, ^1^*J*_C,F_ = 249.1 Hz, C-4″), 144.4 * (C-2), 144.3 * (C-3), 139.2 (C-3′), 138.6 (C-1′), 133.9 (C-6, C-7), 131.6 * (C-4a), 131.4 * (C-8a), 131.3 (d, ^4^*J*_C,F_ = 2.9 Hz, C-1″), 130.3 (d, ^3^*J*_C,F_ = 9.0 Hz, C-2″, C-6″), 128.7 (C-5′), 126.0 * (C-5), 125.9 * (C-8), 123.9 (C-6′), 119.8 (C-2′), 118.2 (C-4′), 115.2 (d, ^2^*J*_C,F_ = 21.8 Hz, C-3″, C-5″), 31.7 (C-9), 13.0 (CH_3_) ppm; HRMS (ESI) calcd. for C_25_H_17_FNO_3_ [M − H]^−^ = 398.1192; found: 398.1199.

*4-Fluoro-N-{3-[(3-methyl-1,4-dioxo-1,4-dihydronaphthalen-2-yl)methyl]phenyl}-3-(trifluoromethyl)benzamide (***3e***).* Compound **3e** was obtained after stirring for 4.5 h, then purified by flash chromatography using cyclohexane:CH_2_Cl_2_:EtOAc (5:3:1). Yellow solid; yield: 83%; *R*_f_ = 0.37 (cyclohexane:CH_2_Cl_2_:EtOAc = 5:3:1); m.p.: 168–169 °C. ^1^H NMR (400 MHz, DMSO-*d*_6_): δ = 10.38 (s, 1H, NH), 8.29 (m, 2H, H-2″, H-6″), 8.02 (m, 2H, H-5, H-8), 7.85 (m, 2H, H-6, H-7), 7.65 (m, 2H, H-4′, H-5″), 7.53 (t, *J* = 1.9 Hz, 1H, H-2′), 7.28 (t, *J* = 7.9 Hz, 1H, H-5′), 7.03 (m, 1H, H-6′), 4.01 (s, 2H, H-9), 2.19 (s, 3H, CH_3_) ppm; ^13^C NMR (100 MHz, DMSO-*d*_6_): δ = 184.6 (C-4), 184.0 (C-1), 163.0 (CO(NH)), 160.5 (dq, ^1^*J*_C,F_ = 258.2, ^3^*J*_C,F_ = 2.4 Hz, C-4″), 144.4 (C-2, C-3), 138.9 (C-3′), 138.7 (C-1′), 135.0 (d, ^3^*J*_C,F_ = 9.7 Hz, C-6″), 134.0 * (C-6), 133.9 * (C-7), 131.7 (d, ^4^*J*_C,F_ = 3.5 Hz, C-1″), 131.6 * (C-4a), 131.4 * (C-8a), 128.7 (C-5′), 126.9 (qd, ^3^*J*_C,F_ = 4.6, 2.0 Hz, C-2″), 126.0 * (C-5), 125.9 * (C-8), 124.2 (C-6′), 122.3 (dq, ^1^*J*_C,F_ = 272.5, ^3^*J*_C,F_ = 1.1 Hz, CF_3_), 120.0 (C-2′), 118.4 (C-4′), 117.4 (d, ^2^*J*_C,F_ = 20.8 Hz, C-5″), 116,4 (qd, ^2^*J*_C,F_ = 32.7, 12.7 Hz, C-3″), 31.7 (C-9), 13.0 (CH_3_) ppm; HRMS (ESI) calcd. for C_26_H_16_F_4_NO_3_ [M − H]^−^ = 466.1066; found: 466.1075.

*2-Fluoro-N-{3-[(3-methyl-1,4-dioxo-1,4-dihydronaphthalen-2-yl)methyl]phenyl}-5-(trifluoromethyl)benzamide (***3f***).* Compound **3f** was obtained after stirring for 5 h, then purified by flash chromatography using cyclohexane:CH_2_Cl_2_:EtOAc (5:3:1). Yellow solid; yield: 62%; *R*_f_ = 0.48 (cyclohexane:CH_2_Cl_2_:EtOAc = 5:3:1); m.p.: 173–175 °C. ^1^H NMR (400 MHz, DMSO-*d*_6_): δ = 10.50 (s, 1H, NH), 8.02 (m, 3H, H-5, H-6″, H-8), 7.96 (m, 1H, H-4″), 7.84 (m, 2H, H-6, H-7), 7.62 (m, 1H, H-4′), 7.58 (t, *J* = 9.1 Hz, 1H, H-3″), 7.47 (t, *J* = 1.8 Hz, 1H, H-2′), 7.28 (t, *J* = 7.9 Hz, 1H, H-5′), 7.04 (dt, *J* = 7.9, 1.8 Hz, 1H, H-6′), 4.00 (s, 2H, H-9), 2.18 (s, 3H, CH_3_) ppm; ^13^C NMR (100 MHz, DMSO-*d*_6_): δ = 184.6 (C-4), 183.9 (C-1), 161.2 (CO(NH)), 160.8 (d, ^1^*J*_C,F_ = 255.1 Hz, C-2″), 144.3 (C-2, C-3), 138.8 (C-1′), 138.7 (C-3′), 134.0 (C-6, C-7), 131.6 * (C-4a), 131.4 * (C-8a), 129.6 (m, C-4″), 128.9 (C-5′), 127.3 (p, ^3^*J*_C,F_ = 3.9 Hz, C-6″), 126.0 * (C-5), 125. 9 * (C-8), 125.9 (d, ^2^*J*_C,F_ = 17.3 Hz, C-1″), 125.3 (qd, ^2^*J*_C,F_ = 32.6, ^4^*J*_C,F_ = 3.3 Hz, C-5″), 124.3 (C-6′), 123.5 (q, ^1^*J*_C,F_ = 272.2 Hz, CF_3_), 119.2 (C-2′), 117.6 (C-4′), 117.5 (d, ^2^*J*_C,F_ = 23.3 Hz, C-3″), 31.7 (C-9), 13.0 (CH_3_) ppm; HRMS (APCI) calcd. for C_26_H_18_F_4_NO_3_ [M + H]^+^ = 468.1217; found: 468.1202.

*4-Fluoro-N-{3-[(3-methyl-1,4-dioxo-1,4-dihydronaphthalen-2-yl)methyl]phenyl}-2-(trifluoromethyl)benzamide (***3g***).* Compound **3g** was obtained after stirring for 6 h, then purified by flash chromatography using cyclohexane:CH_2_Cl_2_:EtOAc (5:3:1). Yellow solid; yield: 81%; *R*_f_ = 0.43 (cyclohexane:CH_2_Cl_2_:EtOAc = 5:3:1); m.p.: 179–180 °C. ^1^H NMR (400 MHz, DMSO-*d*_6_): δ = 10.46 (s, 1H, NH), 8.01 (m, 2H, H-5, H-8), 7.84 (m, 2H, H-6, H-7), 7.76 (m, 1H, H-3″), 7.75 (m, 1H, H-6″), 7.63 (m, 1H, H-5″), 7.62 (dd, *J* = 8.3, 2.3 Hz, 1H, H-4″), 7.41 (t, *J* = 1.9 Hz, 1H, H-2′), 7.26 (t, *J* = 7.9 Hz, 1H, H-5′), 7.01 (d, *J* = 7.9 Hz, 1H, H-6′), 3.99 (s, 2H, H-9), 2.17 (s, 3H, CH_3_) ppm; ^13^C NMR (100 MHz, DMSO-*d*_6_): δ = 184.6 (C-4), 183.9 (C-1), 164.5 (CO(NH)), 161.8 (d, ^1^*J*_C,F_ = 248.6 Hz, C-4″), 144.4 * (C-2), 144.3 * (C-3), 139.0 (C-3′), 138.8 (C-1′), 134.0 * (C-6), 133.9 * (C-7), 132.7 (m, C-1″), 131.6 * (C-4a), 131.4 * (C-8a), 131.3 (d, ^3^*J*_C,F_ = 8.7 Hz, C-6″), 128.9 (C-5′), 128.1 (qd, ^2^*J*_C,F_ = 32.6, ^3^*J*_C,F_ = 8.1 Hz, C-2″), 126.0 * (C-5), 125.9 * (C-8), 124.0 (C-6′), 122.7 (qd, ^1^*J*_C,F_ = 274.1, ^4^*J*_C,F_ = 2.6 Hz, CF_3_), 119.4 (d, ^2^*J*_C,F_ = 21.2 Hz, C-5″), 119.1 (C-2′), 117.4 (C-4′), 114.0 (dq, ^2^*J*_C,F_ = 25.5, ^3^*J*_C,F_ = 5.0 Hz, C-3″), 31.6 (C-9), 13.0 (CH_3_) ppm; HRMS (ESI) calcd. for C_26_H_16_F_4_NO_3_ [M − H]^−^ = 466.1066; found: 466.1071.

*N-{3-[(3-Methyl-1,4-dioxo-1,4-dihydronaphthalen-2-yl)methyl]phenyl}-3-(trifluoromethoxy)benzamide (***3h***).* Compound **3h** was obtained after stirring for 4 h, then purified by flash chromatography using cyclohexane:CH_2_Cl_2_:EtOAc (5:3:1). Yellow solid; yield: 78%; *R*_f_ = 0.41 (cyclohexane:CH_2_Cl_2_:EtOAc = 5:3:1); m.p.: 116–117 °C. ^1^H NMR (400 MHz, DMSO-*d*_6_): δ = 10.29 (s, 1H, NH), 8.02 (m, 2H, H-5, H-8), 7.96 (m, 1H, H-6″), 7.85 (m, 3H, H-2″, H-6, H-7), 7.66 (m, 2H, H-4′, H-5″), 7.58 (d, *J* = 8.4 Hz, 1H, H-4″), 7.55 (s, 1H, H-2′), 7.27 (t, *J* = 7.9 Hz, 1H, H-5′), 7.03 (d, *J* = 7.9 Hz, 1H, H-6′), 4.00 (s, 2H, H-9), 2.19 (s, 3H, CH_3_) ppm; ^13^C NMR (100 MHz, DMSO-*d*_6_): δ = 184.6 (C-4), 183.9 (C-1), 163.8 (CO(NH)), 148.3 (q, ^3^*J*_C,F_ = 1.7 Hz, C-3″), 144.3 (C-2, C-3), 138.9 (C-3′), 138.6 (C-1′), 133.9 (C-6, C-7), 131.6 * (C-4a), 131.4 * (C-8a), 130.9 (C-1″), 130.5 (C-5″), 128.7 (C-5′), 126.7 (C-6″), 126.0 * (C-5), 125.9 * (C-8), 124.1 (C-6′), 123.9 (C-4″), 120.0 (C-2′, C-2″), 120.0 (q, ^1^*J*_C,F_ = 256.9 Hz, CF_3_), 118.4 (C-4′), 31.7 (C-9), 13.0 (CH_3_) ppm; HRMS (ESI) calcd. for C_26_H_17_F_3_NO_4_ [M − H]^−^ = 464.1110; found: 464.1115.

#### 3.1.4. General Synthetic Procedure for Benzamido–Naphthoquinones **5a,b** and **6a,b** (Route B)

AgNO_3_ (0.1 equiv.) and (NH_4_)_2_S_2_O_8_ (2 equiv.) were dissolved in water (4 mL), and a solution of 1,4-naphthoquinone (200 mg, 1 equiv.) and the respective benzamido-phenylacetic acid derivative (1.4 equiv.) in a 1:1 mixture of CH_3_CN/CH_2_Cl_2_ (4 mL) was quickly added. The two-phase mixture was heated to 80 °C and stirred until the TLC showed complete consumption of the starting material (3–4 h). After cooling to ambient temperature, the mixture was extracted three times with CH_2_Cl_2_ (30 mL). The combined organic layers were washed with H_2_O, dried over Na_2_SO_4_, and concentrated in vacuo to give a residue, which was purified by flash chromatography as detailed below.

*N-{4-[(1,4-Dioxo-1,4-dihydronaphthalen-2-yl)methyl]phenyl}benzamide (***5a***).* Compound **5a** was obtained after stirring for 3 h, then purified by flash chromatography using cyclohexane:CH_2_Cl_2_:EtOAc (5:3:1). Amber solid; yield: 59%; *R*_f_ = 0.58 (cyclohexane:CH_2_Cl_2_:EtOAc = 5:3:1); m.p.: 190–192 °C. ^1^H NMR (400 MHz, CDCl_3_): δ = 9.95 (s, 1H, NH), 8.10 (m, 1H, H-8), 8.04 (m, 1H, H-5), 7.87 (m, 2H, H-2″, H-6″), 7.73 (m, 2H, H-6, H-7), 7.62 (d, *J* = 7.7 Hz, 2H, H-3′, H-5′), 7.54 (m, 1H, H-4″), 7.48 (m, 2H, H-3″, H-5″), 7.25 (d, *J* = 7.7 Hz, 2H, H-2′, H-6′), 6.63 (s, 1H, H-3), 3.89 (s, 2H, H-9) ppm; ^13^C NMR (100 MHz, CDCl_3_): δ = 185.1 (C-4), 185.0 (C-1), 165.7 (CO(NH)), 150.8 (C-2), 136.9 (C-4′), 135.6 (C-3), 134.9 (C-1″), 133.8 (C-6, C-7), 132.9 (C-1′), 132.2 (C-8a), 132.1 (C-4a), 131.9 (C-4’′), 130.1 (C-2′, C-6′), 128.8 (C-3″, C-5″), 127.0 (C-2″, C-6″), 126.7 (C-8), 126.1 (C-5), 120.6 (C-3′, C-5′), 35.3 (C-9) ppm; HRMS (ESI) calcd. for C_24_H_18_NO_3_ [M + H]^+^ = 368.1287; found: 368.1277.

*N-{4-[(1,4-Dioxo-1,4-dihydronaphthalen-2-yl)methyl]phenyl}-4-(trifluoromethyl)benzamide (***5b***).* Compound **5b** was obtained after stirring for 4 h, then purified by flash chromatography using cyclohexane:CH_2_Cl_2_:EtOAc (5:3:1). Amber solid; yield: 54%; *R*_f_ = 0.50 (cyclohexane:CH_2_Cl_2_:EtOAc = 5:3:1); m.p.: 201–202 °C. ^1^H NMR (400 MHz, DMSO-*d*_6_): δ = 10.45 (s, 1H, NH), 8.13 (d, *J* = 8.1 Hz, 2H, H-2″, H-6″), 8.02 (m, 1H, H-8), 7.96 (m, 1H, H-5), 7.87 (m, 4H, H-6, H-7, H-3″, H-5″), 7.73 (d, *J* = 8.6 Hz, 2H, H-3′, H-5′), 7.30 (d, *J* = 8.6 Hz, 2H, H-2′, H-6′), 6.75 (s, 1H, H-3), 3.86 (s, 2H, H-9) ppm; ^13^C NMR (100 MHz, DMSO-*d*_6_): δ = 185.2 (C-4), 184.9 (C-1), 164.8 (CO(NH)), 151.0 (C-2), 139.2 (C-1″), 137.8 (C-4′), 135.7 (C-3), 134.6 (C-6, C-7), 133.5 (C-1′), 131.8 (q, ^2^*J*_C,F_ = 31.9 Hz, C-4″) 132.2 (C-8a), 131.1 (C-4a), 130.6 (C-2″, C-6″), 130.0 (C-2′, C-6′), 126.7 (C-8), 126.1 (C-5), 125.8 (q, ^3^*J*_C,F_ = 3.8 Hz, C-3″, C-5″), 124.3 (q, ^1^*J*_C,F_ = 272.5 Hz, CF_3_), 121.1 (C-3′, C-5′), 34.9 (C-9) ppm; HRMS (ESI) calcd. for C_25_H_15_F_3_NO_3_ [M − H]^−^ = 434.1004; found: 434.1012.

*N-{3-[(1,4-Dioxo-1,4-dihydronaphthalen-2-yl)methyl]phenyl}benzamide (***6a***).* Compound **6a** was obtained after stirring for 3 h, then purified by flash chromatography using cyclohexane:CH_2_Cl_2_:EtOAc (5:3:1). Amber solid; yield: 55%; *R*_f_ = 0.58 (cyclohexane:CH_2_Cl_2_:EtOAc = 5:3:1); m.p.: 184–186 °C. ^1^H NMR (400 MHz, CDCl_3_): δ = 10.22 (s, 1H, NH), 8.01 (m, 1H, H-8), 7.97 (m, 1H, H-5), 7.92 (m, 2H, H-2″, H-6″), 7.86 (m, 2H, H-6, H-7), 7.70 (d, *J* = 8.1 Hz, 1H, H-4′), 7.68 (s, 1H, H-2′), 7.57 (m, 1H, H-4″), 7.51 (m, 2H, H-3″, H-5″), 7.31 (t, *J* = 8.1 Hz, 1H, H-5′), 7.06 (d, *J* = 7.6 Hz, 1H, H-6′), 6.81 (s, 1H, H-3), 3.88 (s, 2H, H-9) ppm; ^13^C NMR (100 MHz, CDCl_3_): δ = 184.6 (C-4), 184.3 (C-1), 165.5 (CO(NH)), 150.2 (C-2), 139.3 (C-3′), 137.9 (C-1′), 135.4 (C-3), 134.8 (C-1″), 134.1 (C-6, C-7), 131.7 * (C-8a), 131.5 * (C-4a), 131.5 (C-4″), 128.8 (C-5′), 128.3 (C-3″, C-5″), 127.6 (C-2″, C-6″), 126.2 (C-8), 125.6 (C-5), 124.5 (C-6′), 120.8 (C-2′), 118.6 (C-4′), 35.0 (C-9) ppm; HRMS (ESI) calcd. for C_24_H_18_NO_3_ [M + H]^+^ = 368.1287; found: 368.1276.

*N-{3-[(1,4-Dioxo-1,4-dihydronaphthalen-2-yl)methyl]phenyl}-4-(trifluoromethyl)benzamide (***6b***).* Compound **6b** was obtained after stirring for 4 h, then purified by flash chromatography using cyclohexane:CH_2_Cl_2_:EtOAc (5:3:1). Amber solid; yield: 53%; *R*_f_ = 0.50 (cyclohexane:CH_2_Cl_2_:EtOAc = 5:3:1); m.p.: 205–206 °C. ^1^H NMR (400 MHz, CDCl_3_): δ = 8.08 (m, 1H, H-8), 8.02 (m, 1H, H-5), 7.96 (d, *J* = 7.8 Hz, 2H, H-2″, H-6″), 7.72 (m, 2H, H-6, H-7), 7.71 (d, *J* = 8.0 Hz, 2H, H-3″, H-5″), 7.57 (m, 1H, H-2′), 7.56 (d*, J* = 7.7 Hz, 1H, H-4′), 7.33 (t, *J* = 7.7 Hz, 1H, H-5′), 7.06 (dt, *J* = 7.7 Hz, 1.4 Hz, 1H, H-6′), 6.63 (t, *J* = 1.5 Hz, 1H, H-3), 3.89 (d, *J* = 1.5 Hz, 2H, H-9) ppm; ^13^C NMR (100 MHz, CDCl_3_): δ = 185.1 (C-4), 184.9 (C-1), 164.5 (CO(NH)), 150.4 (C-2), 138.1 (q, ^5^*J*_C,F_ = 1.2 Hz, C-1″), 138.0 (C-1′, C-3′), 135.7 (C-3), 133.9 * (C-6), 133.8 * (C-7), 132.1 * (C-4a), 132.0 * (C-8a), 129.7 (C-5′), 127.6 (C-2″, C-6″), 126.7 (C-8), 126.1 (C-5), 126.0 (C-6′), 125.8 (q, ^3^*J*_C,F_ = 3.9 Hz, C-3″, C-5″), 123.6 (q, ^1^*J*_C,F_ = 272.6 Hz, CF_3_), 121.2 (C-2′), 119.0 (C-4′), 35.7 (C-9) ppm; HRMS (ESI) calcd. for C_25_H_17_F_3_NO_3_ [M + H]^+^ = 436.1161; found: 436.1146.

## 4. Conclusions

The present study describes the effective synthesis of 21 plasmodione-like benzamido–naphthoquinone derivatives, which exhibit remarkable antiplasmodial activity and selectivity. Utilizing advanced ligand efficiency metrics and structure–activity relationship analyses, we have identified highly effective and low-toxicity compounds, including the 2-fluoro-5-trifluoromethylbenzamido derivative **2f** or the 2,4,5-trifluorobenzamido derivative **2i**. The trypanocidal activity of the synthesized compounds was also evaluated. The strongest activity was observed in benzamido-NQ **5a**, although both activity and selectivity were significantly less pronounced.

These findings highlight a promising pathway for developing new antiplasmodial drugs with superior efficacy and safety profiles. They also provide a robust framework for optimizing the drug discovery processes through the systematic application of physicochemical parameters.

## Figures and Tables

**Table 1:** Synthesis of substituted benzamido–menadione and naphthoquinone derivatives (overall yields calculated from MD and NQ).

Compd	Structure	Route	Yield (%)	Compd	Structure	Route	Yield (%)
**2a**	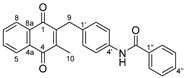	A	79	**3c**	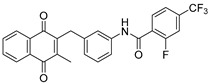	A	69
**2b**	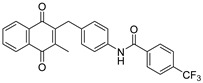	A	72	**3d**	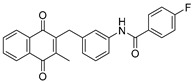	A	71
**2c**	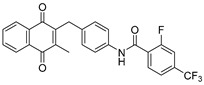	A	81	**3e**	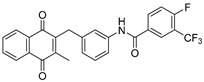	A	83
**2d**	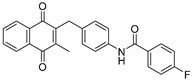	A	76	**3f**	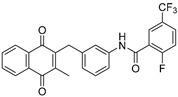	A	62
**2e**	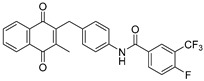	A	65	**3g**	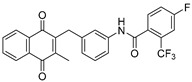	A	81
**2f**	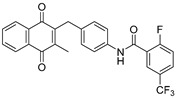	A	62	**3h**	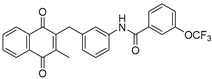	A	78
**2g**	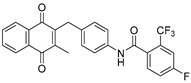	A	81	**5a**	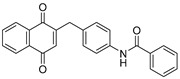	B	59
**2h**	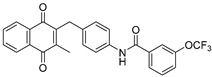	A	75	**5b**	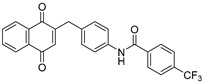	B	54
**2i**	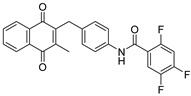	A	61	**6a**	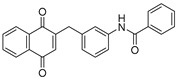	B	55
**3a**	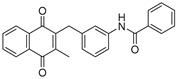	A	72	**6b**	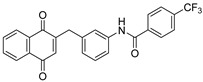	B	53
**3b**	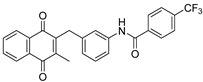	A	72				

**Table 2:** In vitro antiparasitic activity, mammalian cell toxicity, and predicted oral toxicity of the synthesized compounds.

Compd	*P.falc.* ^1^	SI ^2^	*P.falc.* ^1^	*T.b.rhod.* ^3^	SI ^2^	*T.b.rhod.* ^3^	Cyt L6 ^4^	Oral Toxicity ^6^
	IC_50_ μM		pIC_50_^5^	IC_50_ μM		pIC_50_^5^	IC_50_ μM	LD_50_ mg/kg
Chl.	0.009	10,000	8.03				90	750
Mel.				0.015	12,000	7.82	180	3000
Pod.							0.019	100
**2a**	0.336	471	6.47	64.101	2	4.19	158.17	4000
**2b**	0.136	1639	6.87	34.582	6	4.46	222.51	1000
**2c**	0.045	4762	7.35	160.627	1	3.79	213.94	1000
**2d**	0.663	377	6.18	84.442	3	4.07	250.36	4000
**2e**	0.053	2914	7.27	8.911	**17**	5.05	155.88	1000
**2f**	**0.021**	**10,000**	**7.67**	163.537	1	3.79	213.94	1000
**2g**	0.026	5580	7.59	32.324	4	4.49	143.26	4000
**2h**	0.067	1685	7.18	129.636	1	3.89	112.22	57
**2i**	0.023	10,000	7.64	171.796	1	3.76	229.67	1600
**3a**	1.487	88	5.83	65.333	2	4.18	130.95	4000
**3b**	0.265	453	6.58	27.689	4	4.56	119.82	4000
**3c**	0.167	525	6.78	65.344	1	4.18	87.57	4000
**3d**	3.252	69	5.49	53.628	4	4.27	225.33	4000
**3e**	1.012	211	5.99	50.383	4	4.30	213.94	4000
**3f**	0.449	476	6.35	43.195	5	4.36	213.94	4000
**3g**	14.462	2	4.84	26.069	1	4.58	30.55	1000
**3h**	2.810	76	5.55	94.679	2	4.02	214.86	4000
**5a**	4.556	6	5.34	**1.900**	14	**5.72**	25.91	4000
**5b**	14.975	6	4.82	15.016	6	4.82	83.62	1000
**6a**	6.192	3	5.21	3.503	4	5.46	15.59	4000
**6b**	2.104	7	5.68	5.234	3	5.28	14.05	4000

^1^ *P. falciparum*, strain NF54, erythrocytic stages; ^2^ SI is defined as the following ratio: IC_50_ in L6 cells/IC_50_ in each parasite; ^3^ *T. brucei rhodesiense*, strain STIB900 trypomastigote bloodstream forms; ^4^ cytotoxicity L6 cells rat skeletal myoblasts; ^5^ pIC_50_ = −log_10_ IC_50_ (M); ^6^ the oral toxicity was calculated using the ProTox 3.0 software (https://tox.charite.de/protox3/ (accessed on 4 July 2025)), and the calculated median lethal dose (LD_50_) is given in mg/kg body weight. Reference drugs: chloroquine (chl.), melarsoprol (mel.), and podophyllotoxin (pod.). Values in bold highlight the most striking results.

**Table 3:** Important ligand efficiency metrics and composite physicochemical descriptors, as well as their corresponding algebraic expressions and descriptions.

Variable Name	Definition
LE	1.37 × (pIC_50_/NHA)
SILE	pIC_50_/NHA^0.3^
FQ	(pIC_50_/NHA)/[0.0715 + (7.5328/NHA) + (25.7079/NHA^2^) − (361.4722/NHA^3^)]
BEI	(pIC_50_ × 1000)/MW
SEI	(pIC_50_ × 100 Å^2^)/tPSA
LLE (or LipE)	pIC_50_ − logP (or logD_7.4_)
LLE_AT_	0.11 + [(1.37 × LLE)/NHA] = 0.11 + [(LE × LLE)/pIC_50_]
LELP	logP/LE
nBEI	pIC_50_ + log_10_NHA
NSEI	pIC_50_/NPOL(N+O)
PFI (SFI)	logD_7.4_ + #aromrings
AB-MPS	|logD_7.4_ − 3| + #rotbonds + #aromrings

## Data Availability

The data presented in this study are available on request from the corresponding author.

## Supplementary Materials

The following supporting information can be downloaded at https://www.mdpi.com/article/10.3390/ijms262210951/s1.

## Abbreviations

The following abbreviations are used in this manuscript:

AB-MPS	AbbVie’s multi-parameter score
BEI	Binding efficiency index
FQ	Fit quality
H2L	Hit-to-lead
LE	Ligand efficiency
LELP	Ligand efficiency lipophilicity price
LipE	Lipophilic efficiency
LLE	Lipophilic ligand efficiency
LLE_AT_	Astex lipophilic ligand efficiency
MD	Menadione
MW	Molecular weight
nBEI	Normalized binding efficiency index
NHA	Non-hydrogen atoms
NPOL	Number of oxygens and nitrogens in the structure
NQ	Naphthoquinone
NSEI	Normalized (polar) surface efficiency index
PFI	GSK property forecast index
Ro5	Rule of five
SEI	Surface efficiency index
SFI	Solubility forecast index
SILE	Size-independent ligand efficiency
TLC	Thin-layer chromatography
tPSA	Topological polar surface area
WHO	World Health Organization
