# Supplementary material for: Synthesis, Antiprotozoal Activity, and Physicochemical Evaluation of Benzamido–Menadione Derivatives
Source: Int J Mol Sci. 2025 Nov 12;26(22):10951. doi: 10.3390/ijms262210951 (PMC12652484; doi:10.3390/ijms262210951)

Synthesis, Antiprotozoal Activity and Physicochemical Evaluation of Benzamido-Menadione Derivatives

Armin Presser [1,*], Gregor Blaser [1], Eva-Maria Pferschy-Wenzig [2,5], Monica Cal [3,4], Pascal Mäser [3,4] and Wolfgang Schuehly [2]

1 Institute of Pharmaceutical Sciences, Pharmaceutical Chemistry, University of Graz, Schubertstrasse 1, A-8010 Graz, Austria
2 Institute of Pharmaceutical Sciences, Pharmacognosy, University of Graz, Universitaetsplatz 4, A-8010 Graz, Austria
3 Swiss Tropical and Public Health Institute, Kreuzstrasse 2, 4123 Allschwil, Switzerland
4 Faculty of Philosophy and Natural Sciences, University of Basel, Swiss TPH, Petersplatz 1, 4003 Basel, Switzerland
5 Field of Excellence BioHealth, University of Graz, Graz, Austria

Supplementary Material

Table of contents

1. Methodology for biological testing **S2**

2. Calculated physicochemical parameters **S3-S4**

3. Fully annotated correlation plots **S5-S7**

4. ^{1}H NMR and ^{13}C NMR spectra of the target compounds **S8-S28**

1. Methodology for biological testing

1.1 In vitro growth inhibition assay against Plasmodium falciparum NF54

Plasmodium falciparum, strain NF54, erythrocytic stages, and the standard drug, chloroquine, were used for the assay. The parasite cultures incubated in RPMI 1640 medium with 5% AlbuMAX™ (without hypoxanthine) were exposed to serial drug dilutions in microtiter plates. After 48 h of incubation at 37 °C under a reduced oxygen atmosphere, 0.5 μCi [^{3}H]-hypoxanthine was added to each well of the plate. Cultures were incubated for a further 24 h before they were harvested onto glass-fiber filters and washed with distilled water. The radioactivity was counted using a Betaplate™ liquid scintillation counter (Wallac, Zurich). The results were recorded as counts per minute (CPM) per well at each drug concentration and expressed as percentage of the untreated controls. IC_{50} values were calculated from the sigmoidal inhibition curves using Microsoft Excel. Chloroquine was used as control.

1.2 In vitro growth inhibition assay against Trypanosoma brucei rhodesiense

Trypanosoma brucei rhodesiense, STIB 900 strain, and the standard drug, melarsoprol, were used for the assay. Minimum Essential Medium (50 μL) supplemented with 25 mM HEPES, 1g/L additional glucose, 1% MEM non-essential amino acids (100x), 0.2 mM 2-mercaptoethanol, 1 mM Na-pyruvate and 15% heat-inactivated horse serum was added to each well of a 96-well microtiter plate. Serial drug dilutions of 11 three-fold dilution steps covering a range from 100 to 0.002 μg/mL were prepared. Then 4 x 10^3 bloodstream forms of *T. b. rhodesiense* (STIB 900) in 50 μL were added to each well and the plate incubated at 37 °C under a 5% CO_2 atmosphere for 72 h. 10 μL Alamar Blue (resazurin, 12.5 mg in 100 mL double-distilled water) was then added to each well and incubation continued for a further 2–4 h. Then, the plates were read using a Spectramax Gemini XS microplate fluorometer (Molecular Devices Cooperation, Sunnyvale, CA, USA) using an excitation wavelength of 536 nm and an emission wavelength of 588 nm. The IC_{50} values were calculated from the sigmoidal inhibition curves using the microplate reader software Softmax Pro (Molecular Devices Cooperation, Sunnyvale, CA, USA). Melarsoprol was used as control.

1.3 Cytotoxicity against L6 cells

Assays were performed in 96-well microtiter plates, each well containing 100 μL of RPMI 1640 medium supplemented with 1% L-glutamine (200 mM) and 10% foetal bovine serum, and 4000 L6 cells (a primary cell line derived from rat skeletal myoblasts). Serial drug dilutions of 11 threefold dilution steps covering a range from 100 to 0.002 μg/mL were prepared. After 72 h of incubation, the plates were inspected under an inverted microscope to assure growth of the controls and sterile conditions. 10 μL of Alamar Blue solution was then added to each well and the plates incubated for another 2 h. Then the plates were read with a Spectramax Gemini XS microplate fluorometer (Molecular Devices Cooperation, Sunnyvale, CA, USA) using an excitation wavelength of 536 nm and an emission wavelength of 588 nm. The IC_{50} values were calculated by linear regression from the sigmoidal dose inhibition curves using the microplate reader software Softmax Pro (Molecular Devices Cooperation, Sunnyvale, CA, USA). Podophyllotoxin (Sigma P4405) was used as control.

Table S1: Calculated ligand efficiency metrics of the tested compounds.

compd	Ligand efficiency metrics (*P. falciparum*)								Ligand efficiency metrics (*T. brucei rhodesiense)*							
	**LE** [1]	**LLE** [1]	**LELP** [1]	**SILE** [2]	**FQ** [2]	**LLE_{AT}** [2]	**nBEI** [2]	**NSEI** [2]	**LE** [1]	**LLE** [1]	**LELP** [1]	**SILE** [2]	**FQ** [2]	**LLE_{AT}** [2]	**nBEI** [2]	**NSEI** [2]
**2a**	0.3062	1.5645	16.030	2.3576	0.6434	0.1839	7.94	1.62	0.1984	-0.7161	24.749	1.5269	0.4167	0.0762	5.66	1.05
**2b**	0.2855	1.1090	20.170	2.4057	0.6642	0.1560	8.39	1.72	0.1855	-1.2963	31.045	1.5628	0.4315	0.0562	5.98	1.12
**2c**	0.2964	1.4885	19.762	2.5509	0.7060	0.1700	8.88	1.84	0.1531	-2.0641	38.266	1.3173	0.3646	0.0268	5.33	0.95
**2d**	0.2825	1.1685	17.732	2.2270	0.6097	0.1634	7.66	1.54	0.1863	-0.9366	26.896	1.4683	0.4020	0.0672	5.55	1.02
**2e**	0.2936	1.4174	19.955	2.5246	0.6987	0.1671	8.80	1.82	0.2038	-0.8082	28.750	1.7533	0.4852	0.0774	6.58	1.26
**2f**	0.3098	1.8195	18.910	2.6628	0.7370	0.1833	9.20	1.92	0.1528	-2.0719	38.345	1.3146	0.3638	0.0265	5.32	0.95
**2g**	0.3061	1.7267	19.142	2.6353	0.7294	0.1796	9.12	1.90	0.1812	-1.3678	32.333	1.5590	0.4315	0.0549	6.02	1.12
**2h**	0.2895	1.1657	20.756	2.4915	0.6896	0.1570	8.71	1.44	0.1569	-2.1209	38.306	1.3496	0.3735	0.0245	5.42	0.78
**2i**	0.3275	2.4267	15.915	2.7008	0.7437	0.2139	9.14	1.91	0.1614	-1.4466	32.288	1.3311	0.3666	0.0481	5.27	0.94
**3a**	0.2757	0.9185	17.807	2.1222	0.5791	0.1534	7.29	1.46	0.1980	-0.7243	24.798	1.5239	0.4159	0.0758	5.65	1.05
**3b**	0.2734	0.8193	21.058	2.3040	0.6361	0.1440	8.10	1.64	0.1895	-1.1998	30.387	1.5966	0.4408	0.0602	6.08	1.14
**3c**	0.2735	0.9190	21.423	2.3531	0.6512	0.1470	8.31	1.69	0.1689	-1.6735	34.694	1.4529	0.4021	0.0426	5.72	1.05
**3d**	0.2510	0.4779	19.964	1.9782	0.5416	0.1318	6.96	1.37	0.1953	-0.7394	25.654	1.5394	0.4215	0.0762	5.75	1.07
**3e**	0.2419	0.1365	24.219	2.0813	0.5760	0.1155	7.53	1.50	0.1734	-1.5606	33.783	1.4921	0.4130	0.0471	5.83	1.07
**3f**	0.2561	0.4895	22.873	2.2037	0.6099	0.1297	7.88	1.59	0.1761	-1.4937	33.265	1.5153	0.4194	0.0498	5.90	1.09
**3g**	0.1953	-1.0185	29.999	1.6803	0.4650	0.0690	6.37	1.21	0.1850	-1.2744	31.674	1.5914	0.4405	0.0586	6.12	1.15
**3h**	0.2240	-0.4569	26.823	1.9273	0.5334	0.0916	7.08	1.11	0.1624	-1.9845	37.006	1.3970	0.3866	0.0300	5.56	0.80
**5a**	0.1939	-1.1126	32.708	1.7703	0.4930	0.0688	6.80	1.31	0.1994	-0.9649	31.810	1.8203	0.5069	0.0743	6.95	1.34
**5b**	0.2552	0.7392	17.513	1.9166	0.5212	0.1462	6.66	1.30	0.2673	0.9866	16.719	2.0077	0.5460	0.1583	6.90	1.36
**6a**	0.2617	0.8724	17.076	1.9657	0.5346	0.1527	6.79	1.34	0.2803	1.2522	15.943	2.1054	0.5726	0.1713	7.17	1.43
**6b**	0.2068	-0.4927	25.708	1.7058	0.4697	0.0889	6.33	1.21	0.2068	-0.4939	25.714	1.7053	0.4696	0.0889	6.33	1.21

[1] The ligand efficiency (LE), lipophilic ligand efficiency (LLE) and ligand efficiency lipophilic price (LELP) are based on IC_{50} data in nmol/L, these values and the molecular weight (MW) were calculated using the DataWarrior software, version 6.05.02 (https://openmolecules.org/datawarrior/index.html); [2] The values for the size-independent ligand efficiency (SILE), the fit quality (FQ), the Astex lipophilic ligand efficiency (LLE_{AT}), the normalized binding efficiency index (nBEI) and the normalized (polar) surface efficiency index (NSEI) were calculated according to the corresponding formulae presented in Table 3.

Table S2: Calculated physicochemical properties of the tested compounds.

compd	MW [1]	log*P* [2]	log$D_{7.4}$ [2]	TPSA [2]	#HBD [1]	#HBA [1]	NHA [1]	pKa (basic) [2]	PFI [3]	AB-MPS [3]
**2a**	381.43	4.41	3.91	63.24	1	4	29	3.58	6.91	7.91
**2b**	449.43	4.86	3.97	63.24	1	4	33	3.76	6.97	8.97
**2c**	467.42	5.05	4.08	63.24	1	4	34	3.19	7.08	9.08
**2d**	399.42	4.47	3.73	63.24	1	4	30	3.23	6.73	7.73
**2e**	467.42	4.98	4.01	63.24	1	4	34	3.63	7.01	9.01
**2f**	467.42	4.96	4.05	63.24	1	4	34	3.31	7.05	9.05
**2g**	467.42	4.87	4.06	63.24	1	4	34	3.51	7.06	9.06
**2h**	465.43	5.31	4.15	72.47	1	5	34	3.42	7.15	10.15
**2i**	435.40	4.75	3.76	63.24	1	4	32	3.18	6.76	7.76
**3a**	381.43	4.31	3.95	63.24	1	4	29	3.83	6.95	7.95
**3b**	449.43	4.84	3.97	63.24	1	4	33	3.71	6.97	8.97
**3c**	467.42	4.96	4.08	63.24	1	4	34	3.12	7.08	9.08
**3d**	399.42	4.37	3.79	63.24	1	4	30	3.43	6.79	7.79
**3e**	467.42	4.92	4.01	63.24	1	4	34	3.65	7.01	9.01
**3f**	467.42	4.86	4.04	63.24	1	4	34	3.23	7.04	9.04
**3g**	467.42	4.76	4.02	63.24	1	4	34	3.49	7.02	9.02
**3h**	465.43	5.27	4.13	72.47	1	5	34	3.37	7.13	10.13
**5a**	521.39	5.26	4.49	63.24	1	4	37	2.57	7.49	10.49
**5b**	367.40	3.47	3.49	63.24	1	4	28	3.08	6.49	7.49
**6a**	367.40	3.55	3.55	63.24	1	4	28	2.63	6.55	7.55
**6b**	435.40	4.40	3.80	63.24	1	4	32	3.37	6.80	8.80

[1] The molecular weight (MW), the number of H-bond donor (#HBD, NH/OH count), the number of H-bond acceptor (#HBA, N/O count) and the number of non-hydrogen atoms (NHA) were calculated using the DataWarrior software, version 6.05.02 (https://openmolecules.org/datawarrior/index.html); [2] The log*P*, log$D_{7.4}$, topological polar surface area (TPSA) and pKa (basic) were calculated using the ADMETlab 3.0 software (https://admetlab3.scbdd.com), the pKa (basic) denotes the pKa value of basic sites, with higher values indicating weaker acidity of the conjugate acid and stronger basicity of the corresponding site; [3] The Property Forecast Index (PFI) and the AbbVie Multi-Parameter Score (Abb-MPS) were calculated as shown in Table 3.

Figure S1. Fully annotated scatterplot of LLE vs SILE for the synthesized compounds including some optimization trajectories

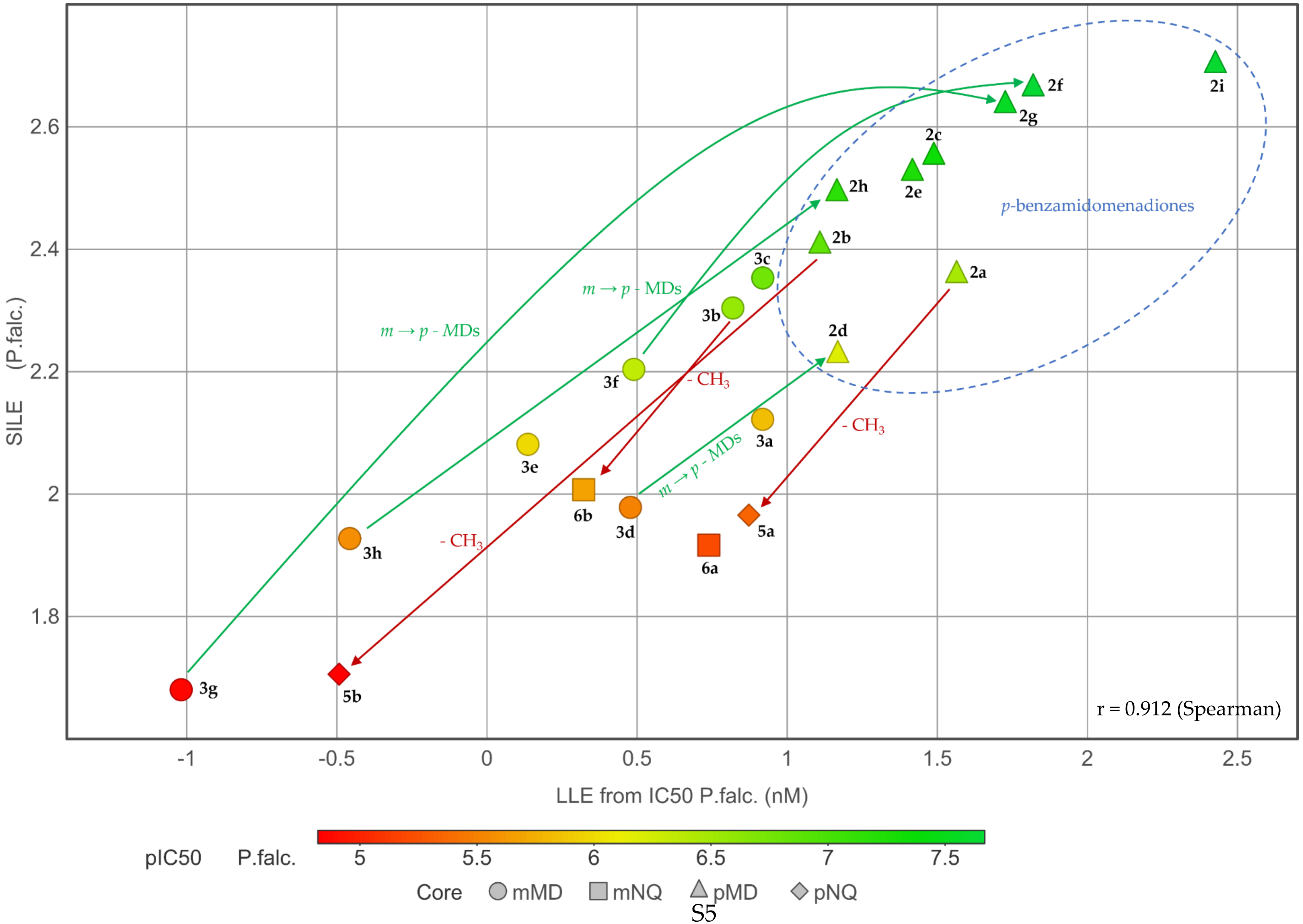

Figure S2. Fully annotated scatterplot of LLE vs FQ for the synthesized compounds including some optimization trajectories

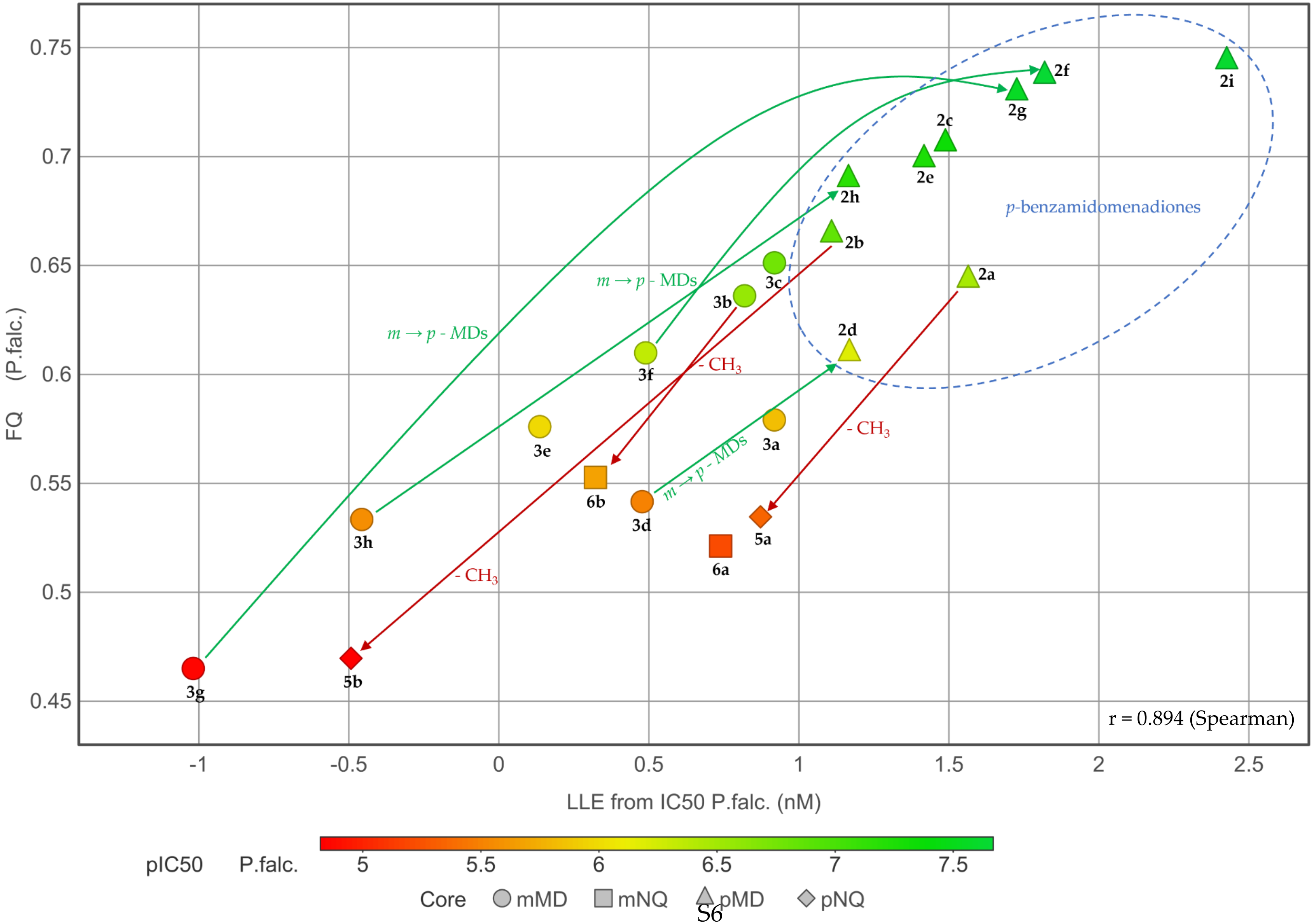

Figure S3. Fully annotated scatterplot of NSEI vs nBEI for the synthesized compounds including some optimization trajectorie

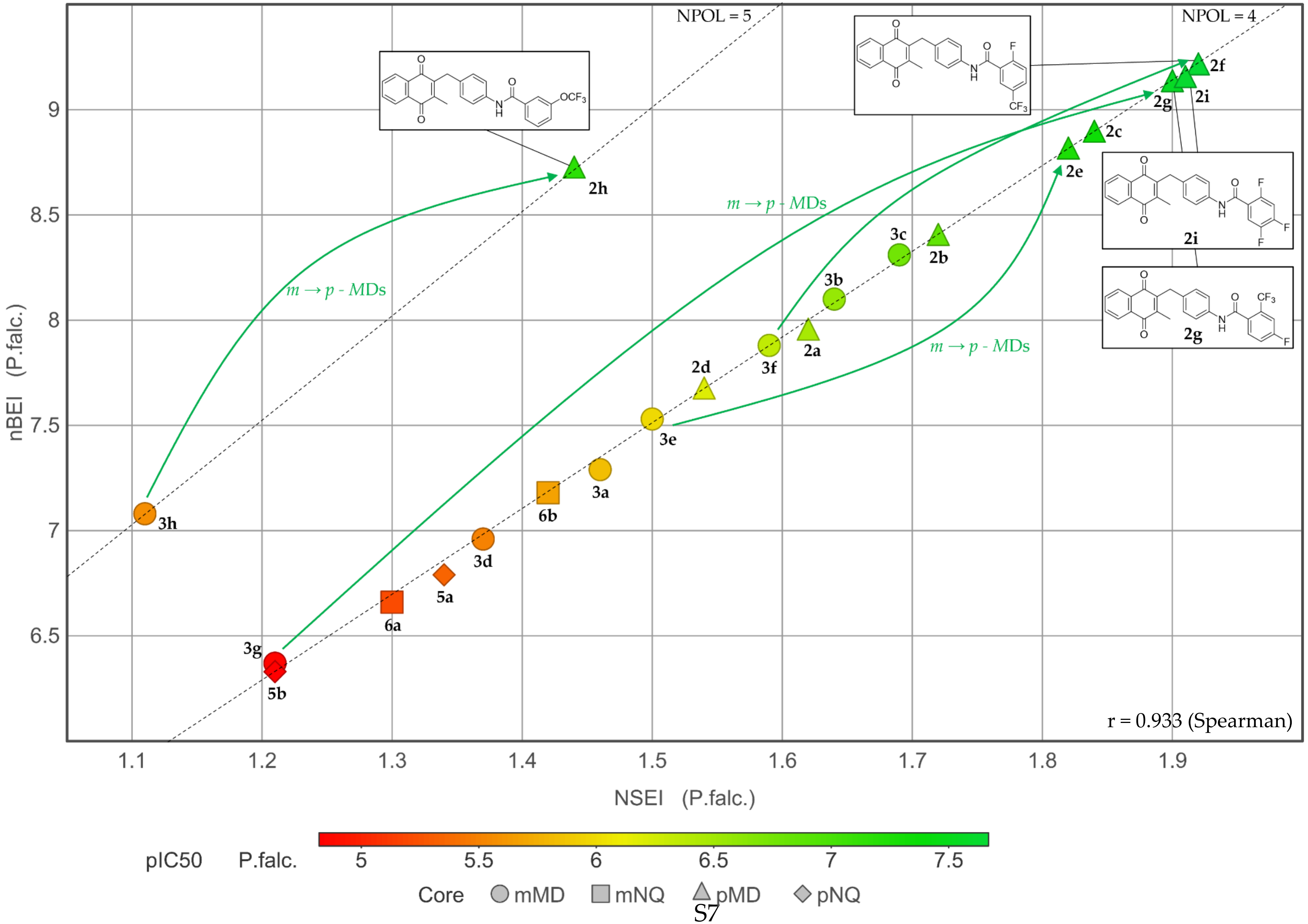

^{1}H- and ^{13}C-NMR of the target compounds

N-{4-[(3-Methyl-1,4-dioxo-1,4-dihydronaphthalen-2-yl)methyl]phenyl}benzamide (**2a**)

^{1}H NMR

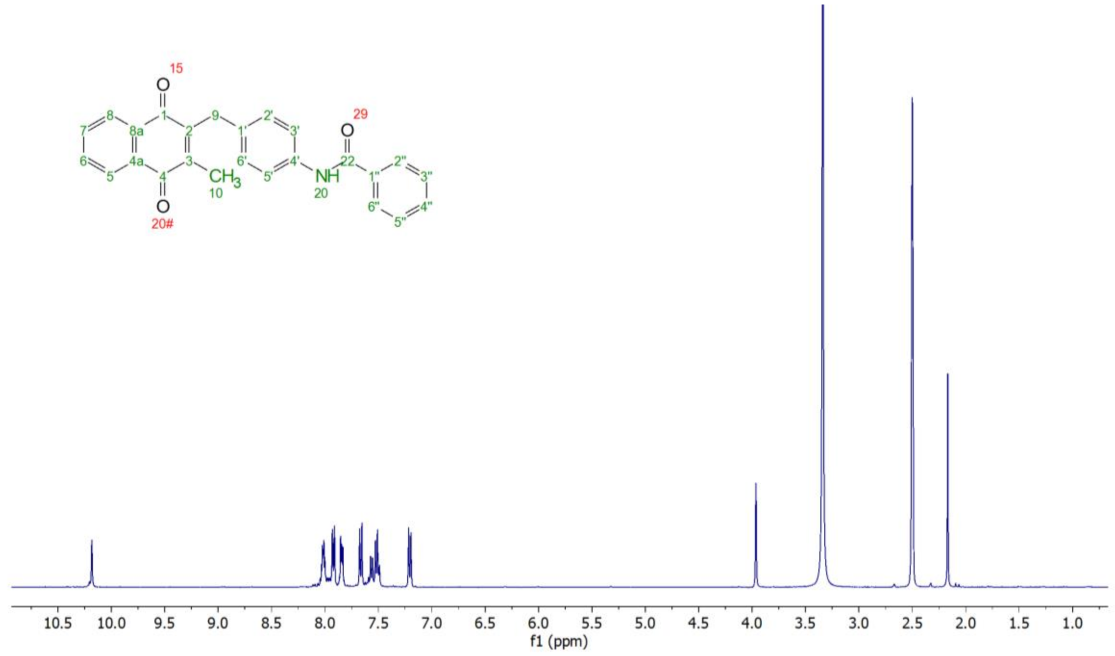

^{13}C NMR

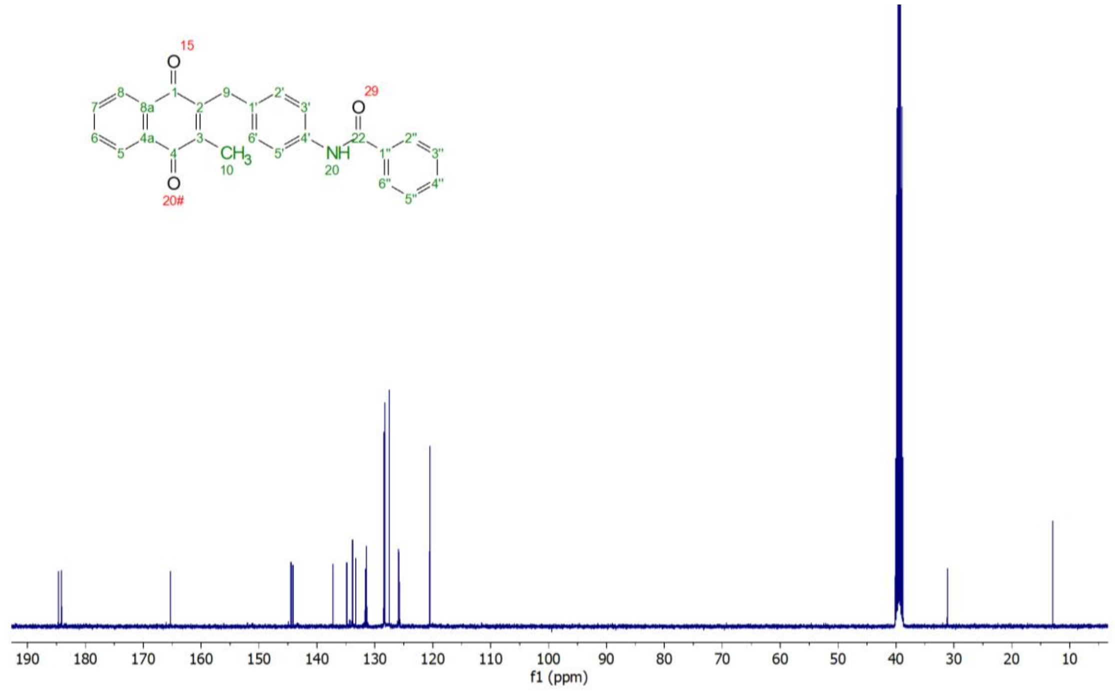

N-{4-[(3-Methyl-1,4-dioxo-1,4-dihydronaphthalen-2-yl)methyl]phenyl}-4-(trifluoromethyl)benzamide (**2b**)

^{1}H NMR

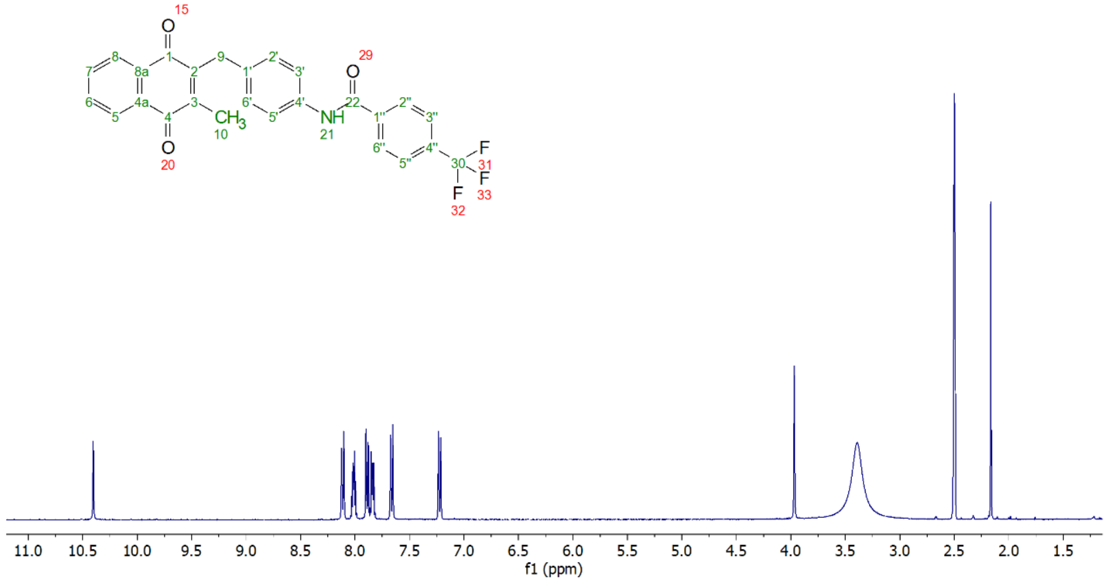

^{13}C NMR

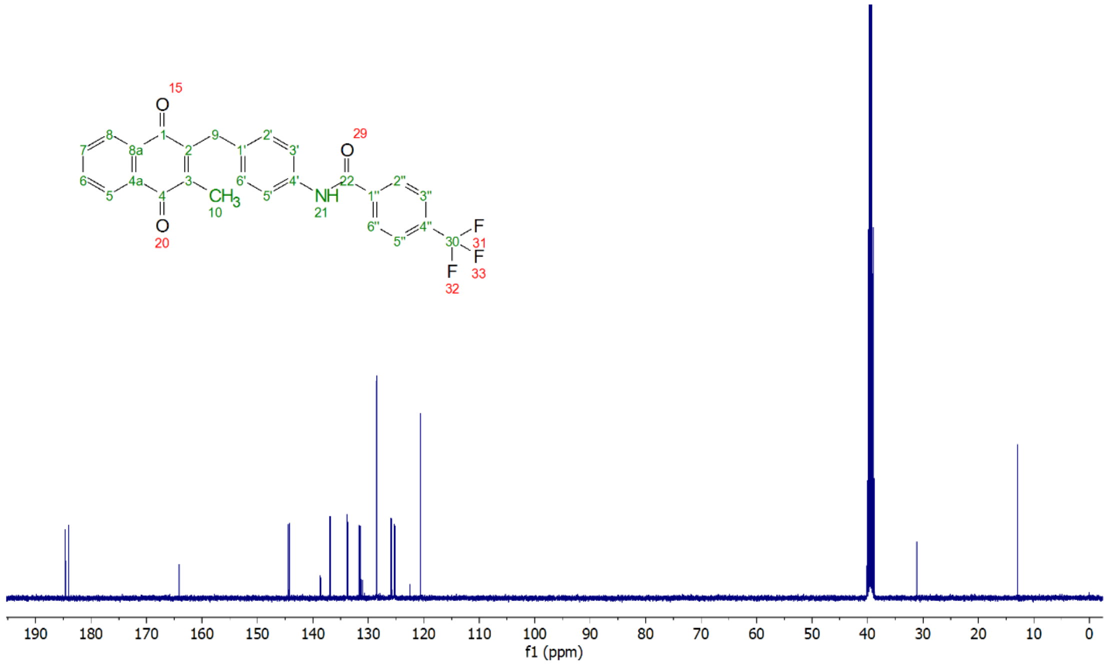

2-Fluoro-N-{4-[(3-methyl-1,4-dioxo-1,4-dihydronaphthalen-2-yl)methyl]phenyl}-4-(trifluoromethyl)benzamide (**2c**)

^{1}H NMR

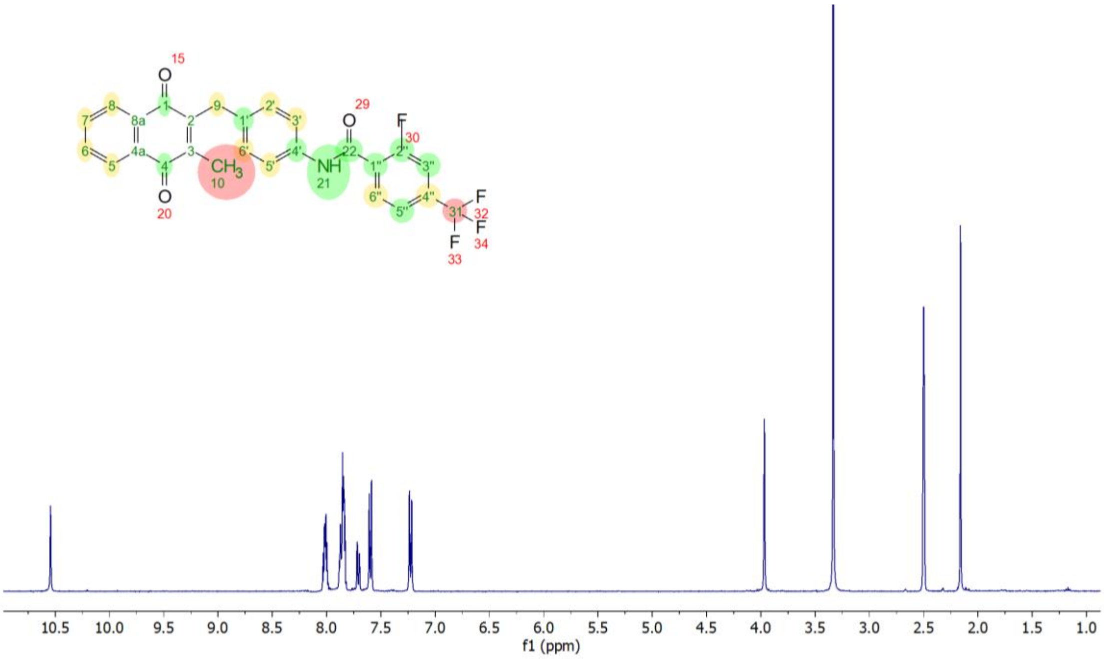

^{13}C NMR

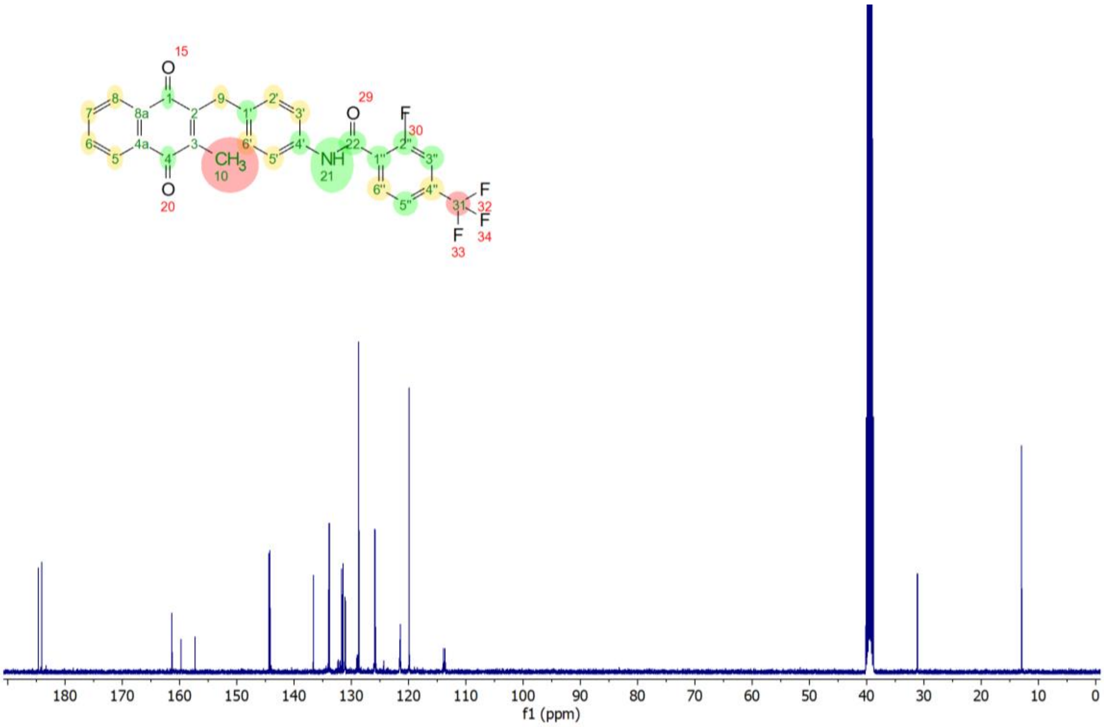

4-Fluoro-N-{4-[(3-methyl-1,4-dioxo-1,4-dihydronaphthalen-2-yl)methyl]phenyl}benzamide (**2d**)

^{1}H NMR

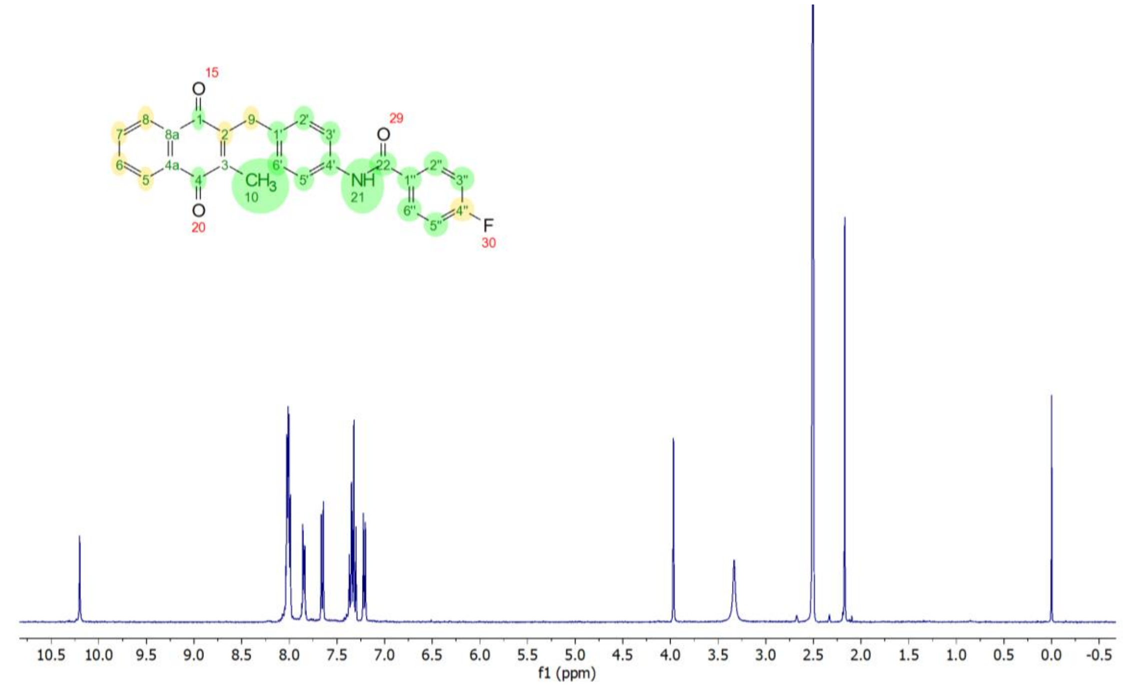

^{13}C NMR

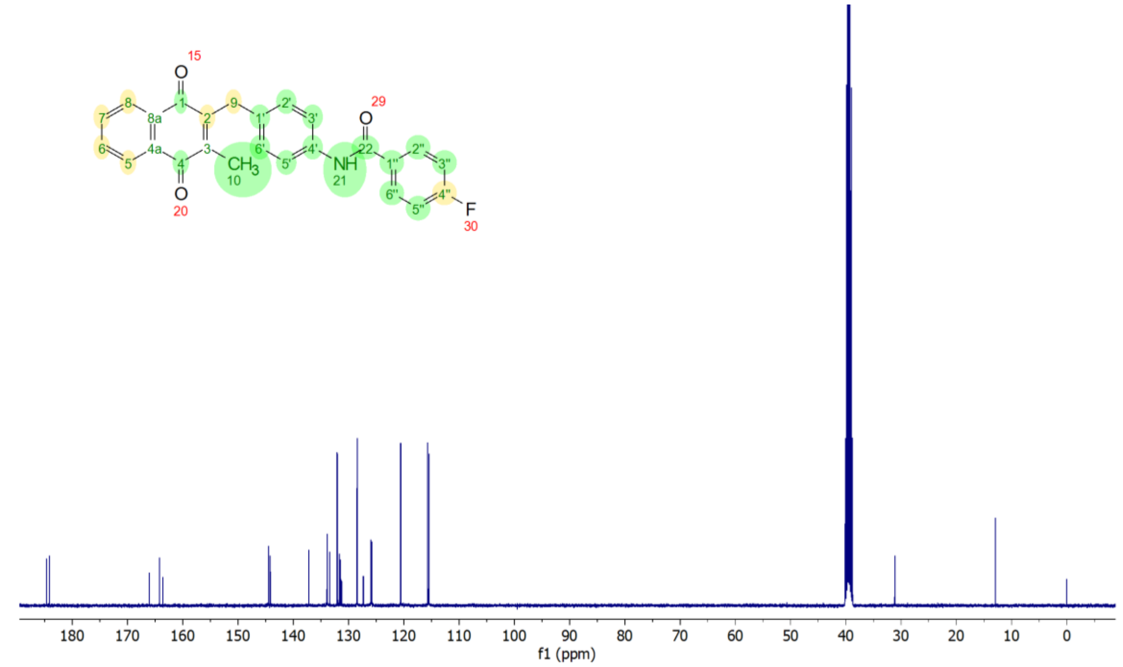

4-Fluoro-N-{4-[(3-methyl-1,4-dioxo-1,4-dihydronaphthalen-2-yl)methyl]phenyl}-3-(trifluoromethyl)benzamide (**2e**)

^{1}H NMR

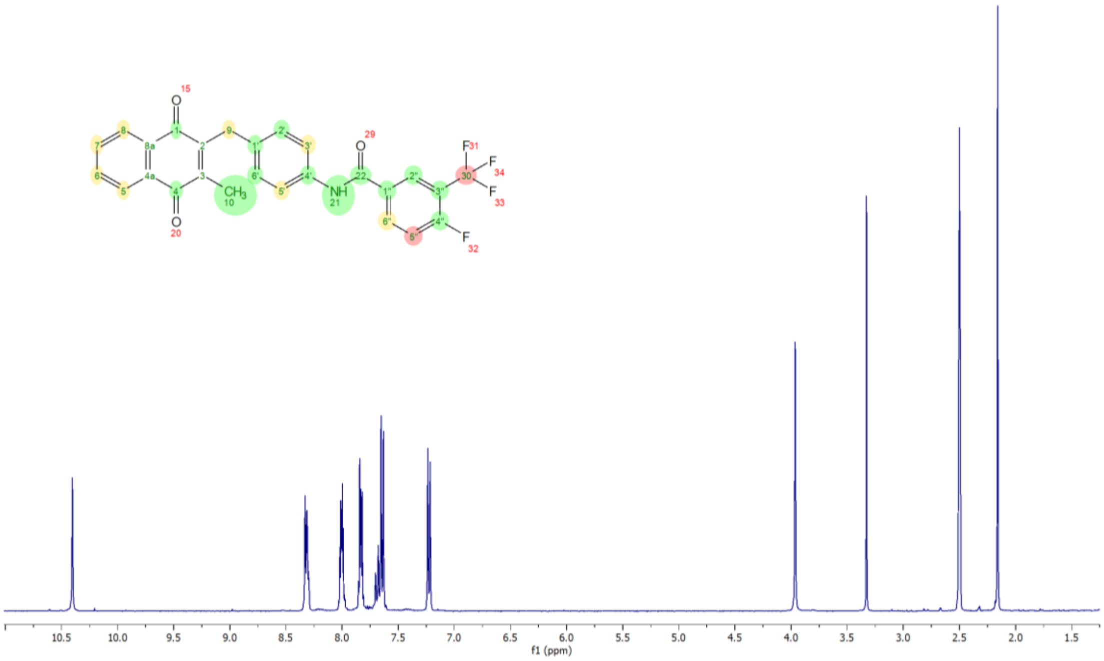

^{13}C NMR

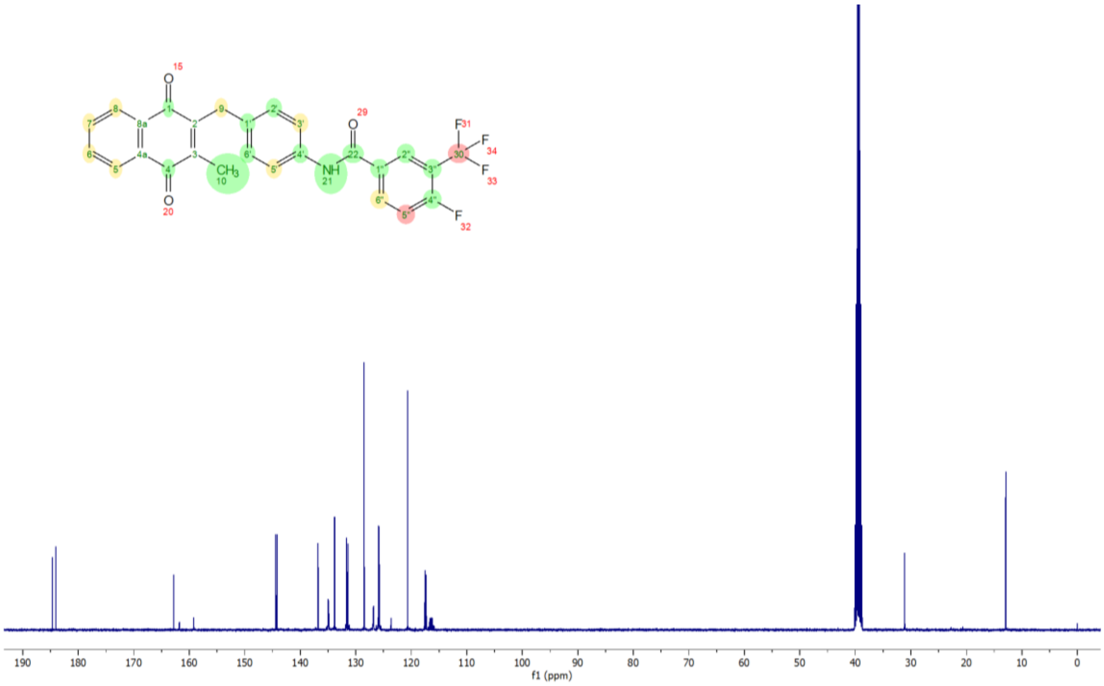

2-Fluoro-N-{4-[(3-methyl-1,4-dioxo-1,4-dihydronaphthalen-2-yl)methyl]phenyl}-5-(trifluoromethyl)benzamide (**2f**)

^{1}H NMR

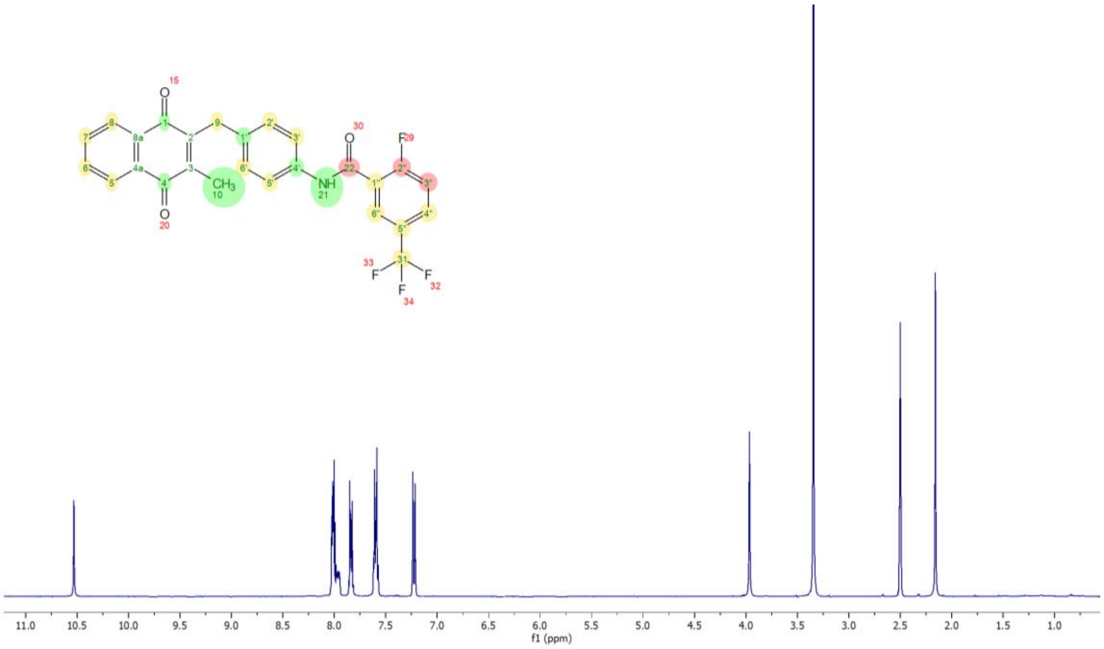

^{13}C NMR

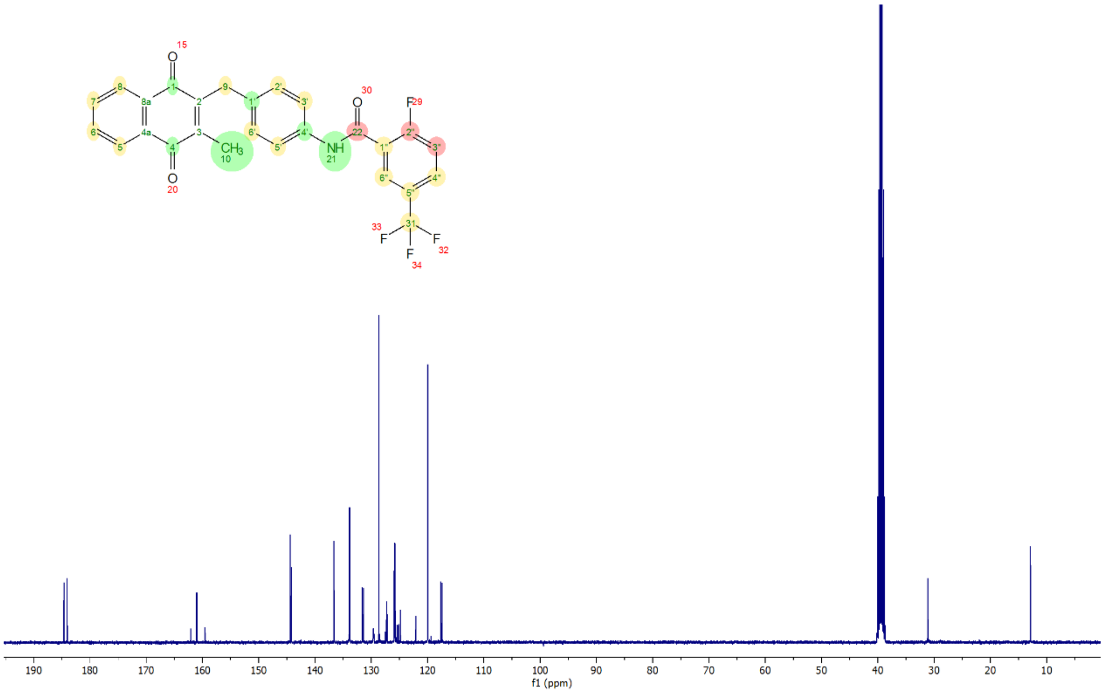

4-Fluoro-N-{4-[(3-methyl-1,4-dioxo-1,4-dihydronaphthalen-2-yl)methyl]phenyl}-2-(trifluoromethyl)benzamide (**2g**)

^{1}H NMR

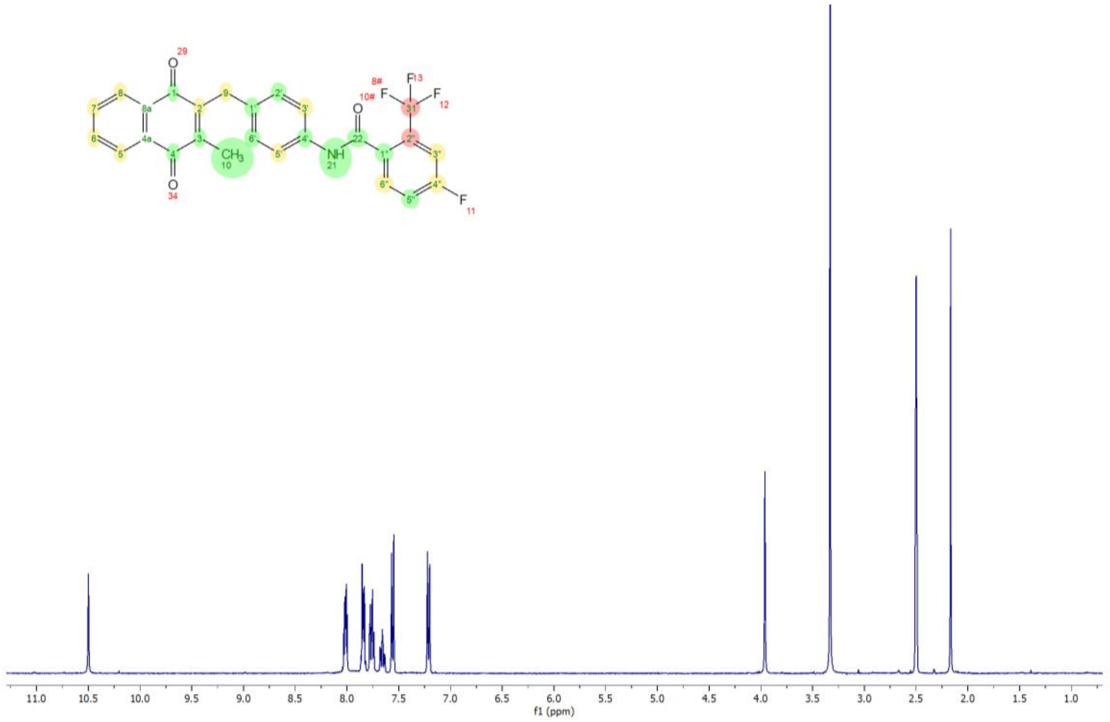

^{13}C NMR

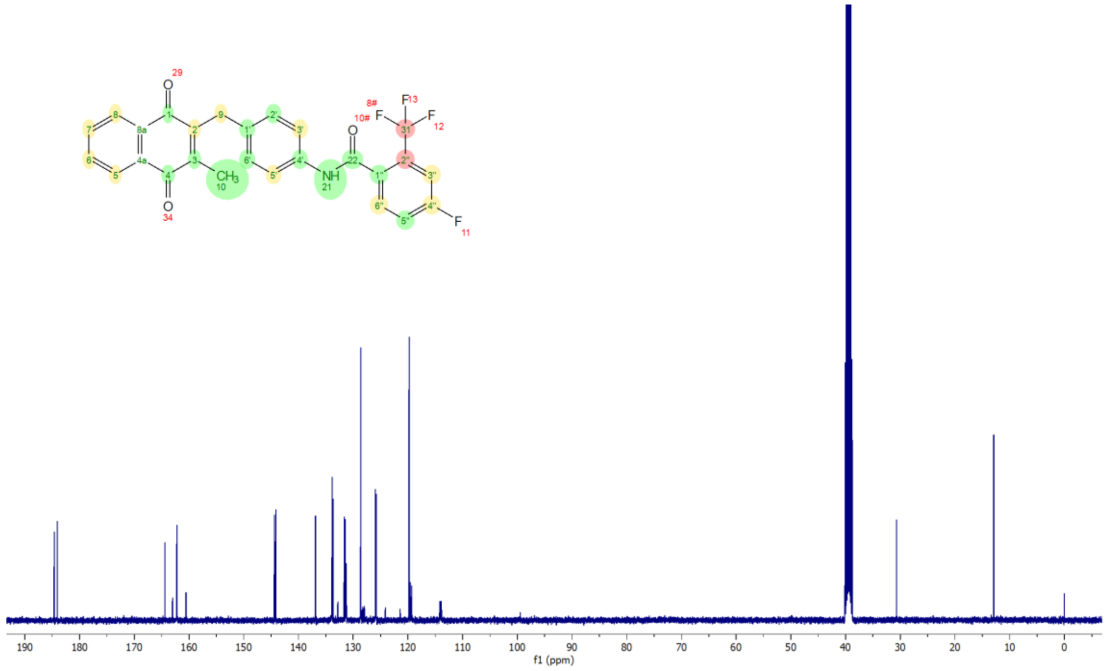

N-{4-[(3-Methyl-1,4-dioxo-1,4-dihydronaphthalen-2-yl)methyl]phenyl}-3-(trifluoromethoxy)benzamide (**2h**)

^{1}H NMR

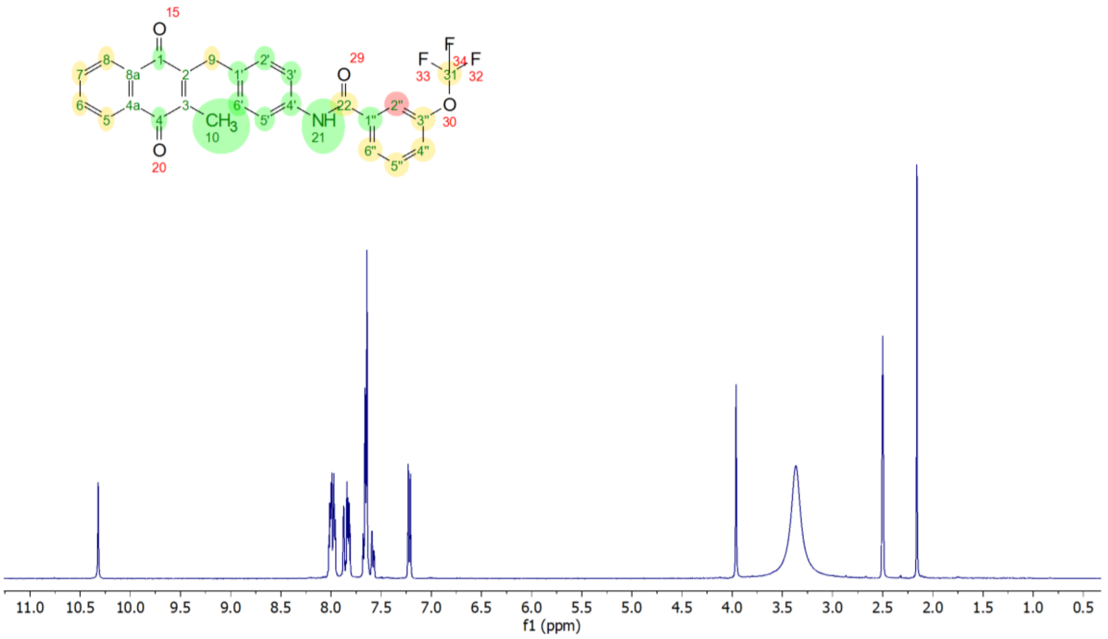

^{13}C NMR

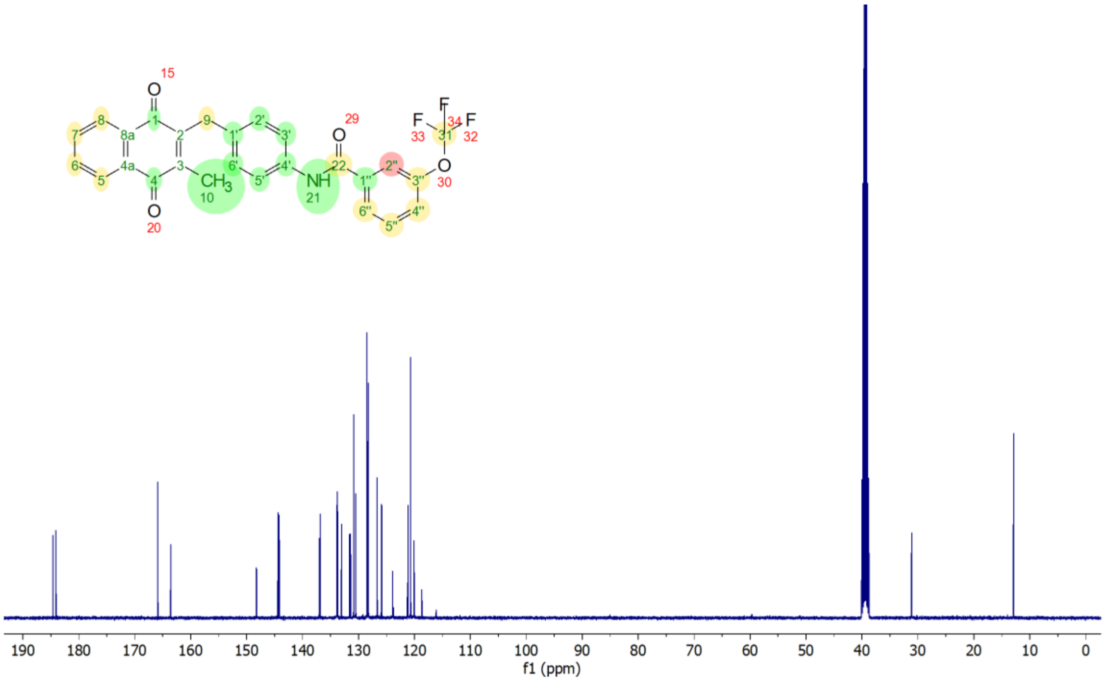

2,4,5-Trifluoro-N-{4-[(3-methyl-1,4-dioxo-1,4-dihydronaphthalen-2-yl)methyl]phenyl}benzamide (**2i**)

^{1}H NMR

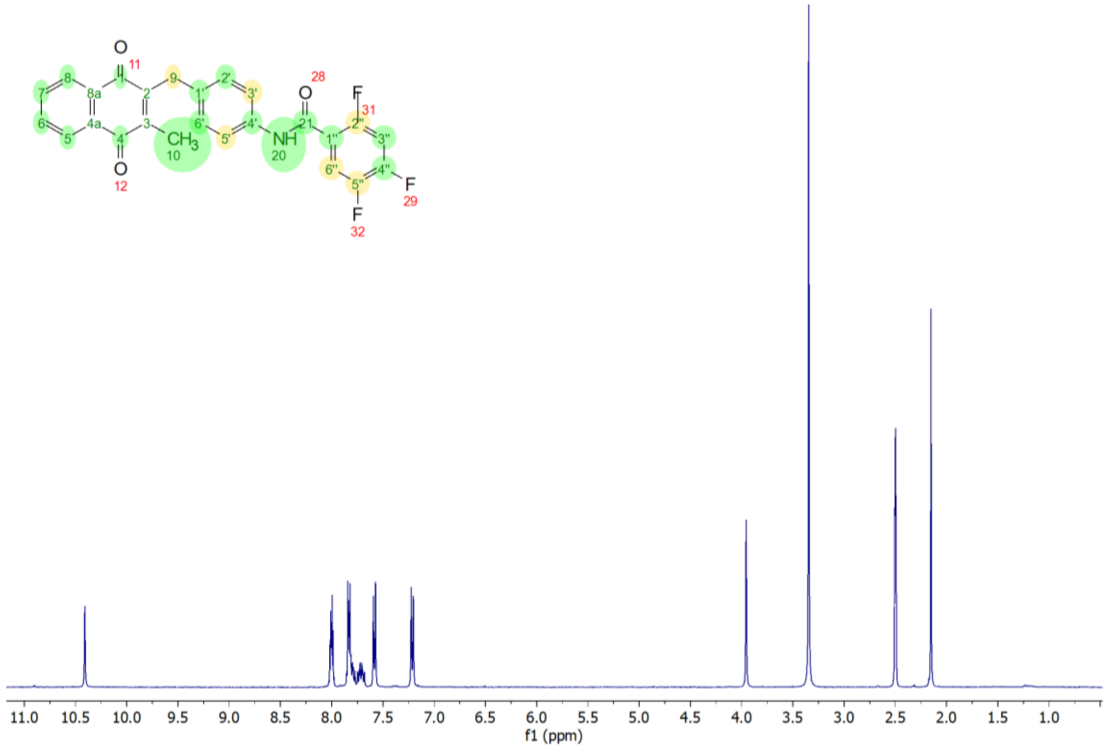

^{13}C NMR

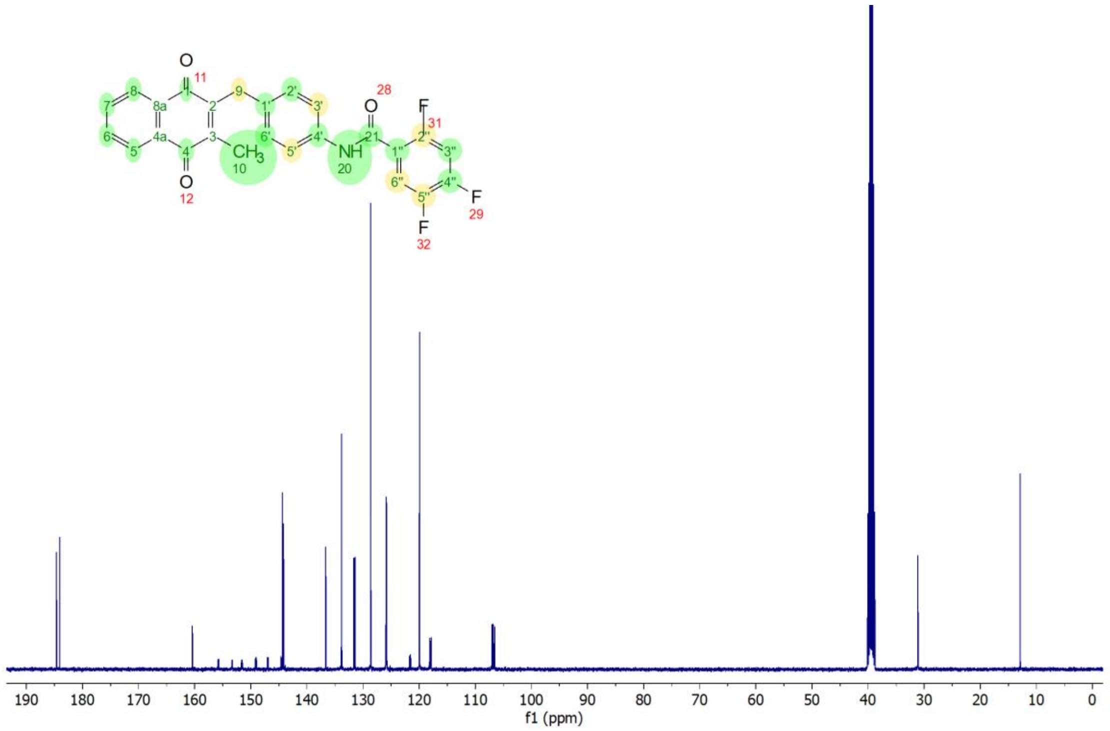

N-{3-[(3-Methyl-1,4-dioxo-1,4-dihydronaphthalen-2-yl)methyl]phenyl}benzamide (**3a**)

^{1}H NMR

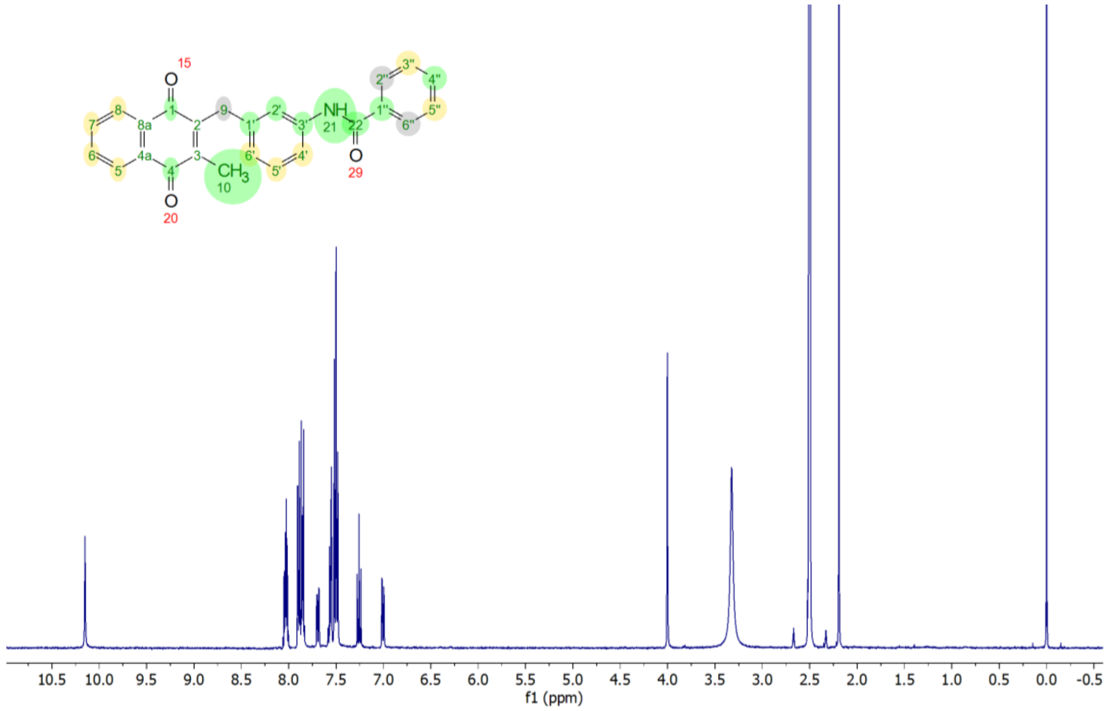

^{13}C NMR

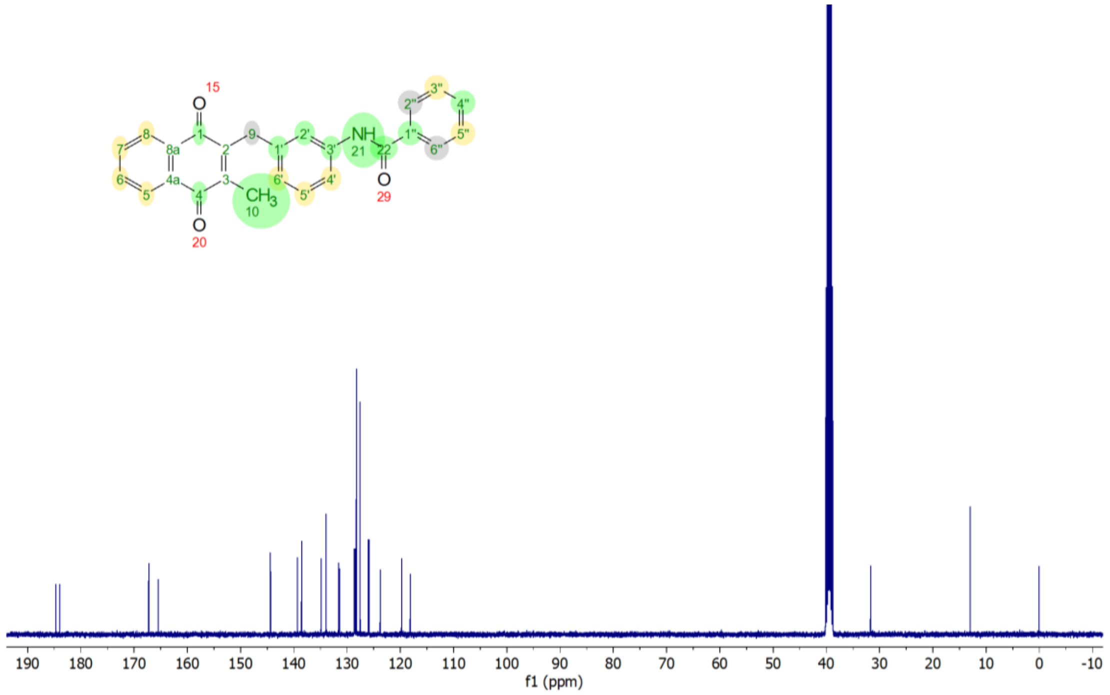

N-{3-[(3-Methyl-1,4-dioxo-1,4-dihydronaphthalen-2-yl)methyl]phenyl}-4-(trifluoromethyl)benzamide (**3b**)

^{1}H NMR

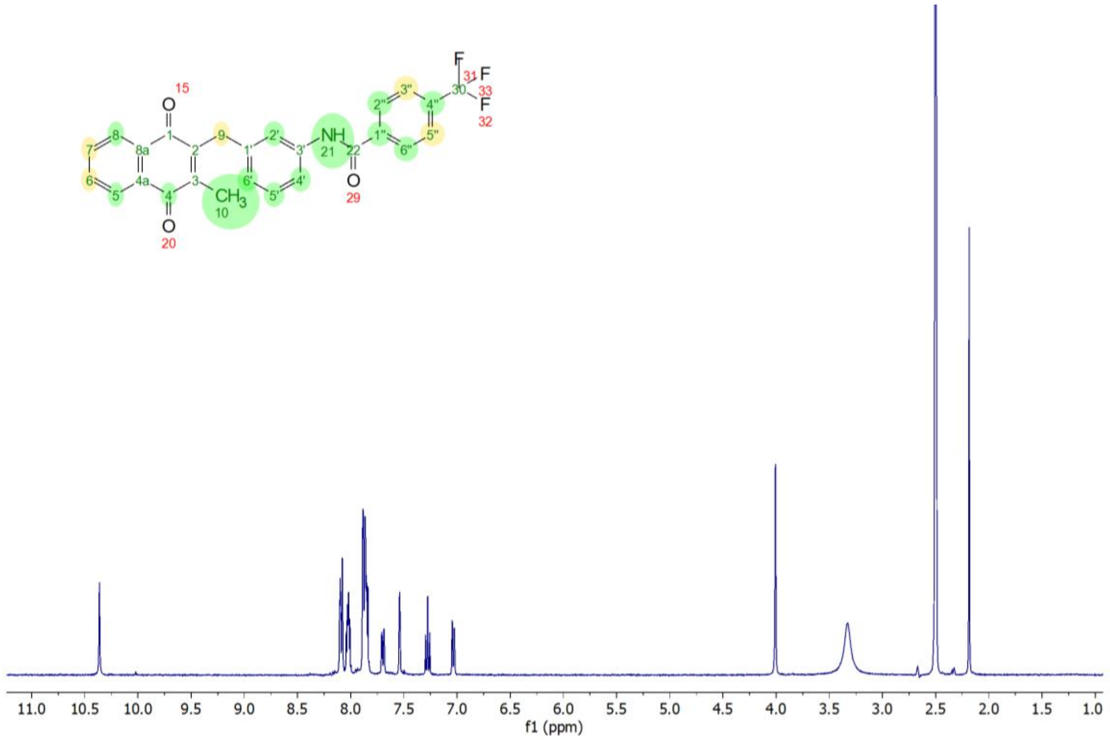

^{13}C NMR

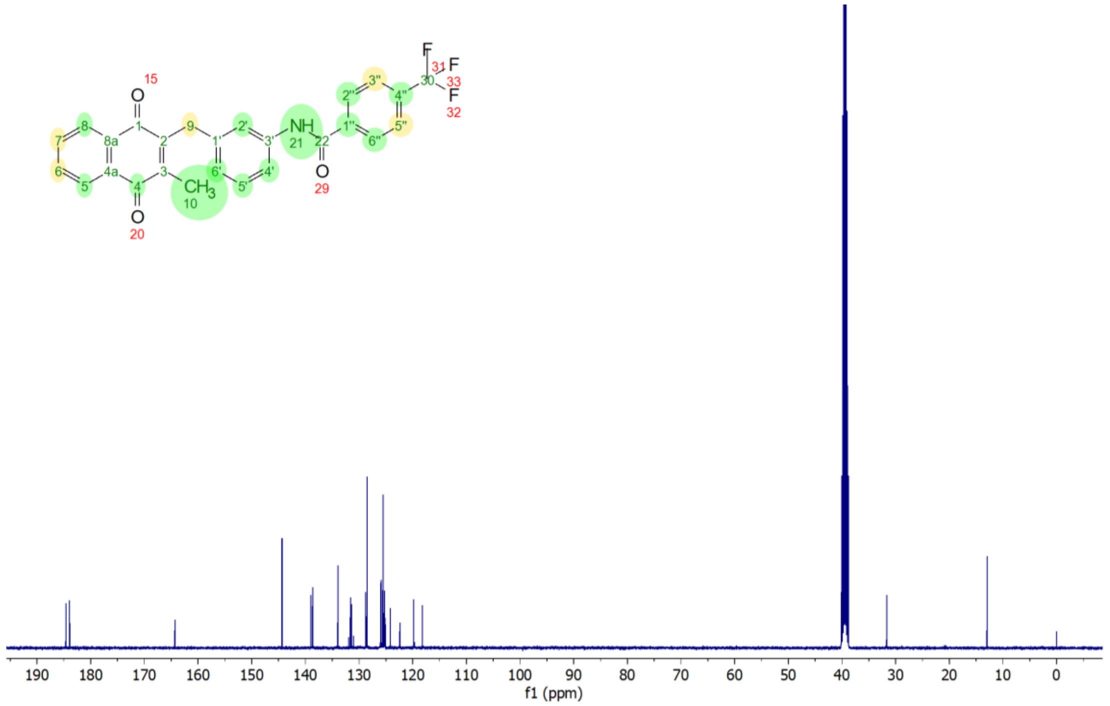

2-Fluoro-N-{3-[(3-methyl-1,4-dioxo-1,4-dihydronaphthalen-2-yl)methyl]phenyl}-4-(trifluoromethyl)benzamide (**3c**)

^{1}H NMR

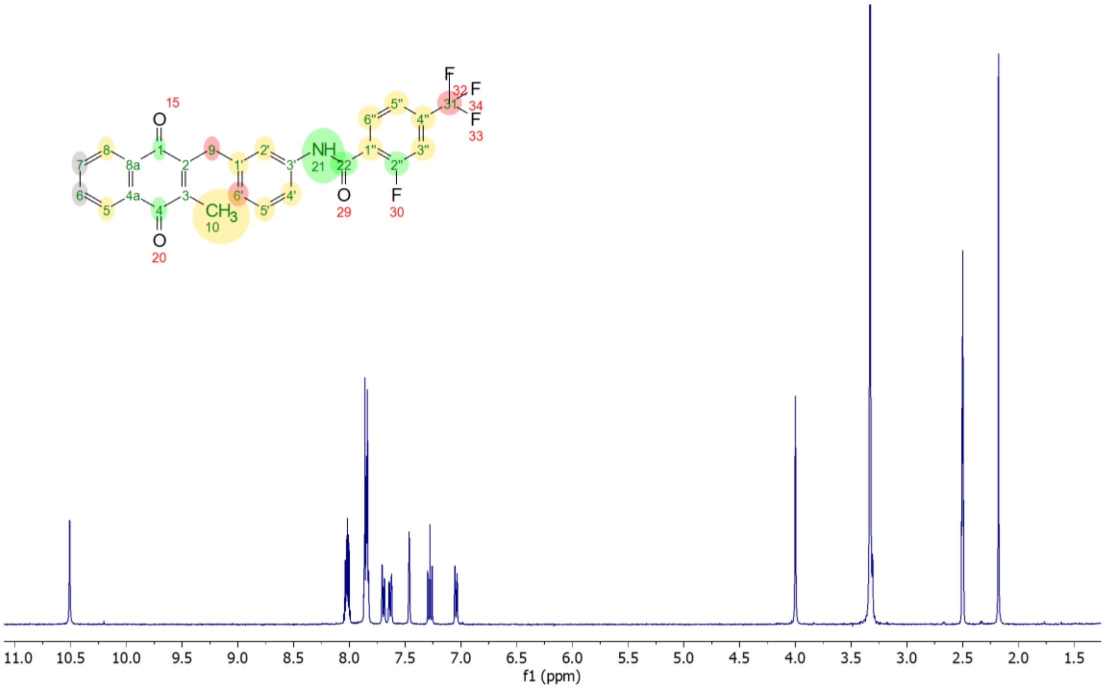

^{13}C NMR

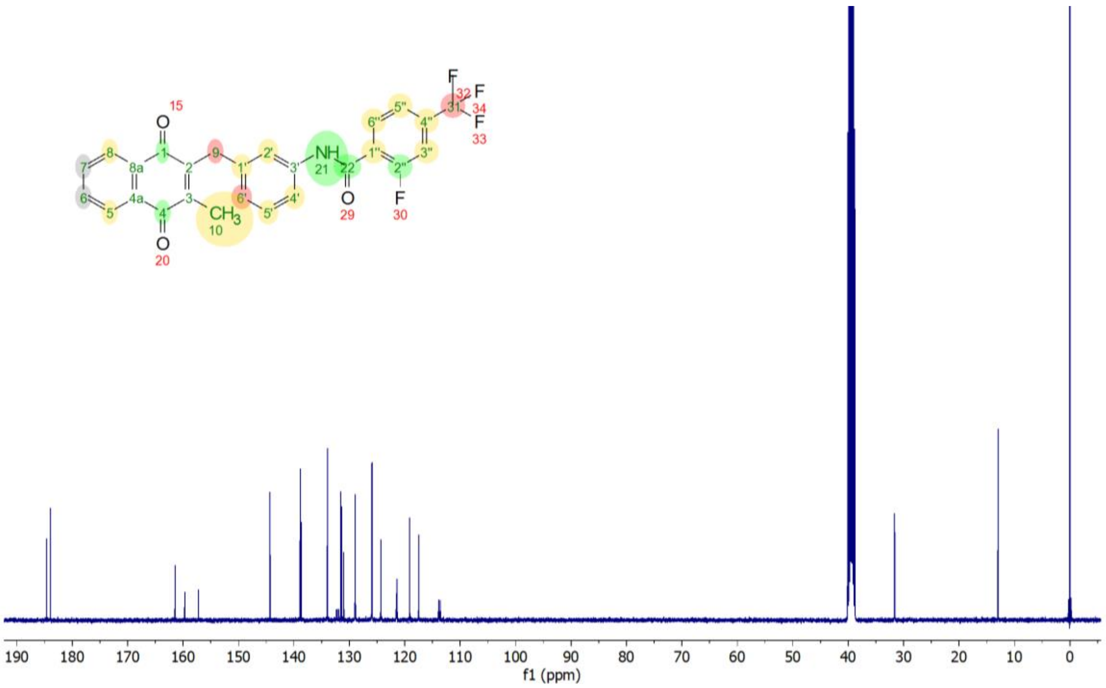

4-Fluoro-N-{3-[(3-methyl-1,4-dioxo-1,4-dihydronaphthalen-2-yl)methyl]phenyl}benzamide (**3d**)

^{1}H NMR

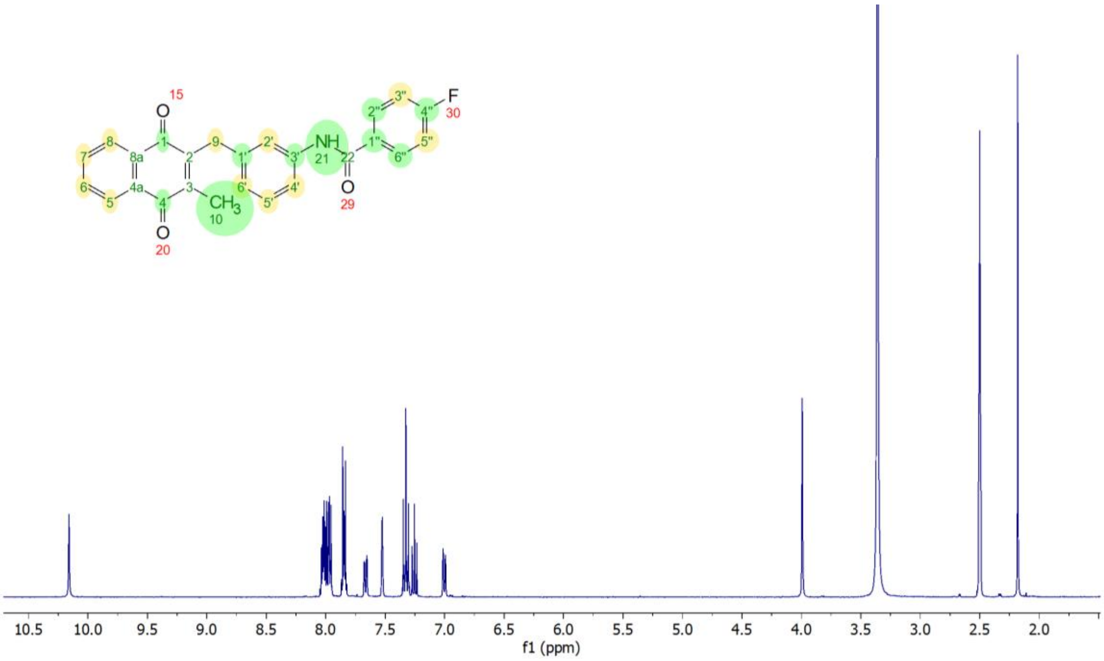

^{13}C NMR

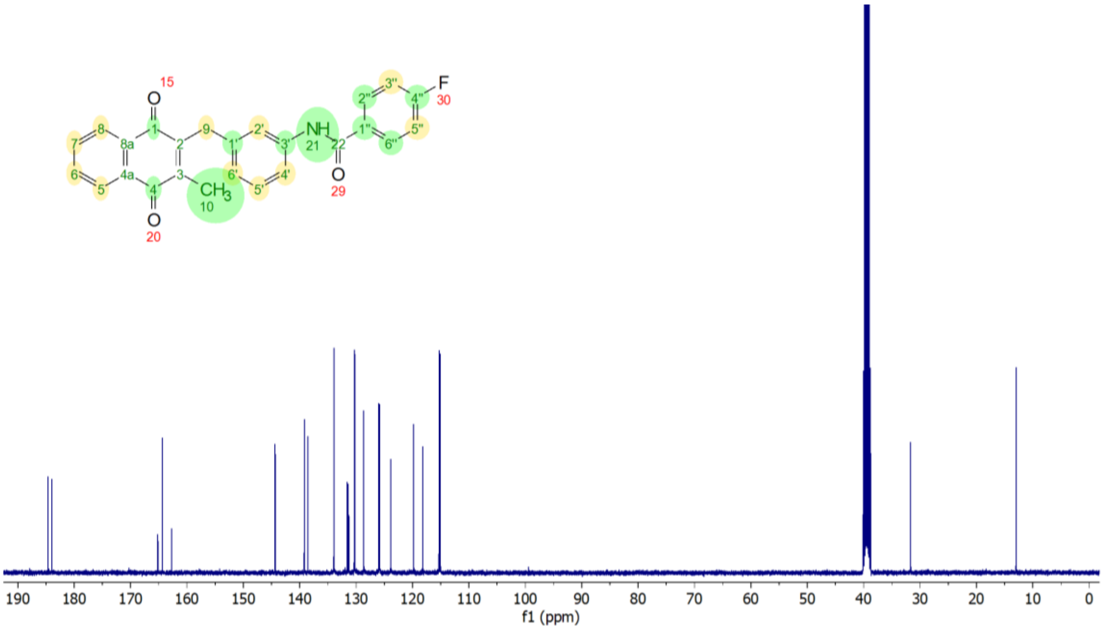

4-Fluoro-N-{3-[(3-methyl-1,4-dioxo-1,4-dihydronaphthalen-2-yl)methyl]phenyl}-3-(trifluoromethyl)benzamide (**3e**)

^{1}H NMR

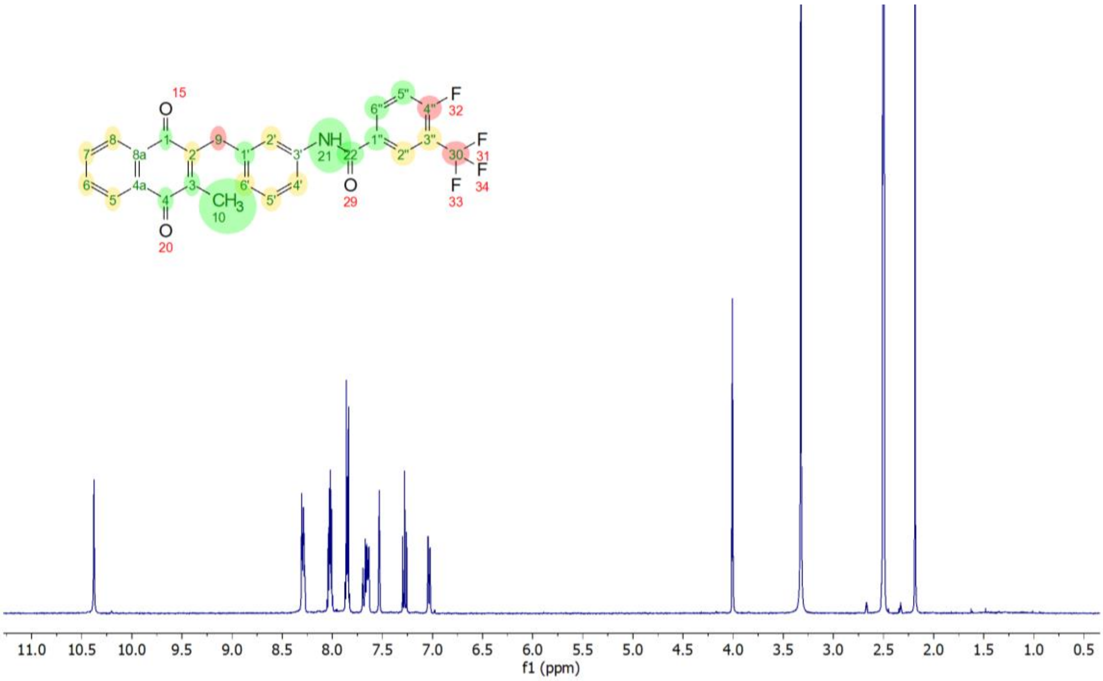

^{13}C NMR

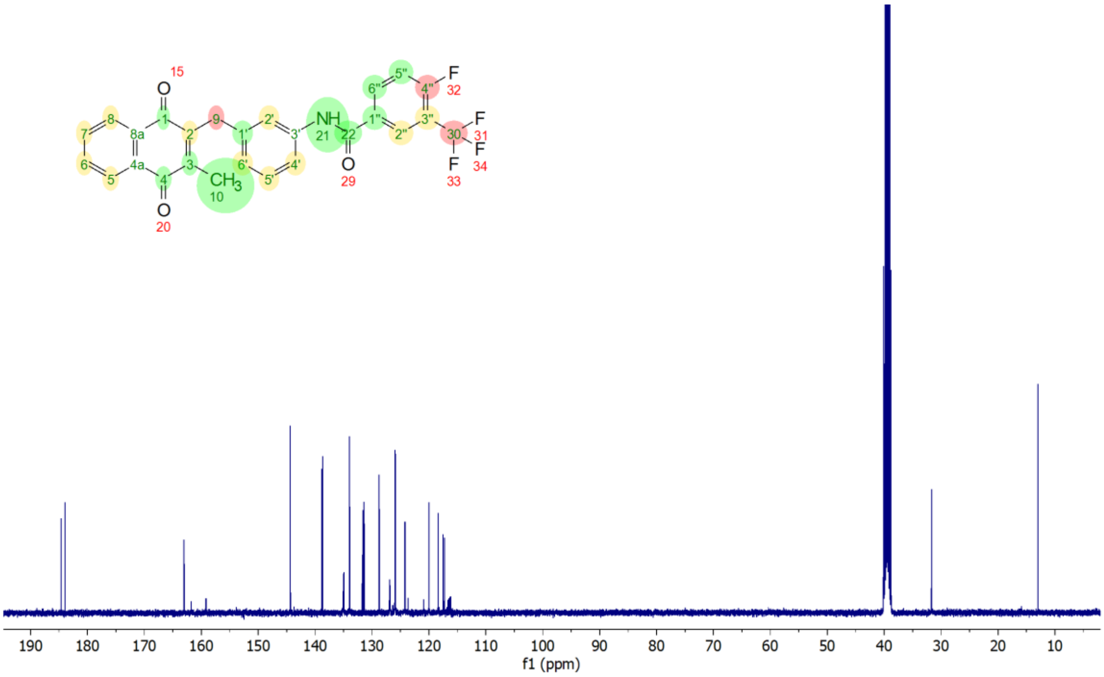

2-Fluoro-N-{3-[(3-methyl-1,4-dioxo-1,4-dihydronaphthalen-2-yl)methyl]phenyl}-5-(trifluoromethyl)benzamide (**3f**)

^{1}H NMR

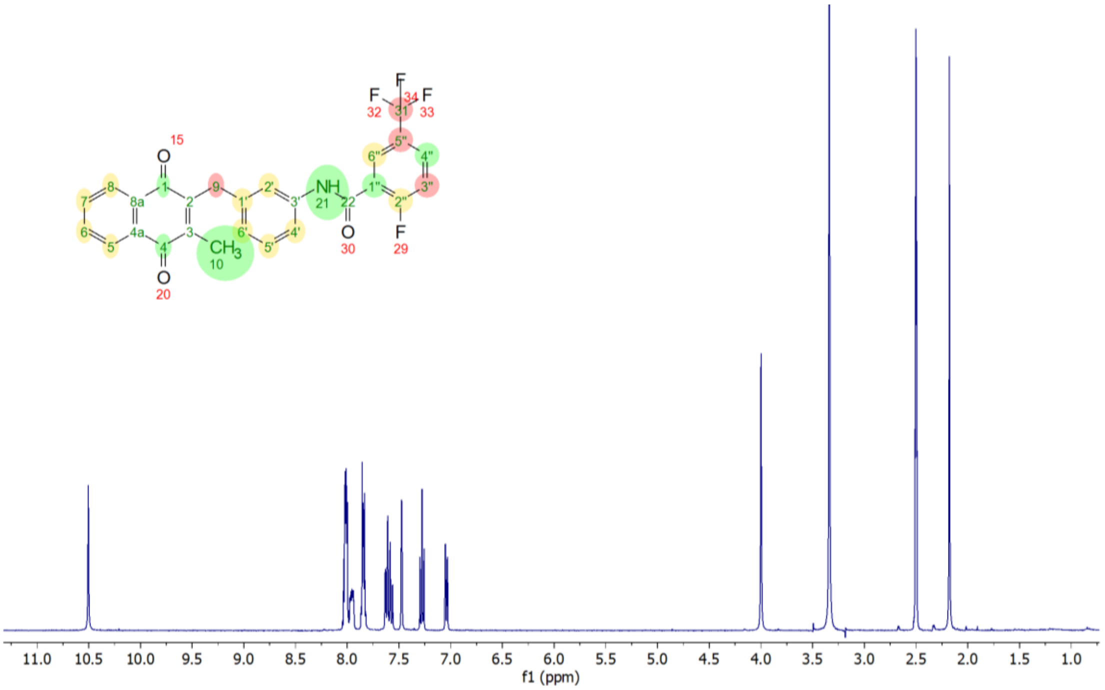

^{13}C NMR

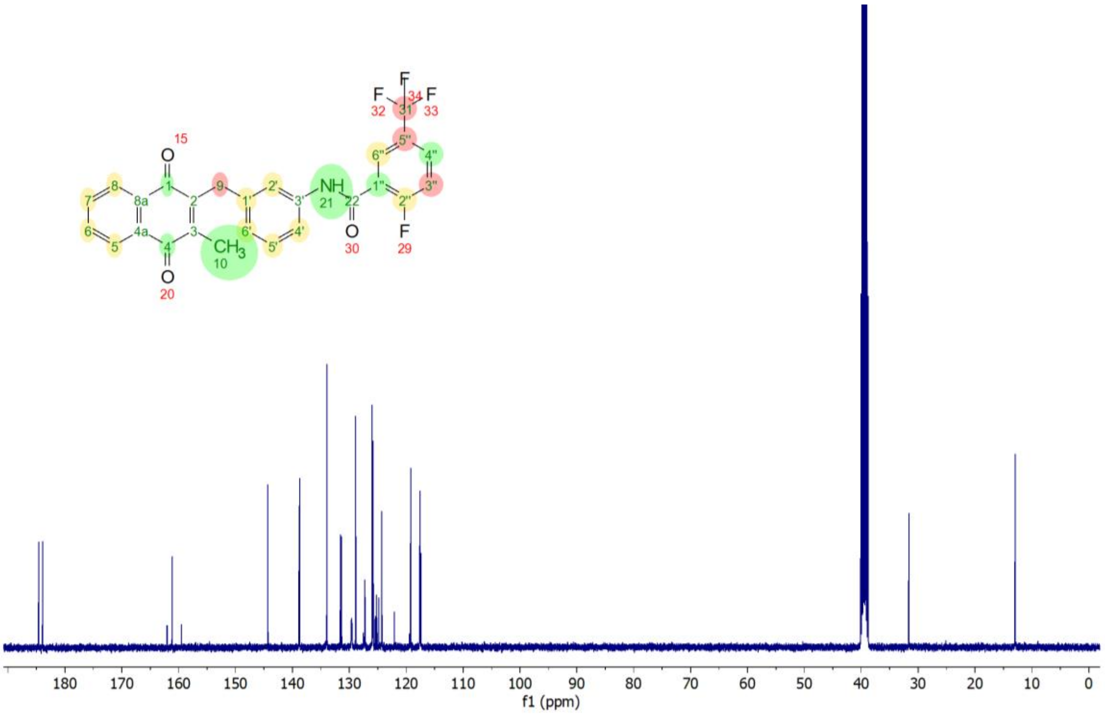

4-Fluoro-N-{3-[(3-methyl-1,4-dioxo-1,4-dihydronaphthalen-2-yl)methyl]phenyl}-2-(trifluoromethyl)benzamide (**3g**)

^{1}H NMR

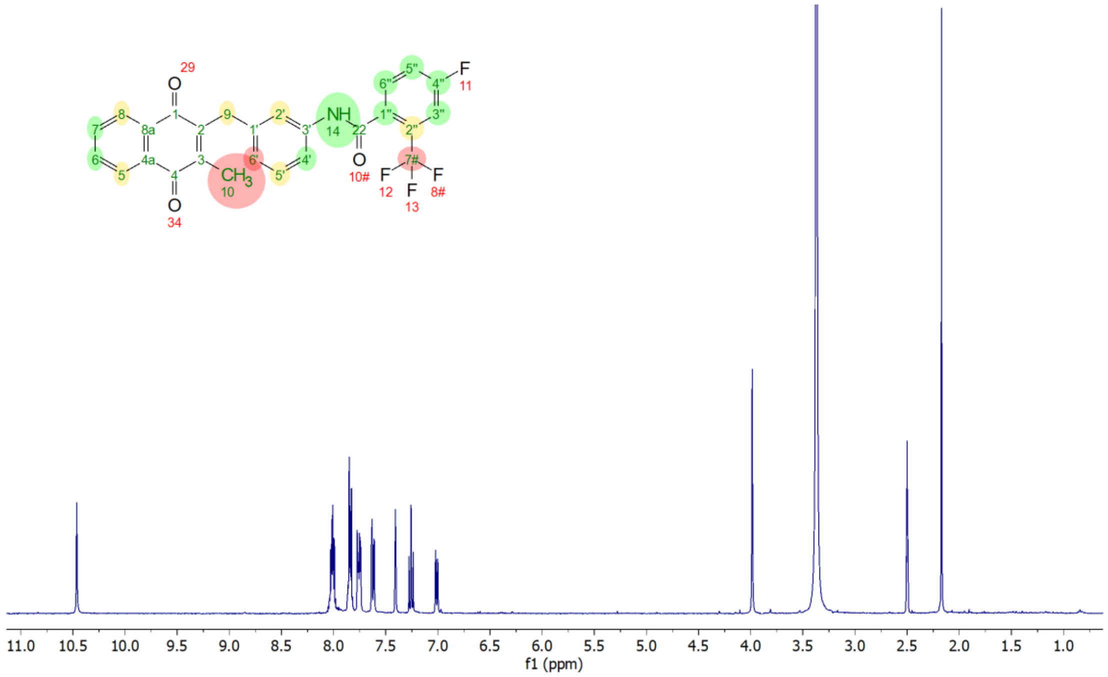

^{13}C NMR

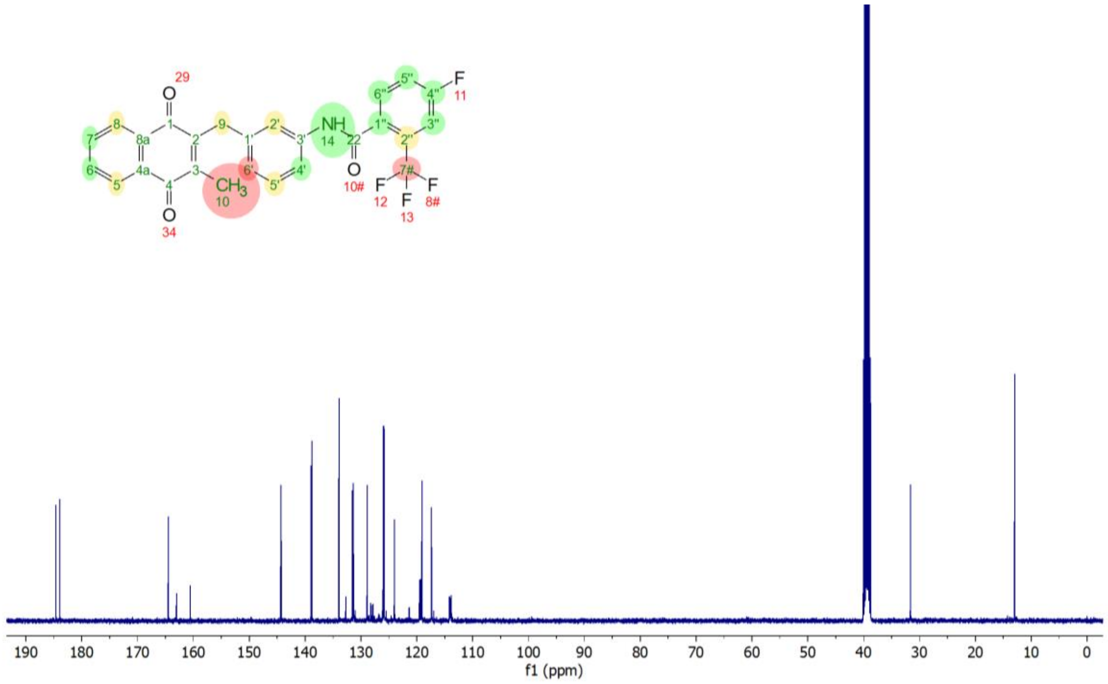

N-{3-[(3-Methyl-1,4-dioxo-1,4-dihydronaphthalen-2-yl)methyl]phenyl}-3-(trifluoromethoxy)benzamide (**3h**)

^{1}H NMR

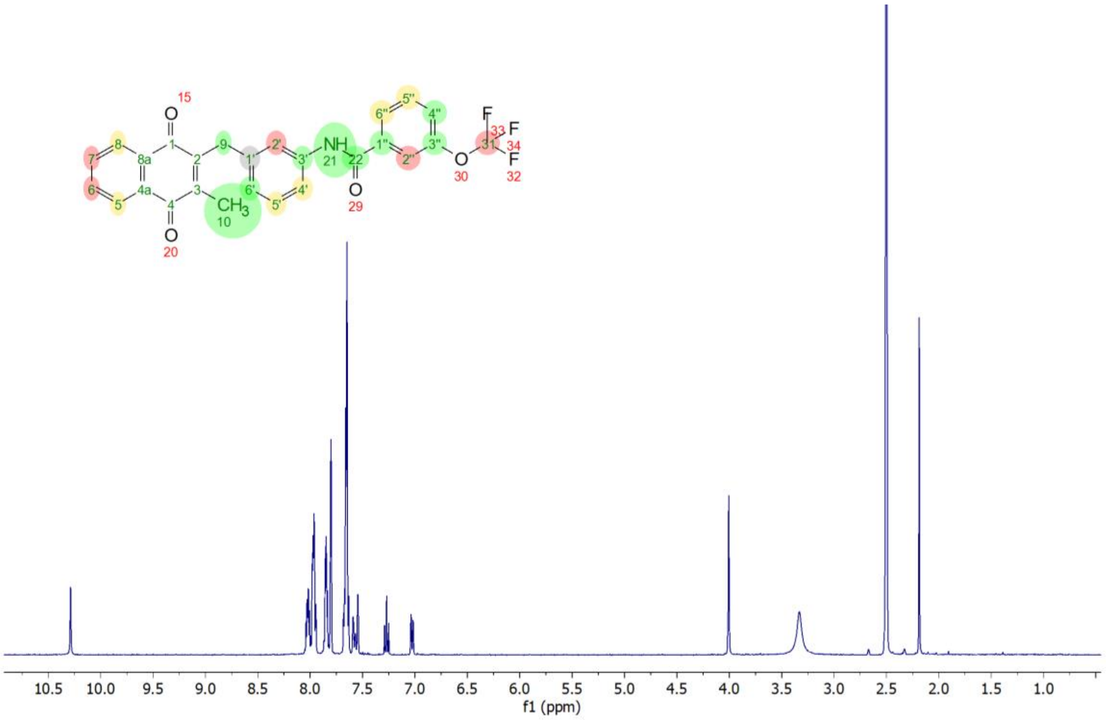

^{13}C NMR

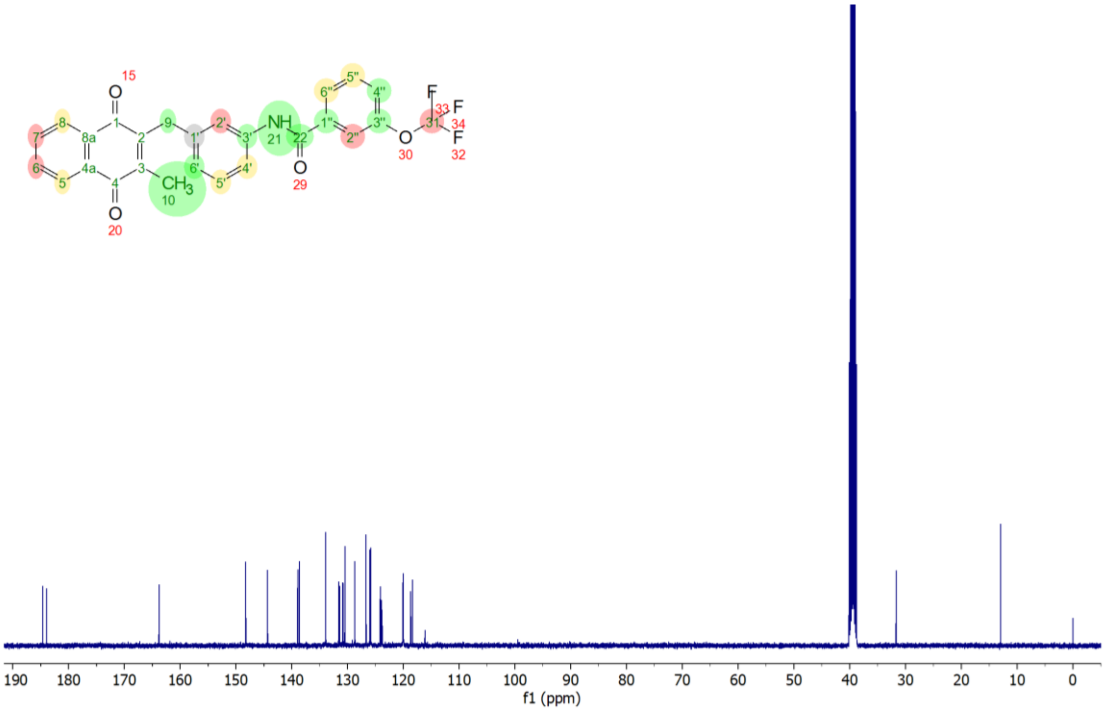

N-{4-[(1,4-Dioxo-1,4-dihydronaphthalen-2-yl)methyl]phenyl}benzamide (**5a**)

^{1}H NMR

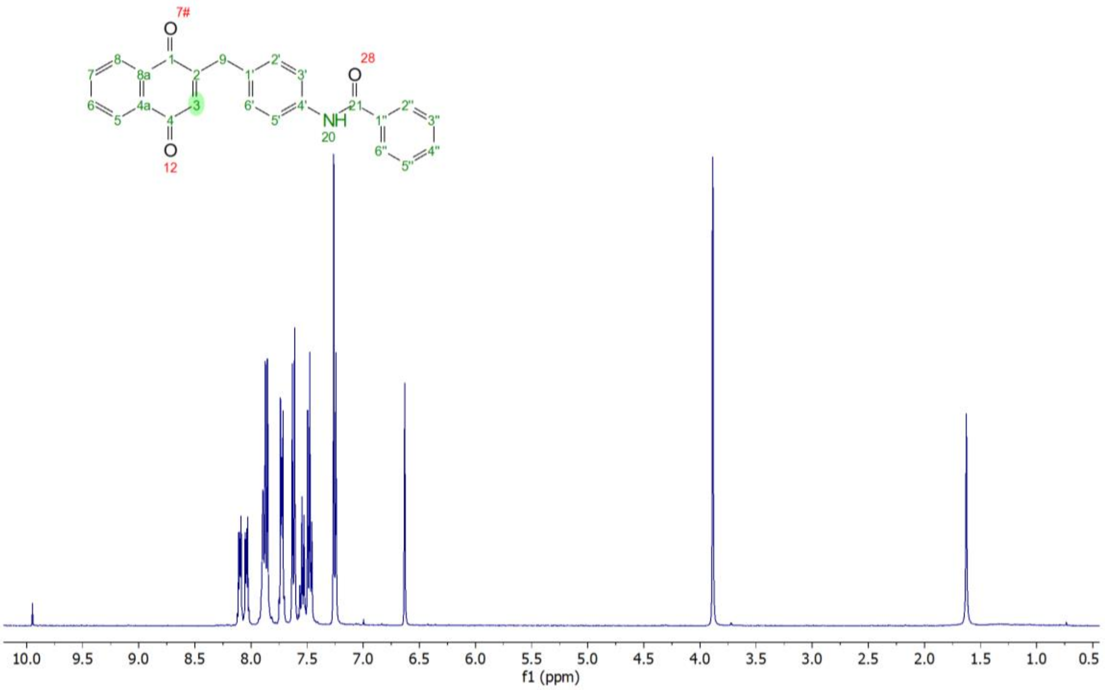

^{13}C NMR

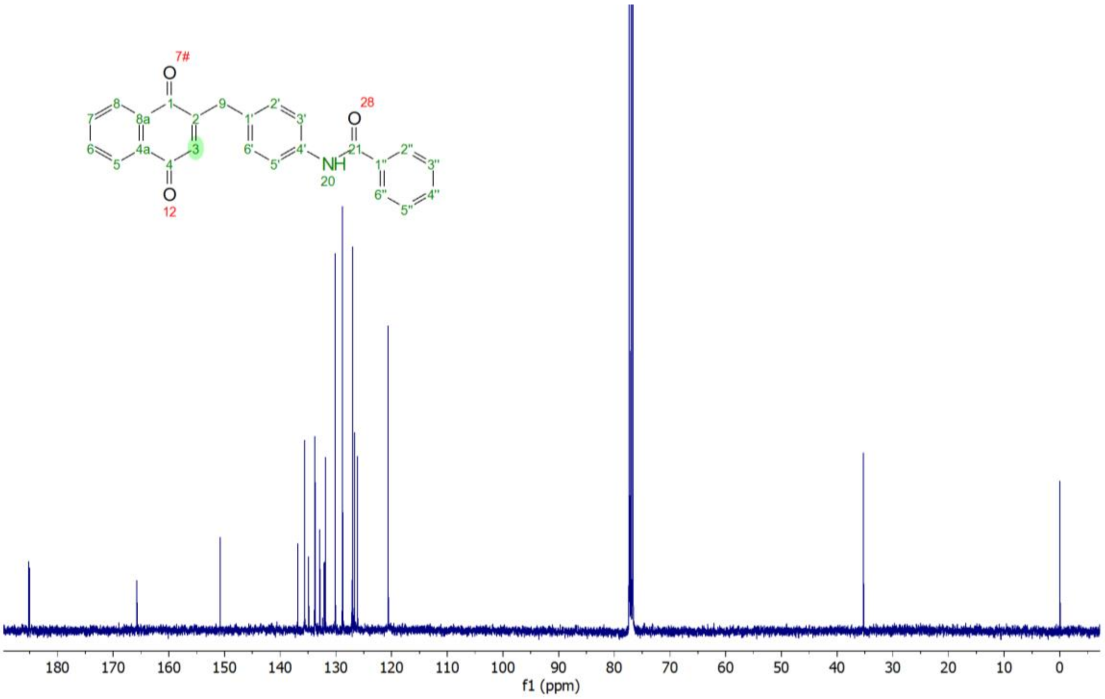

N-{4-[(1,4-Dioxo-1,4-dihydronaphthalen-2-yl)methyl]phenyl}-4-(trifluoromethyl)benzamide (**5b**)

^{1}H NMR

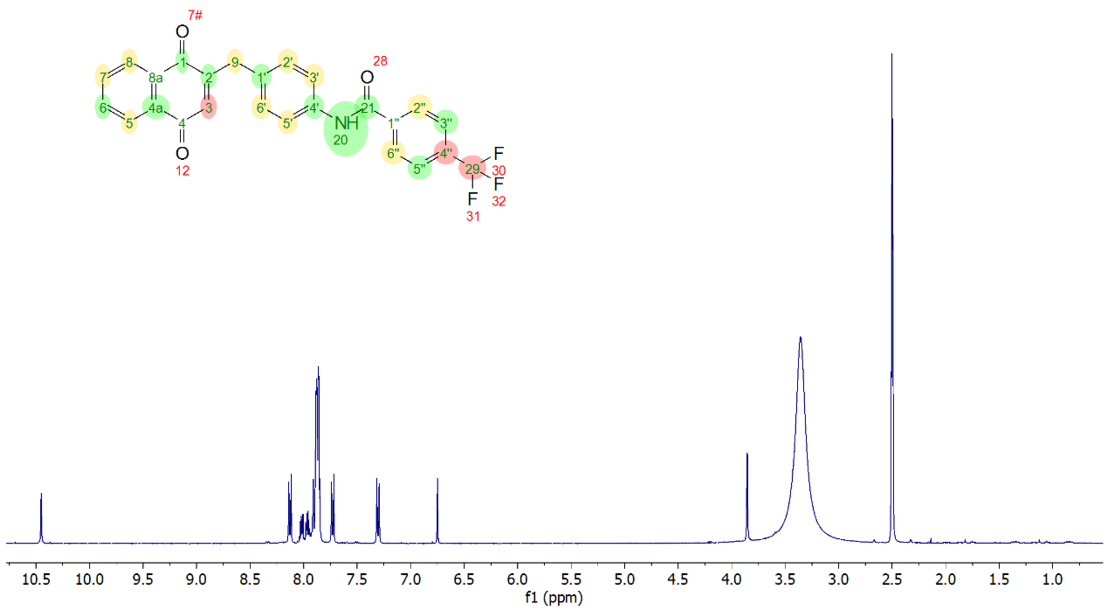

^{13}C NMR

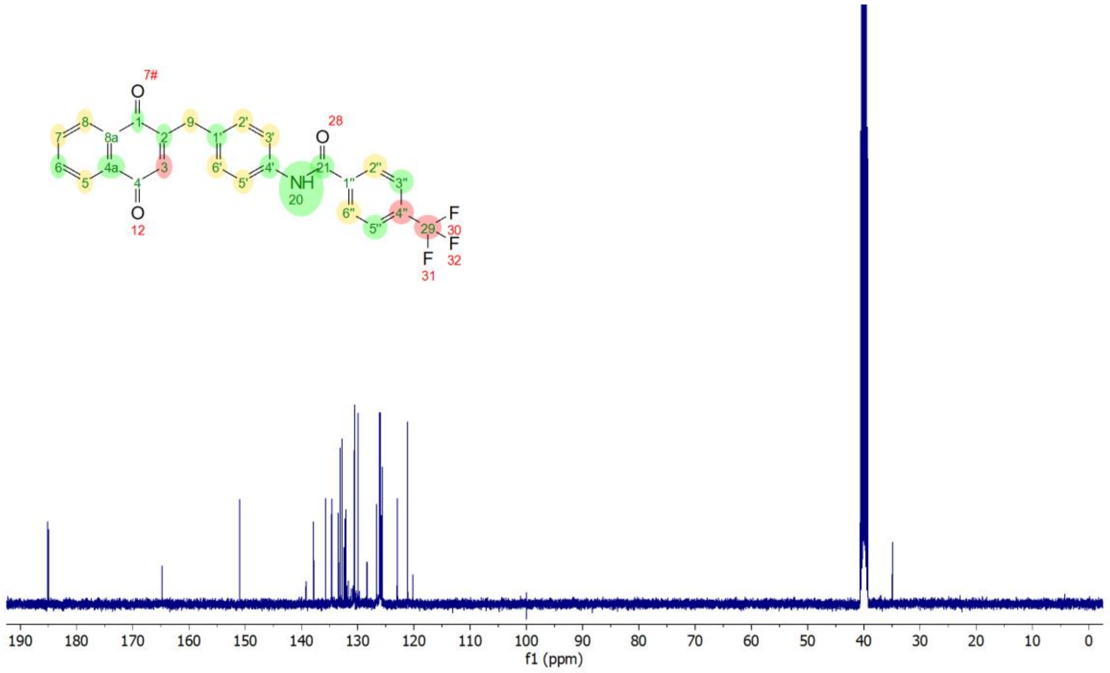

N-{3-[(1,4-Dioxo-1,4-dihydronaphthalen-2-yl)methyl]phenyl}benzamide (**6a**)

^{1}H NMR

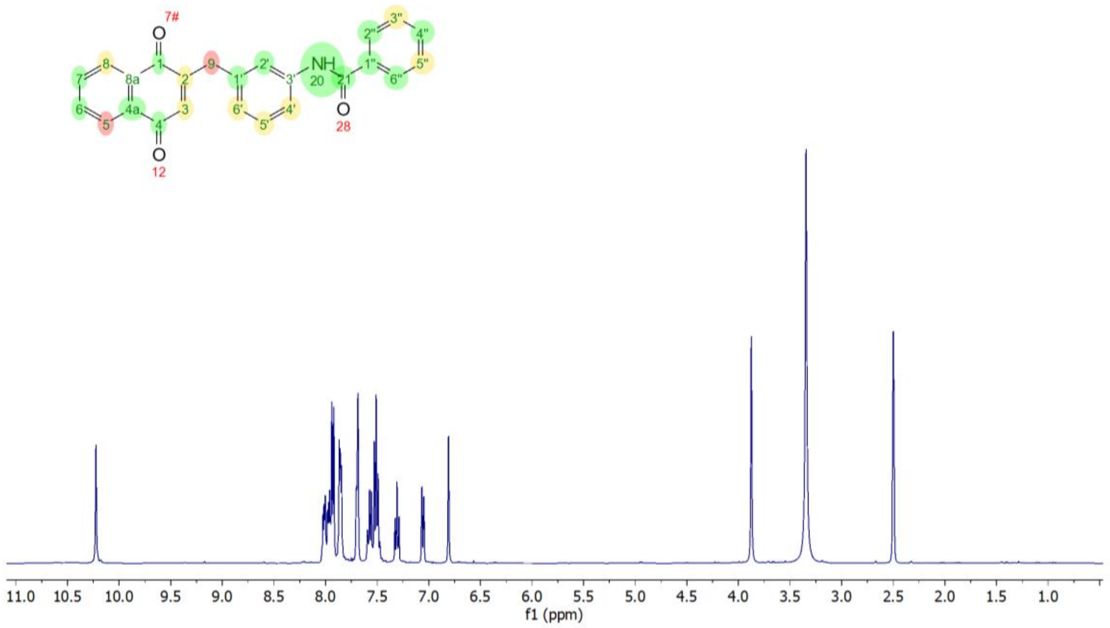

^{13}C NMR

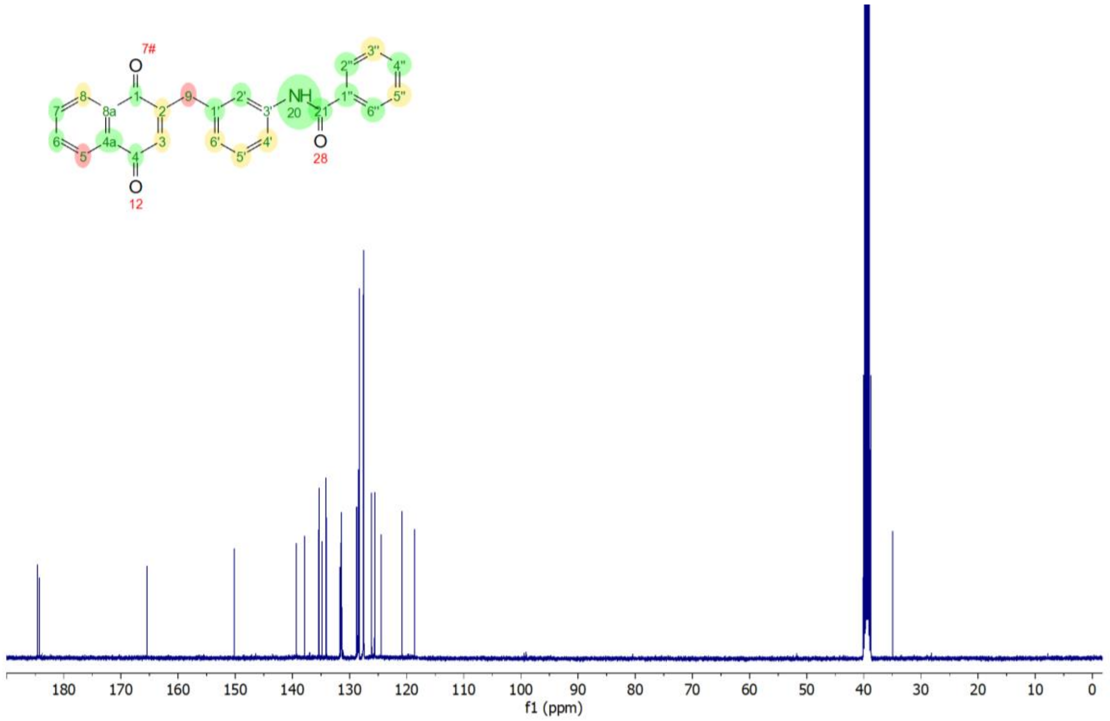

N-{3-[(1,4-Dioxo-1,4-dihydronaphthalen-2-yl)methyl]phenyl}-4-(trifluoromethyl)benzamide (**6b**)

^{1}H NMR

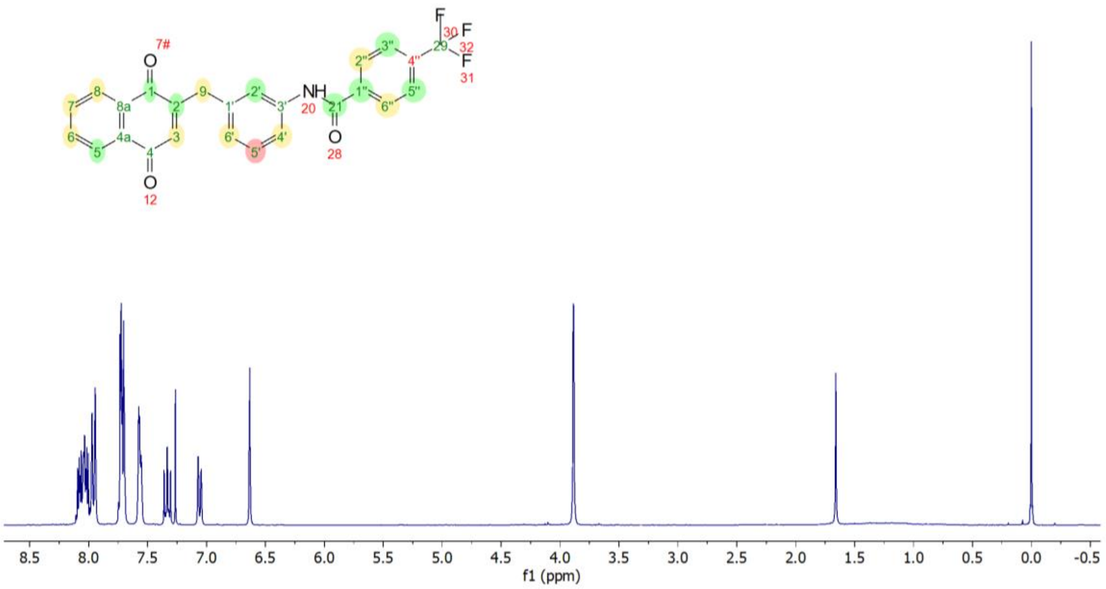

^{13}C NMR

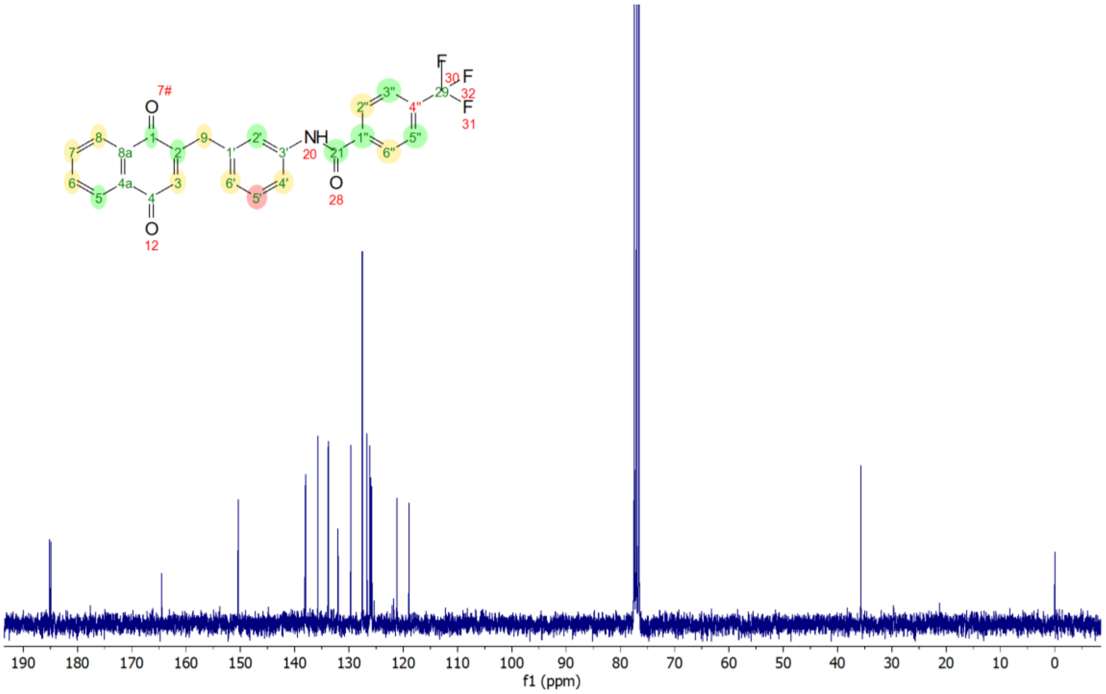

Supplement: Supplementary file 1 [file ijms-26-10951-s001.zip › ijms-3976285-supplementary.pdf]
